# Potent neutralizing nanobodies resist convergent circulating variants of SARS-CoV-2 by targeting diverse and conserved epitopes

**DOI:** 10.1038/s41467-021-24963-3

**Published:** 2021-08-03

**Authors:** Dapeng Sun, Zhe Sang, Yong Joon Kim, Yufei Xiang, Tomer Cohen, Anna K. Belford, Alexis Huet, James F. Conway, Ji Sun, Derek J. Taylor, Dina Schneidman-Duhovny, Cheng Zhang, Wei Huang, Yi Shi

**Affiliations:** 1 Department of Pharmacology and Chemical Biology, University of Pittsburgh, Pittsburgh, PA USA; 2 The University of Pittsburgh and Carnegie Mellon University Program for Computational Biology, Pittsburgh, PA USA; 3 Department of Cell Biology, University of Pittsburgh, Pittsburgh, PA USA; 4 Medical Scientist Training Program, University of Pittsburgh School of Medicine and Carnegie Mellon University, Pittsburgh, PA USA; 5 School of Computer Science and Engineering, Institute of Life Sciences, The Hebrew University of 6, Jerusalem, Israel; 6 Department of Structural Biology, University of Pittsburgh, Pittsburgh, PA USA; 7 Department of Structure Biology, St. Jude Children’s Research Hospital, Memphis, TN USA; 8 Department of Pharmacology, Case Western Reserve University, Cleveland, OH USA; 9 Department of Biochemistry, Case Western Reserve University, Cleveland, OH USA

**Keywords:** Cryoelectron microscopy, Microbiology, Structural biology

## Abstract

Interventions against variants of severe acute respiratory syndrome coronavirus 2 (SARS-CoV-2) are urgently needed. Stable and potent nanobodies (Nbs) that target the receptor binding domain (RBD) of SARS-CoV-2 spike are promising therapeutics. However, it is unknown if Nbs broadly neutralize circulating variants. We found that RBD Nbs are highly resistant to variants of concern (VOCs). High-resolution cryoelectron microscopy determination of eight Nb-bound structures reveals multiple potent neutralizing epitopes clustered into three classes: Class I targets ACE2-binding sites and disrupts host receptor binding. Class II binds highly conserved epitopes and retains activity against VOCs and RBD_SARS-CoV_. Cass III recognizes unique epitopes that are likely inaccessible to antibodies. Systematic comparisons of neutralizing antibodies and Nbs provided insights into how Nbs target the spike to achieve high-affinity and broadly neutralizing activity. Structure-function analysis of Nbs indicates a variety of antiviral mechanisms. Our study may guide the rational design of pan-coronavirus vaccines and therapeutics.

## Abstract (web summary)

Highly potent neutralizing nanobodies (Nbs) are of great interest as potential COVID-19 therapeutics. Here, the authors show that potent neutralizing Nbs targeting the receptor binding domain (RBD) of the SARS-CoV-2 spike protein are also effective against convergent variants of concern of the virus. They determine eight Nb-bound spike protein cryo-EM structures, classify the binding epitopes of the Nbs and discuss their neutralization mechanisms.

## Introduction

The Coronavirus Disease 2019 (COVID-19) pandemic has caused devastating consequences to global health and the economy. In addition to vaccine development, potent neutralizing monoclonal antibodies (mAbs) isolated from convalescent plasma^1^ has been approved for emergency therapeutic use with more candidates in the pipeline^2–4^. Camelid VHH single-domain antibodies or nanobodies (Nbs) have also been successfully developed for virus neutralization^5–12^. By using camelid immunization with receptor-binding domain (RBD)_SARS-CoV2_ and an advanced proteomic pipeline, we have recently identified thousands of high-affinity RBD Nbs including a repertoire of ultrapotent Nbs^8^. Affinity-matured Nbs are highly soluble, stable, and can be rapidly produced in microbes at low costs^13^. Stable Nbs can resist aerosolization for inhalation therapy of SARS-CoV-2 infection. Recently, the high preclinical efficacy of an ultrapotent Nb construct (PiN-21) has been demonstrated in a sensitive COVID-19 animal model. At a very low dose (0.2 mg/kg), the PiN-21 inhalation treatment quickly protects animals’ weight loss after SARS-CoV-2 infection, decreases lung viral titers by a million fold which leads to drastically mitigated lung pathology, while preventing viral pneumonia^14^. Potent neutralizing Nbs, therefore, represent a convenient and cost-effective therapeutic option to help mitigate the evolving pandemic.

The ACE2 receptor binding site (RBS) on the spike glycoprotein (S) is the major target of serologic response in COVID-19 patients. The RBS is the primary region of convergent mutations in circulating variants of SARS-CoV-2. The variants may enhance ACE2 binding leading to higher transmissibility, elude clinical mAbs, and reduce the neutralizing activities of both convalescent and vaccine-elicited polyclonal sera^15–17^. Particularly concerning variants include Alpha (B.1.1.7), Beta (B.1.351), and Gamma (P.1)^18–20^. Long-term control of the pandemic will require the development of effective interventions with broadly neutralizing activities against the evolving strains^18^.

Here, we assessed the impact of the convergent variants of concern and the critical RBD point mutations on the ultrapotent neutralizing Nbs. Subsequent determination of eight high-resolution structures, involving six Nbs bound to either S or RBD, by cryo-EM provided critical insights into the antiviral mechanisms of highly potent neutralizing Nbs. Structural comparisons between neutralizing mAbs and Nbs revealed both similarities and marked differences between the two antibody species.

## Results

### Potent Nbs are resistant to circulating SARS-CoV-2 variants

**Fig. 1:**
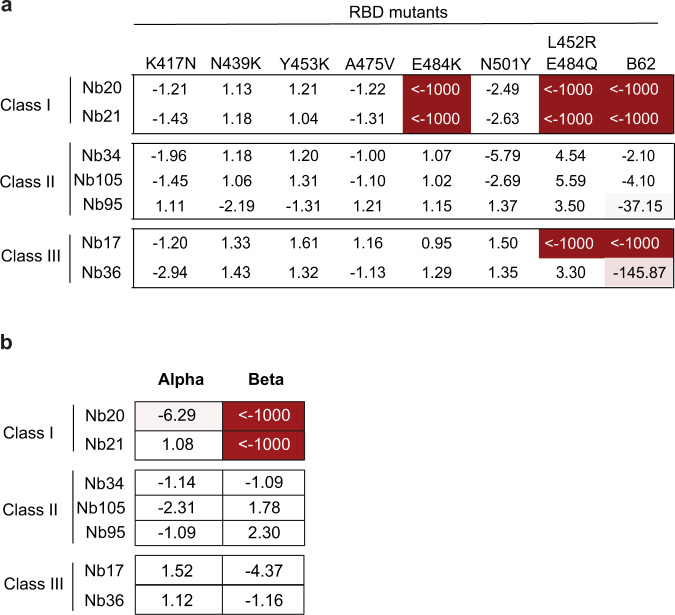
The impact of RBD circulating variants on Nb binding and neutralization. **a** ELISA binding of the spike variants (a summary heatmap). Data are shown as binding affinity fold change relative to that of RBD WT. **b** The fold change in neutralizing potencies of the Nbs against two dominant circulating variants (Alpha and Beta strains) relative to that of the wild-type SARS-CoV-2 pseudovirus particles. Negative values represent a loss in affinity or neutralization potency, and positive values represent a gain in affinity or neutralization potency. Based on the highest Nb concentration tested, reduction in affinity or neutralization potency greater than 1000-fold is represented as “<−1000”.

We performed enzyme-linked immunosorbent assay (ELISA) to evaluate how six critical RBD mutations (Supplementary Table 1) impact the binding of seven diverse and potent neutralizing Nbs^8^. Interestingly, the neutralizing Nbs were generally unaffected by the mutations (Fig. 1a, Supplementary Fig. 1). An exception was E484K, which abolished the ultra-high affinities of Nbs 20 and 21. In addition, we found that three Nbs (17, 20, and 21) were affected by the double mutant L452R and E484Q, which are the critical RBD mutations recently found in Kappa (B.1.617.1) strain^21^. Moreover, we evaluated two circulating variants of global concern (Alpha and Beta) on Nb neutralization using a pseudotyped virus neutralization assay (Methods). These pseudoviruses fully recapitulate the major mutations of the natural spike variants (Supplementary Fig. 2, Methods). The initial SARS-CoV-2 strain (Wuhan-Hu-1) was used as a control. Consistent with the ELISA results, we found that the UK strain (Alpha), possessing a critical RBD mutation N501Y, has little effect on all 7 potent neutralizing Nbs (Fig. 1b, Supplementary Fig. 3). The Beta strain (B.1.351), containing three RBD mutations (K417N, E484K, and N501Y), drastically reduces the efficacy of Nbs 20 and 21 but has a very marginal impact on the efficacies of other Nbs. The results contrast with recent investigations of a repertoire of neutralizing mAbs derived from convalescent and vaccine-elicited polyclonal sera, where most of the isolated mAbs were significantly affected by at least one of mutations found in VOCs^22^.

To assess the potential to resist future mutations, highly neutralizing Nbs were evaluated for their binding to a potential future RBD variant (B62). B62 possesses unseen mutations which were evolved in vitro for high ACE2 binding affinity and potentially enhanced infectivity (Supplementary Fig. 2). This highly evolved RBD variant contains 9 point mutations (I358F, V445K, N460K, I468T, T470M, S477N, E484K, Q498R, and N501Y), including both established and potential mutations that together increase the affinity of ACE2 binding by 600-fold^23^. While several Nbs were substantially affected by B62, Nbs 34 and 105 retained their high affinity against this evolved variant. These striking results prompted us to further investigate the structural basis for the broadly neutralizing activities of these Nbs. Our high-resolution cryo-EM maps of Nbs in complex with either the prefusion-stabilized S^24^ or the RBD revealed three Nb classes that were affected differently by the variants and provided insights into their antiviral mechanisms, which were further supported by functional assays.

### Ultrapotent Class I Nbs and E484K as an Achilles’ heel

Class I dominates high-affinity RBD Nbs and represents some of the most potent neutralizers for SARS-CoV-2. For example, Nb21 can neutralize a clinical isolate of SARS-CoV-2 at sub-ng/ml, which is the most potent antiviral Nb reported to date. To better understand the neutralization mechanism of class I Nbs in the context of the S trimer, we solved the structure of Nb21 bound to the S using cryo-EM.

**Fig. 2:**
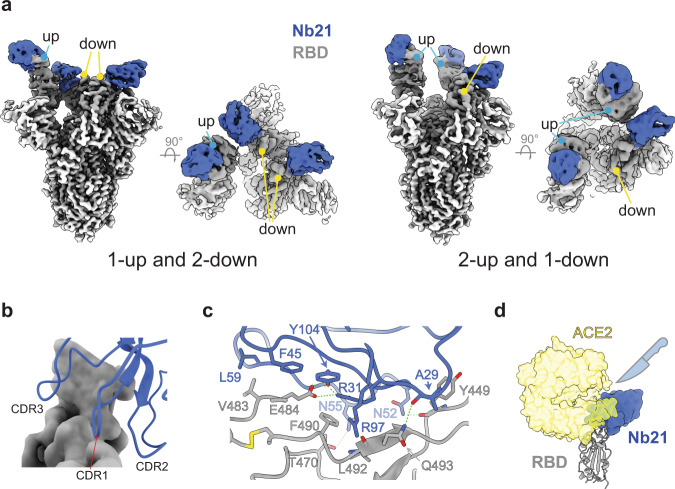
Structure of an ultrapotent class I Nb (21). **a** Cryo-EM structure of the Nb21:S complex reveals 1-up and 2-down RBD conformations. **b** The involvement of three CDRs of Nb21 for RBD binding. **c** Additional Nb21:RBD interactions: side chains of R97, N52, and N55 (Nb21) form hydrogen bonds with the main chain carbonyl groups of L492 and Y449 and the side chain of T470 (RBD), respectively. The main-chain carbonyl group of A29 (Nb21) also forms a hydrogen bond with Q493 (RBD). Besides these polar interactions, F45 and L59 of Nb21 and V483 of RBD form a cluster of hydrophobic interactions, together, providing ultrahigh-affinity and selectivity for RBD binding. **d** Structural overlap of hACE2 with Nb21:RBD complex.

Nb21 binds RBDs in both up and down conformations. There are two major classes of the spike bound to Nb21: (i) one up-RBD and two down-RBDs (with the overall resolution of 3.6 Å) and (ii) two up-RBDs and one down-RBD (3.9 Å) (Fig. 2a, Supplementary Fig. 4a–c). Due to the high flexibility of RBD, we performed local refinement of one down-RBD with Nb21 to resolve the binding interface (Supplementary Fig. 4c). Nb21 binds the extended external loop region of the RBD with two β-strands. The interactions are mediated by all three CDR loops (Fig. 2b). The local density map suggests potential cation-π interactions between R31 of Nb21 and F490 of RBD and a polar interaction network among R31 and Y104 of Nb21 and E484 of RBD. These four residues are located at the center of the Nb21:RBD interface, constituting a major site of interactions (Fig. 2c, Supplementary Fig. 10a).

Consistent with the ELISA and pseudovirus assay results, relative binding energy calculation (Methods) revealed that E484 on the RBD is the Achilles’ heel of Nb21 (Supplementary Fig. 5a). E484 provides the highest binding energy among all the interface residues on the RBD. In addition, it facilitates a network of adjacent residues such as F490, F489, N487, Y486, and V483 to participate in Nb21 binding. The E484K mutation can substantially destabilize the interface packing by electrostatic repulsion with R31 (CDR1), subsequently disrupting the cation-π stacking interaction between R31 and F490 (RBD). Simple charge reversal on R31 (R31D) failed to recover the salt bridge and binding to the E484K mutant (Supplementary Fig. 5b). Similarly, E484Q (on Kappa/B.1.617.1 variant), may also disrupt the critical salt bridge of E484: R31 and the neighboring interaction network resulting in substantial loss of binding to the RBD (Fig. 1).

In summary, Class I Nbs bind the RBS epitopes and can potently inhibit the virus by directly blocking ACE2 binding (Fig. 2d). Nevertheless, since the epitopes are among the least conserved regions on the spike, a critical point mutation (E484K/Q) can dramatically reduce the ultrahigh affinity of class I Nbs. The critical E484K/Q mutation is notably absent in the Alpha VOC, a variant that has once dominated infection cases in the US and Europe, and can still be potently neutralized by Nbs 20 and 21^25^.

### Class II Nbs bind non-RBS epitopes and block ACE2 binding

**Fig. 3:**
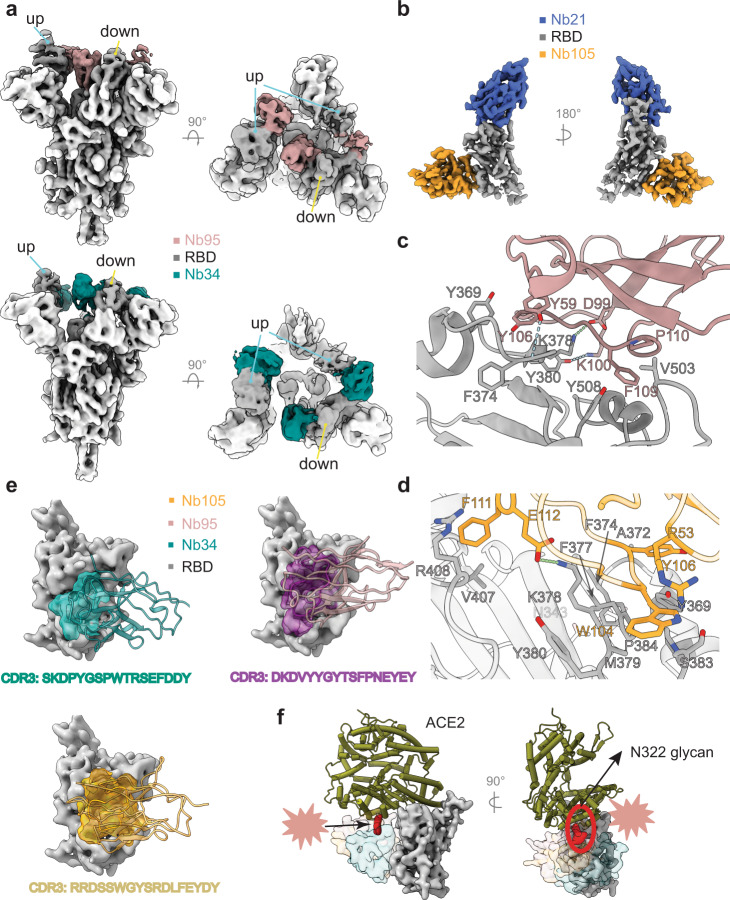
Structures of class II Nbs (95, 34, and 105). **a** Cryo-EM structures of Nbs 95 and 34 in complex with S. **b** Cryo-EM structure of the Nb105:Nb21:RBD complex. **c** Nb95: RBD interactions. Residues in pink denote Nb95 for RBD binding. **d** Nb105: RBD interactions. Residues in yellow denote Nb105 for RBD binding. **e** Class II Nb:RBD interactions are predominantly mediated by CDR3. Nbs are represented as ribbons. The CDR3 loops are shown as surface representations. **f** Steric effects of class II Nbs on hACE2:RBD interactions. N322 glycosylation (ACE2) is presented in red density.

Class II Nbs (95, 34, and 105) can potently neutralize SARS-CoV-2 below 150 ng/ml. Cryo-EM analysis of Nbs 95 and 34 with S revealed two major classes of the complexes with an overall resolution of 3.4 and 3.5 Å, respectively: (1) two-up-one-down RBDs (Fig. 3a, Supplementary Figs. 6a, b and 7a) and 2) three-up RBDs with high flexibility (Supplementary Fig. 6c). Nb105, on the other hand, forms an elongated structure with two copies of S in all RBD-up conformations (Supplementary Fig. 8a, b). The strong preferred orientation of this extended dimeric structure on the EM grids limits a high-resolution reconstruction to accurately define the Nb105:RBD interface. To map the epitope, therefore, we assembled a trimeric complex of Nb21:Nb105:RBD. The resulting ~60 kDa complex was stable and was resolved by cryo-EM at 3.6 Å (Fig. 3b, Supplementary Fig. 8c–f).

Class II Nbs bind RBD primarily through the hydrophobic residues on a relatively long CDR3 loop (17 or more residues) (Fig. 3c–e). Major interactions of Nb95:RBD and Nb105:RBD were resolved by local refinements (Methods). After the assignment of bulky side chains, such as Tyrosine and Phenylalanine (Supplementary Fig. 10c), potential polar interactions can be inferred based on modeling. For Nb95, the side chains of CDR3 residues D99 and K100 form ionic or hydrogen-bonding interactions with the side chains of K378 and Y380 of RBD, respectively (Fig. 3c). The side chain of Y55 of Nb95 also forms a hydrogen bond with the main chain carbonyl of F374 of RBD. In addition to those polar interactions, the CDR3 residues P110 and F109 of Nb95 form hydrophobic interactions with the RBD residues V503 and Y508, and residues Y55 and Y106 of Nb95 cluster with the RBD residue Y369 to form aromatic interactions (Fig. 3c, Supplementary Fig. 10b). Similar to Nb95, Nb105 recognizes RBD with CDR3 W104 and Y106 residing in two hydrophobic patches of M379-P384 and Y369-F377, respectively (Fig. 3d). Another hydrophobic residue F111 is clamped between V407 and R408 (RBD), forming a cation-π stacking interaction. The three patches of hydrophobic interactions surround an electrostatic interaction between E112 and K378 of RBD (Fig. 3d).

**Fig. 4:**
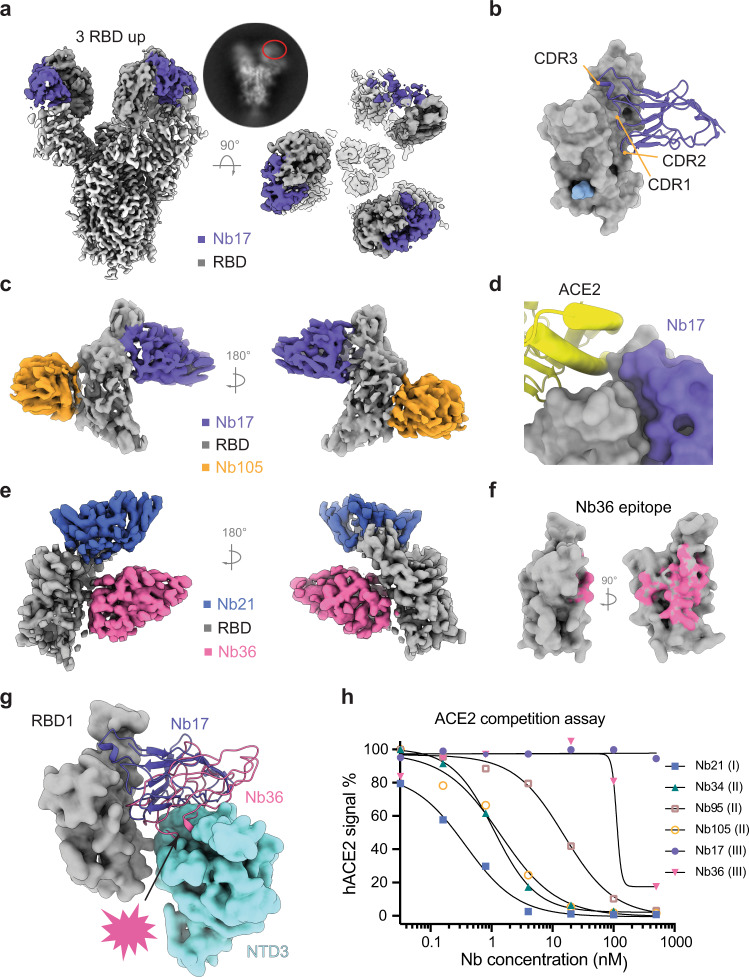
Structures of class III Nbs (17 and 36). **a** Cryo-EM structures of Nb17 in complex with S. **b** Nb17:RBD interactions are mediated by all three CDRs. **c** Cryo-EM structure of the Nb17:Nb105:RBD complex. **d** Nb17 structurally does not overlap with ACE2. **e** Cryo-EM structure of the Nb36:Nb21:RBD complex. **f** Epitope of Nb36 on the RBD surface. **g** Nb17 stacks on NTD via its framework, while isolated Nb36:RBD complex indicates Nb36 would clash with neighboring NTD on S. **h** ACE2 competition assay with the S.

While class II Nbs do not compete with the ACE2 directly, they can still efficiently block ACE2 binding at low nM concentrations consistent with their high neutralization potencies (Fig. 4h). Superposition of ACE2:RBD into the Nb: RBD complexes reveals that binding of Nbs 105 and 95 to the RBD overlaps with the subdomain II of ACE2 (residues 308–326) and the N322 glycan, wherever Nb34 can clash with the glycan (Fig. 3f). Recent crystal structures of camelid Nbs^5^ and synthetic constructs (sybodies)^9^ have revealed a similar mode of binding with substantially lower neutralization potency (over 100-fold) compared to highly affinity matured class II Nbs (95, 34, and 105).

### Class III Nbs utilize distinct neutralizing mechanisms

Nb17 can neutralize the virus in vitro at an half-maximal inhibitory concentration (IC50) of ~25 ng/ml. Notably, all three RBDs on the S trimer were in an open conformation with two having particularly strong densities (Fig. 4a, Supplementary Fig. 9a–d). Nb17 binds a semi-conserved epitope (Supplemental Fig. 13a, b) including a segment spanning residues 345–356 and additional residues that generally do not overlap with the RBS. This epitope is localized on the opposite side of class II Nb epitopes (Fig. 4b). Similar to Nb21, Nb17 also utilizes all three CDRs for RBD recognition. Remarkably, no bulky side chains directly involve interface packing and contribute to the ultrahigh RBD binding affinity. Instead, small hydrophobic residues, such as Ala, Val, and Pro, and polar interactions are the primary contributors at the Nb17:RBD interface (Supplementary Fig. 9g). 3D variability analysis shows that Nb17 density stacks on the adjacent NTD preferring the open conformation of all RBDs when Nb17 is bound (Movie S1).

We also reconstituted the Nb17:Nb105:RBD complex to characterize the interface interactions (Fig. 4c, Supplementary Fig. 9e, f). Superposition of the Nb17:RBD complex to the ACE2:RBD complex indicates that Nb17 would not interfere with ACE2 interactions (Fig. 4d). Structural alignment reveals that the CDR3 of Nb17 overlaps with Nb21 to compete for RBD binding (Supplementary Fig. 9h).

To further explore the mechanism by which Nb17 efficiently neutralizes SARS-CoV-2, we assembled the Nb17:S complex and performed a limited proteolysis experiment using proteinase K to assess the potential impact of all-RBD-up conformation (Methods). Compared to S itself (the super stable hexapro variant) or the S: Nb105 complex which is difficult to digest, Nb17 binding to S appears to increase the proteolysis rate of S in a manner similar to hACE2 binding. Here, we speculate that with ultra-high affinity, Nb17 can lock S1 in a specific, open conformation that promotes the unprogrammed spike post-fusion transition and immature cleavage of S1 (Supplementary Fig. 12a)^26–28^.

Nb17 is resistant to all the dominant natural RBD mutations that we have tested, except for the Kappa variant, presumably due to the L452R mutation. Here, the long side chain of R at position 452 may disrupt the interfacial packing with the adjacent residues of S30, V96, and Q98 on Nb17 (Supplementary Fig. 12b). Compared to the Nb21:RBD interface, where E484 is buried inside the core of the interface, E484 localizes at the rim of the Nb17:RBD interface (Supplementary Fig. 9h). As such, while E484 directly contacts Nb17, the mutations (E484K and E484Q) do not affect RBD binding (Fig. 1a). The loss of binding to the super variant of B62 is likely caused by two other point mutations (I468 and T470) (Supplementary Fig. 13b).

While Nb36:S complex is highly soluble, to our surprise, particles were not detected on the EM grids under cryogenic conditions. Therefore, to characterize Nb36:S interactions, we titrated different concentrations of Nb36 with S protein and imaged the complexes by negative stain EM. The increasing concentration of Nb36 coincided with an enhanced blurring of the particles, which compromised contrast in the electron micrographs (Supplementary Fig. 11a). This observation suggests that Nb36 can destabilize the integrity of the spike. To test this, we employed thermal shift melting assays under similar conditions as those used for negative stain EM. Consistently, an increase in Nb36 concentration correlated with a decrease in protein melting temperature, indicating that Nb36 promotes instability of the S complex (Supplementary Fig. 11b). To map the epitope, we reconstituted and imaged the Nb36:Nb21:RBD complex by cryo-EM (Fig. 4e, Supplementary Fig. 11c–e). The analysis reveals that the Nb36 epitope partially overlaps with Nb17 while exhibiting no overlap with Nb21 (Fig. 4f). The epitope covers a small segment on the non-RBS region (residues 353–360 of RBD) as well as distinct, non-RBS epitope residues that contact Nb17. Nb36 binds RBD in an orientation that is markedly different from Nb17. Superposition of the structure onto S reveals that Nb36 would have a significant steric clash with the neighboring NTD in the trimeric S complex (Fig. 4g). Facilitated by the small size, Nb36 may insert its convex paratope residues between an RBD and the adjacent NTD to destabilize the spike.

To validate this hypothesis, we employed analytical SEC to check the sizes of Nb:RBD complexes. Our analysis reveals that Nb36 binding to S leads to the formation of a smaller complex than the Nb21:S complex and interestingly the S itself (Supplementary Fig. 12c). Dynamic light scattering (DLS) was used to further substantiate the negative stain and SEC results. After two hours of incubation at room temperature, the Nb36:S complex showed a substantially smaller hydrodynamic radius (R_h_) than the Nb21: S complex, which has an identical radius with the transiently formed Nb36: S complex (Supplementary Fig. 12d). Together, these data suggest that Nb36 can efficiently neutralize SARS-CoV-2 by destabilizing the spike. Since a super stable S variant (HexaPro) was used for this study, it is anticipated that Nb36 binding may have a more dramatic impact on the highly flexible wild-type spike^24^. Moreover, this destabilization mechanism is a reminiscence of mAb CR3022. However, Nb36 targets a completely different epitope from CR3022 with substantially higher neutralization potency (~7 nM)^8,29,30^.

### Class III RBD Nbs is a previously unidentified class of neutralizing Nbs

**Fig. 5:**
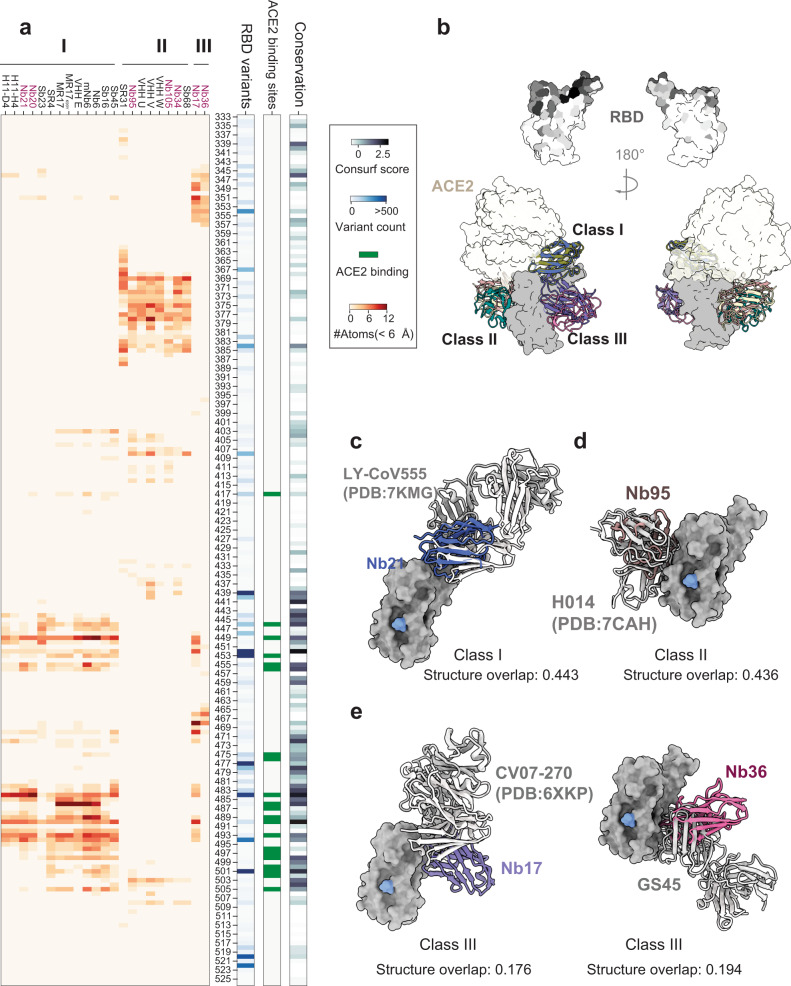
Class III Nbs bind semi-conserved epitopes unique to Nbs. **a** Epitope clustering analysis of RBD Nbs and correlation with RBD sequence conservation and ACE2 binding sites. The conservation scores of SARS-CoV-2 Spike RBD amino acids were computed by ConSurf server using the empirical Bayesian method from the multiple sequence alignment and normalized by the *z*-score method. **b** Overview of three Nb classes binding to the RBD, RBD surface was colored based on conservation (ConSurf score). **c**–**e**. Structural comparison of different classes of Nbs with the closest mAbs for RBD binding.

To investigate the RBD epitopes and Nb neutralization mechanism systematically, we analyzed all the available structures (deposited by March, 2021) that include Nb: RBD interactions (Fig. 5a). Epitope clustering supports the notion of three distinct classes of neutralizing Nbs. As expected, most Nbs bind class I epitopes that mainly cover RBS (Fig. 5a, b). Class I and II epitopes are shared between Nbs and mAb (Fig. 5c, d). In contrast, class III Nbs are unique among all the neutralizing Nbs and mAbs that have been characterized (Fig. 5e). Class III epitopes are in close proximity to the neighboring NTD. Thus, accessing these epitopes is likely elusive for mAbs due to steric hindrance imposed by their large sizes. Here, with optimal orientations and substantially smaller sizes, Nbs can target relatively conserved epitopes (Supplementary Fig. 13) where the virus may have relatively low mutational tolerance^31^.

### Class II and III Nbs target conserved RBD epitopes

To assess the epitope conservation, we selected and aligned 12 RBDs from the major clades of the sarbecovirus family (i.e., lineage B of beta-coronavirus or SARS-like)^32^. Major RBD epitope residues for each class of Nbs, and the epitope sequence identities to RBD_SARS-CoV-2_ were shown in Supplementary Data Fig 13a, b. The median epitope identities for class I, II, and III Nbs are 50% (*σ* = 11.1%), 82.6% (*σ* = 5.7%), and 76.5% (*σ* = 10.3%), respectively.

We also evaluated the binding of potent neutralizing Nbs to RBD_SARS-CoV_, which shares ~73% sequence identity with RBD_SARS-CoV-2_. Consistent with epitope conservation analysis, the ELISA results show that unlike class I and III Nbs, potent neutralizing class II Nbs (specifically, Nb95 and Nb105, but not Nb34) binds strongly to both RBD_SARS-CoV_ and RBD_SARS-CoV-2_ by targeting highly conserved RBD epitopes (Supplementary Fig. 13c, d). It is possible that specific and ultrapotent Class II Nbs may be used for the further bioengineering of pan-sarbecovirus Nb constructs.

### Nbs and mAbs are differently affected by Spike mutations

**Fig. 6:**
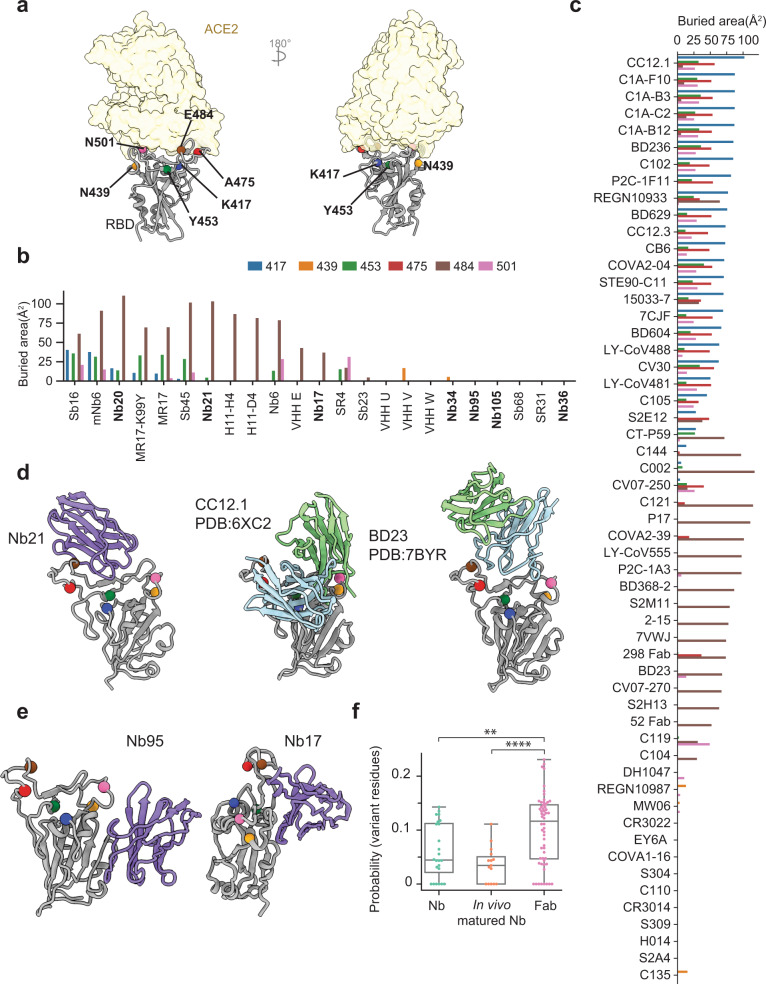
mAbs and Nbs binding to RBD are differently affected by mutations in the circulating variants. **a** Localization of six RBD residues where major circulating variants mutate. **b** Buried surface area of Nbs by different RBD residues. **c** Buried surface area of Fabs by different RBD residues. **d**, **e** Representative structures of different classes of Nbs with major variant residues shown as spheres. Two Fab structures that bind similarly to Class I Nbs were shown on the side. **f** The boxplot showing the probability of epitope residues coinciding with the variant mutations (*n* = 24, 15, and 56 for Nbs, in vivo matured Nbs, and Fabs, respectively). Box plots indicate median (middle line), 25th, 75th percentile (box), and 5th and 95th percentile (whiskers) as well as outliers (single points). Statistical analysis was performed using a two-tailed student *t* test, *p < 0.05, **p < 0.01, ***p < 0.001, ****p < 0.0001.

To understand how the unique binding modes translate to high resistance against SARS-CoV-2 mutants, we compared three Nb classes with mAbs. Buried surface area (BSA) of RBD interfacing residues from both Nb and mAb-bound structures were calculated and compared systematically (Fig. 6a–c). The analysis reveals that the majority of mAbs (83%) use at least one of the mutated RBD residues to bind, with 60% using two or more variant residues for RBD interactions. In contrast, Nbs target these sites substantially less frequently (Fig. 6b) with the exception of class I Nbs, which predominantly recognizes the hot spot fostered by E484 (Fig. 6d). Other classes do not bind these variant residues directly (Fig. 6b, e).

The fact that many critical mutations localize on the RBS is intriguing (Fig. 5a). Under selection pressures, the virus appears to have evolved an efficient strategy to evade host immunity by preferentially targeting this critical functional region. Specific RBS mutations (such as K417N and E484K/Q) may help optimize host adaptation (improved ACE2 binding) to achieve higher transmissibility^33^. In parallel, as RBS is the main target of serologic response, the mutations provide an effective means for the resulting variants to escape the neutralizing pressure from serum polyclonal antibodies^23,31,33,34^. Since most clinical mAbs originate from convalescent plasma, it is not surprising that they are less effective against convergent circulating variants^15,16^. Fundamentally, this is different from neutralizing Nbs which have not been co-evolving with the virus and therefore are likely less sensitive to the plasma-escaping variants. Indeed, we found that the probability of neutralizing Nb epitopes coinciding with the variant mutations was substantially lower than that of mAbs (Fig. 6f). This is particularly the case for potent and in vivo affinity-matured Nbs. Together with the functional data (Fig. 1), our systematic structural analysis provides a framework to understand how potent neutralizing Nbs can resist the convergent variants. It is conceivable that Nbs may provide additional therapeutic benefits over mAbs for the evolving variants of SARS-CoV-2.

### Systematic comparisons of mAbs and Nbs for RBD binding

**Fig. 7:**
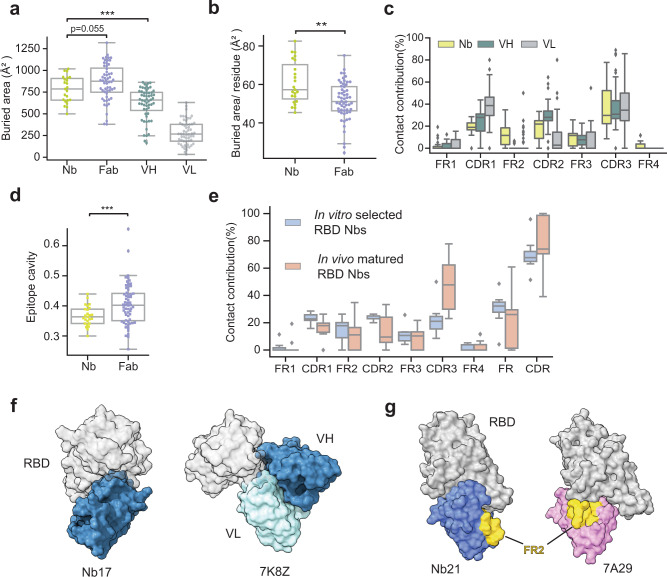
Comparisons of RBD neutralizing Nbs and mAbs. **a** Buried surface areas of RBD: Nb and RBD: Fab complexes. VH heavy chain, VL light chain. **b** Buried surface areas per-interface residue for Nbs and Fabs. **c** The contact contribution of CDRs and FRs of Nbs and Fabs in RBD binding (using a 6 Å cutoff). Contact contribution % was calculated as # of contacting residues on CDR or FR region/total # of contacting residues. **d** Quantification of interface cavity. *Y*-axis is the curvature value. (*n* = 25 and 60 for Nbs and Fabs, respectively). Box plots indicate median (middle line), 25th, 75th percentile (box), and 5th and 95th percentile (whiskers) as well as outliers (single points). Statistical analysis was performed using a two-tailed student *t* test, *p < 0.05, **p < 0.01, ***p < 0.001, ****p < 0.0001. **e** Comparison of contributions from CDRs and FRs for RBD binding between in vivo matured Nbs and in vitro selected Nbs (*n* = 9 and 15 fpr in vitro selected Nbs and in vivo matured Nbs, respectively). **f** Representative structures of 7d showing different binding modes (epitope curvature) of an Nb and a Fab. Nbs target concave RBD surfaces to achieve high-affinity binding. **g** Representative structures of 7e showing the direct involvement of FR2 from an in vitro selected Nb (PDB# 7A29) for RBD interaction.

We compiled and analyzed all the available structures including 56 distinct mAb-bound complexes and 23 Nb-bound complexes (Supplementary Table 4). Nbs have lower BSA values than Fabs (*μ*_Nb_ = 779 Å^2^ vs. *μ*_Fab_ = 862 Å^2^, *p* = 0.055) (Fig. 7a). In addition, the distribution of BSA for Nbs is distinct from Fabs and is substantially narrower (*σ*_Nb_ = 151 Å^2^, *σ*_Fab_ = 210 Å^2^)^35^ (Fig. 7a). Despite the smaller size, Nbs have evolved multiple strategies for high-affinity RBD binding. They exploit surface residues (especially using CDR3 loops) significantly more efficiently than Fabs for RBD engagement (Fig. 7b, c). Nbs also have higher BSA per-interface residue (Fig. 7b). The involvement of the framework (FR) regions in RBD binding, particularly FR2, is also evident probably due to the absence of light chain pairing (Fig. 7c). Interestingly, we found that compared to in vivo affinity-matured RBD Nbs, in vitro selected Nbs to tend to use highly conserved FR sequences more extensively for interactions which may lead to decreased specificity (Figs. 7e, g). Compared to Fabs, Nbs bind significantly more concave surfaces (Methods) to tighten the interactions (Fig. 7d, f). Finally, neutralizing Nbs employ electrostatic interactions more extensively while both types of antibodies predominantly use hydrophobic interactions to achieve high specificity (Supplementary Figs. 14 and 15).

## Discussion

The prospect of curbing the pandemic relies on the development of effective vaccines and therapeutics that resist both the current and future circulating variants of SARS-CoV-2. Highly selective neutralizing Nbs and the multivalent Nb forms represent some of the most potent antiviral agents that have been developed to date^6–8,36^. Critically, a recent study has demonstrated the preclinical efficacy and efficiency of using an ultrapotent aerosolizable Nb for inhalation therapy of SARS-CoV-2 infections in a sensitive COVID-19 model^14^. Here, our structure analysis reveals that neutralizing Nbs can be grouped into three epitope classes. Class I contains some of the most potent SARS-CoV-2 neutralizing Nbs that have been identified to date. Ultrapotent class I Nbs such as Nbs 20 and 21 can neutralize SARS-CoV-2 (Munich strain) with IC50s of 66 and 22 pM, respectively^37^. They target variable RBS and the ultrahigh binding affinities are not affected by the highly transmissible Alpha variant (B.1.1.7). However, the RBD binding of class I Nbs may be abolished by a single point mutation (E484K/Q) that is present in Gamma (P.1), Beta (B.1.351), or Kappa (B.1.617.1) variants. Class II Nbs strongly target conserved epitopes that are resistant to the current VOCs and likely the mutational escape. Class III Nbs bind with high-affinity previously unidentified epitopes including Nb17 epitope that is likely inaccessible to large conventional antibodies. They can efficiently neutralize SARS-CoV-2 at or below 100 ng/ml.

Both classes I and II Nbs potently neutralize SARS-CoV-2 by sterically interfering with ACE2 binding. Interestingly, we found that class III Nbs can employ distinct neutralization mechanisms that do not rely on ACE2 competition. Specifically, Nb17 can lock the spike in an all-RBD-up conformation, which may lead to immature cleavage of S1 and loss of function of a spike by the cellular protease activity. Nb36, on the other hand, may destabilize the spike to efficiently neutralize the virus. Further studies would be needed to fully understand these mechanisms during viral infection in vivo. Moreover, while the preclinical efficacy of the trimeric Nb21 construct (PiN-21) has been demonstrated^14^, it would be highly interesting to evaluate the in vivo efficacy of potent class II and III Nbs which target more conserved epitopes, the multi-epitope/multivalent forms and their combinations for neutralizing the VOCs of SARS-CoV-2, especially the Gamma (P.1), Beta (B.1.351), Kappa (B.1.617.1), and the prevalent Delta (B.1.617.2) strains that have shown to evade the vaccinated sera and clinical mAbs.

In summary, our structure–function investigations provide a framework to map neutralizing epitopes systematically and to understand the structural basis and mechanisms by which Nbs efficiently and uniquely target the spike to inhibit the virus and its variants. The structural findings presented here may also help the rational design of pan-sarbecovirus and pan-coronavirus therapies and vaccines.

## Methods

### Protein expression and purification

The plasmid with cDNA encoding SARS-Cov-2 spike HexaPro (S)^24^ was obtained from Addgene. To express the S protein, HEK293-ES cells were transiently transfected with the plasmid using polyethyleneimine and 3.5 mM valproic acid sodium salt to enhance protein production. After 3 h of transfection, 1 μM kifunensine was added to further boost protein expression. Cell culture was harvested three days after transfection and the supernatant was collected by high-speed centrifugation at 20,000×*g* for 30 min. The secreted S protein in the supernatant was purified using Ni-NTA agarose columns. Protein eluates were then concentrated and further purified by size-exclusion chromatography using a Superose 6 10/300 column (Cytiva) in a buffer composed of 20 mM Hepes pH 7.5 and 200 mM NaCl. The purified S protein was then pooled and concentrated to 1 mg/ml.

The RBD of SARS-CoV-2 was expressed and purified from insect cells using affinity chromatography^8^. Briefly, RBD was expressed in Sf9 insect cells as a secreted protein using the baculovirus method. A FLAG-tag and an 8× His-tag were fused to the N terminus of the RBD sequence, and a TEV protease cleavage site was inserted between the His-tag and RBD. The protein was purified by nickel-affinity resins, followed by overnight TEV protease treatment and size exclusion chromatography (Superdex 75). The purified protein was concentrated in a buffer containing 20 mM Hepes pH 7.5 and 150 mM NaCl. RBD mutants (with His-tag) were purchased through Sino Biologics or Acro Biosystems.

Nanobody genes were codon-optimized and synthesized by Synbio^8^. All nanobody sequences were cloned into pET-21b(+) vectors using EcoRI and HindIII restriction sites. Plasmids were transformed into BL21 (DE3) cells and plated onto Agar gel media with 50 μg/ml ampicillin. Agar plates were incubated at 37 °C overnight, and single colonies were picked for protein purification. The cell culture was allowed to grow at 37 °C to an OD600 of 0.5–0.6, at which point the temperature was lowered to 16 °C and 0.5–1 mM IPTG was added to induce protein expression overnight. Cells were then pelleted, resuspended in a lysis buffer (1× phosphate-buffered saline (PBS), 150 mM NaCl, 0.2% Triton-X100, and protease inhibitors), and ultrasonicated on ice. The clarified cell lysate was collected by centrifugation at 15,000×*g* for 10 min. His-tagged Nbs were captured using cobalt resin and eluted with a pre-chilled buffer containing imidazole (50 mM NaPO4, 300 mM NaCl, 150 mM Imidazole, pH 7.4). Nbs eluted from His-Cobalt resin were further purified using a Superdex 75 gel filtration column using filtered 1× PBS. Nbs were used fresh or flash frozen and stored at −80 °C before use.

### Negative-stain electron microscopy

For negative staining electron microscopy, 3 µl of specified concentration of Nb36 with the S protein was applied to a glow-discharged grid coated with carbon film. The sample was left on the carbon film for 60 s, followed by negative staining with 2% uranyl formate. Electron microscopy micrographs were recorded on a Gatan Ultrascan CCD camera at 22,000× magnification in an FEI Tecnai 12 electron microscope operated at 100 keV.

### Cryo-EM sample preparation and imaging

For S complexed with Nb21, Nb34, and Nb95, each Nb was mixed with the S protein at a molecular ratio of 5 to 1 and incubated at 4 degrees for 30 min. The complex was then diluted in a buffer containing 20 mM Hepes pH 7.5 and 200 mM NaCl to reach a concentration of the S protein at 0.2 mg/ml. Then the sample was applied to a 1.2/1.3 UltrAuFoil grid (Electron Microscopy Sciences) that had been freshly glow-discharged and plunge-frozen in liquid ethane using an FEI Vitrobot Mark IV. All cryo-EM data were collected on Titan Krios transmission electron microscopes (Thermo Fisher) operating at 300 kV. For the S and Nb21 complex, images were acquired on a Falcon 3 detector, with a nominal magnification of 96,000, corresponding to a final pixel size of 0.83 Å/pixel. For each image stack, a total dose of about 62 electrons was equally fractionated into 70 fractions with ~0.88e^−^/Å^2^/fraction. EPU 2 Software was used to automate data collection. Defocus values used to collect the dataset ranged from −0.5 to −3.5 μm.

For the complexes of S with Nb95 and Nb34, sample preparation and data collection were similar to those for the complex of S with Nb21 except that the data were acquired on a Gatan K3-Summit detector. Further details of data collection parameters are summarized in Supplementary Tables 2 and 3.

For S complexes with Nb17 and 105, purified Nbs were mixed with the SARS-CoV-2 S HexaPro trimer with a 2:1 molar ratio Nbs to a final concentration of 0.1 mg/mL S protein and incubated at room temperature for two hours. Cryo-EM grids (Quantifoil AU 1.2/1.3 300 mesh) were glow-discharged and coated with graphene oxide thin layer flakes following the protocol from ref. ^38^ (figshare. Media. 10.6084/m9.figshare.3178669.v1). The cryo-EM specimens were prepared using an FEI Vitrobot Mark IV with 3.5 μl of Nb:S complex. Grids were blotted for 3 s with blot force −5 in 100% humidity at 4 °C prior to plunge freezing. The frozen-dehydrated grids were transferred to a Titan Krios (Thermo Fisher Scientific) transmission electron microscope equipped with a Gatan K3 direct-electron counting camera and BioQuantum energy filter for data acquisition. Movies of the specimen were recorded with a nominal defocus setting in the range of −0.5 to −2.0 μm using SerialEM with beam-tilt image-shift data collection strategy with a 3 × 3 patterns and 1 shot per hole. The movie stacks were collected in the correlated double sampling (CDS) super-resolution mode of the K3 camera at a nominal magnification of 81,000 yielding a physical pixel size of 1.08 Å/pixel. Each stack was exposed for 5 s, with each frame exposed for 0.1 s, resulting in a 50-frame movie. For datasets without using CDS mode, the movie stacks were collected in the super-resolution mode at a nominal magnification of 81,000 with an exposure time of 2.5 s, and each frame exposed for 0.05 s. The total accumulated dose on the specimen was 40 e/Å^2^ for each stack.

For the trimeric Nb complexes (Nb105:RBD: Nb21, Nb17:RBD: Nb105, and Nb36:RBD: Nb21), two purified Nbs were mixed with purified RBD with 1.1:1.1:1 molar ratio and subsequently polished by size-exclusion chromatography. Peak fraction corresponding to the trimeric complexes was used for cryo-grid preparation. Movies of the specimen were recorded with a nominal defocus setting in the range of −0.5 to −2.5 μm using SerialEM or Latitude S with beam-tilt image-shift data collection strategy with a 3 × 3 patterns and 3 shot per hole. The movie stacks were collected in the CDS super-resolution mode of the K3 camera at a nominal magnification of 165,000 yielding a physical pixel size of 0.52 Å/pixel. Each stack was exposed for 2.8 s, with each frame exposed for 0.1 s, resulting in a 28-frame movie. The total accumulated dose on the specimen was 108 e/Å^2^ for each stack.

### Cryo-EM data processing

For the samples of S protein with Nb21, Nb34, and Nb95, cryo-EM data processing was performed using Relion 3.1. Beam-induced motion correction was performed using the motion correction program implemented in Relion to generate average micrographs and dose-weighted micrographs from all frames. Contrast transfer function (CTF) parameters were estimated using CTFFIND 4.1.12 from average micrographs. The loG-based auto-picking procedure was used for reference-free particle picking. Initial particle stacks were subjected to 2D classification and the best class averages that represented different views were selected as templates for second round automatic particle picking from the dose-weighted micrographs.

For the S protein with Nb21 data, approximately 900,000 particles were auto-picked from 2574 micrographs for further processing. The whole set of particles was cleaned to remove contaminants or junk particles by 2D classification and 3D classification using 2× binned particles. Finally, approximately 135,000 particles were used for 3D auto-refinement with the structure of EMD-22221 (EMBD ID) low-pass filtered to 40 Å as the reference. This yielded a map of ~3.4 Å resolution (corrected gold-standard FSC 0.143 criterion). The particles were re-extracted and used for further 3D classification into four classes. The most populated two classes, which contained 53% (1-up-down RBDs) and 29% (2-up-1-down RBDs) of particles were subjected to further 3D auto-refinement. To improve the local density of Nb21 and RBD, focused refinement was performed with a soft mask applied to one down RBD and Nb21, resulting in an improved local resolution ranging from 4.5 to 3.3 Å for the RBD and Nb21 interface. All maps were sharpened using the post-processing program in Relion or DeepEMhancer. The local resolution was estimated by ResMap v1.1.5 in Relion. Similar approaches were used to solve the structures of S protein with Nb95 and Nb34 as well. The detailed information of data processing is shown in Supplementary Figs. 4 and 6–10 and Supplementary Tables 2 and 3.

For other structures, each movie stack was processed on the fly using CryoSPARC live (version 3.0.0)^39,40^. The movie stacks were aligned using patch motion correction with an F-crop factor of 0.5. The CTF parameters of each particle were estimated using patch CTF. Particles were auto-picked using a 220 and 100 Å gaussian blob for Nb:S and 2Nbs: RBD complexes respectively. The numbers of bin2 particles selected after 2D classification are included in Supplementary Table 2. The initial 3D volume and decoys were generated using ab initio reconstruction with a minibatch size of 1000 using a set of rebalanced 2D classes. The particles after 2D clean-up were submitted to one round of heterogeneous refinement with ab initio 3D volume from good 2D classes and decoy 3D volumes from bad 2D classes. Based on the coordinates and angular information of these particles, bin1 particles of the 3D class with well-resolved secondary structure features were re-extracted from the dose-weighted micrographs. For small trimeric complexes, a pixel size that can achieve the resolution limit of the sample, instead of bin1 pixel, was used for the final reconstruction to prevent overfitting. The final particle set was subjected to non-uniform 3D refinements^40^, followed by local 3D refinements, yielding final maps with reported global resolutions using the 0.143 criteria of the gold-standard Fourier shell correlation (Supplementary Table 2). The half maps were used to determine the local resolution of each map and focused classification was performed using Relion 3.0^41,42^. For Nb17:S complex, the final particles (45,362) were aligned to the C3 symmetry axis to expand the particle set to 136,086 (Supplementary Fig. 10c). Then a mask focused on the arc shape including RBD, Nb17, and NTD was created with the binary map extended 10 pixels and a soft edge of 10 pixels. The cryosparc particle set was converted to a relion star file using pyem (https://github.com/asarnow/pyem), and focused classification was performed by Relion with *k* = 3. The class with densities for all three targeted domains well resolved was selected for further local refinement in CryoSPARC 3.0.0.

### Model building, structure refinement, and analysis

For modeling whole S protein with Nbs, the RBD models were generated by docking the atomic model of SARS-Cov2 RBD (PDB ID 7JVB, chain B) into the refined cryo-EM density using Chimera 1.14 (UCSF). Nb structures were modeled ab initio in Coot using based on the locally refined cryo-EM maps and refined in Phenix. After refinement, each residue of the sequence-updated models was manually checked and refined iteratively in Coot version 0.9 and Phenix version dev-3951. Structural models were validated by MolProbity 4.5.1. The final refinement statistics are listed in Supplementary Tables 2 and 3.

### CDR3 loop modeling

To optimize the CDR3 loop conformation, it was modeled ab initio using “RosettaAntibody3” H3 loop modeling with restraints (distance, dihedral, and planar angles^43^) generated by NanoNet. NanoNet is a deep residual neural network, similar to DeepH3^44^, trained on solved CDR3 loops of antibodies and Nbs from the PDB. NanoNet uniqueness comes from the fact that it takes as input only the sequence of the CDRs (each in a single one-hot encoding matrix) without the framework region. In addition, it uses MSE loss and predicts the pairwise distances and angles directly (for angles, it predicts the sine and cosine values to overcome cyclic loss), instead of using categorical cross-entropy loss and trying to predict the pairwise probability distributions. NanoNet architecture consists of two 2D residual blocks, followed by two convolutional layers for each output, with the tan*h* activation function for angles and ReLU (rectified linear activation function) for distances.

For each nanobody, 100 models were generated, and the one that fitted best in the cryo-EM density map was chosen manually.

For Nb20, Nb21, Nb95, Nb105, and Nb34 the models generated were similar to the ones without the optimization. For Nbs 17 and 36, the models generated from “RosettaAntibody3” with NanoNet fitted better in the cryo-EM map and were further refined in the density map.

### Contact heat map

An RBD residue and an Ab/Nb residue were defined in contact if the distance between any pair of their atoms was lower than a threshold of 6 Å. The Ab/Nb contact value of each RBD residue is calculated as the average of all the Ab/Nb contacts. Nb classes were clustered using k-means (*k* = 3).

### Conservation score of SARS-CoV-2 RBD

The conservation scores were obtained from the Consurf server by querying the RBD sequence^45^. The multiple sequence alignment of different RBDs was constructed and the evolutionary rate was calculated using an empirical Bayesian method. The evolutionary rate was then normalized by the *z*-score method to calculate the conservation score, where higher scores indicate more conservation, and lower scores indicate more variability.

### Measurement of buried surface area

The solvent-accessible surface area (SASA) of molecules was calculated by FreeSASA^46^. The buried surface area in the case of the Nb–RBD complex was then calculated using Eq. (1)

1 $${{{{{\rm{BSA}}}}}}=\frac{1}{2}\times [{{{{{\rm{SASA}}}}}}({{{{{\rm{Nb}}}}}})+{{{{{\rm{SASA}}}}}}({{{{{\rm{RBD}}}}}})-{{{{{\rm{SASA}}}}}}({{{{{\rm{complex}}}}}})]$$

### Measuring structural overlap between Nb and best-matched Fab

The best matched Fab for an Nb was obtained using the epitope similarity (Jaccard-index). The Nb–RBD complex structure was superimposed on its best matched Fab–RBD structure and protein volume were calculated using ProteinVolume^47^. Then the structural overlap was calculated using Eq. (2)

2 $${{{{{\rm{Structural}}}}}}\,{{{{{\rm{overlap}}}}}}=[{{{{{\rm{Volume}}}}}}({{{{{\rm{Nb}}}}}})+{{{{{\rm{Volume}}}}}}({{{{{\rm{RBD}}}}}})-{{{{{\rm{Volume}}}}}}({{{{{\rm{complex}}}}}})]/{{{{{\rm{Volume}}}}}}({{{{{\rm{RBD}}}}}})$$

### Measurement of the interface curvature

The interface curvature was calculated as the average of the shape function of the interface atoms of the antigen or the Nb. For this purpose, a sphere of radius *R* (6 Å) is placed at a surface point of the interface atom. The fraction of the sphere inside the solvent-excluded volume of the protein is the shape function at the corresponding atom^48^.

### Enzyme-linked immunosorbent assay (ELISA)

Proteins (SARS-CoV-2 RBD and RBD variants, SARS-CoV RBD) were coated onto 96-well ELISA plates, with 150 ng of protein per well in the coating buffer (15 mM sodium carbonate, 35 mM sodium bicarbonate, pH 9.6) at 4 °C for overnight. The plates were decanted, washed with a buffer (1× PBS, 0.05% Tween 20), and blocked for 2 h at room temperature (1× PBS, 0.05% Tween 20, 5% milk powder). Nbs were serially 5× diluted in blocking buffers starting from 10, 2.5, or 0.5 μM with at least 8 different concentrations and incubated with the well for 2 h at room temperature. After three washes, the anti-T7 tag HRP-conjugated secondary antibodies (Thermo Fisher, Cat# PA1-31449) were diluted at 1:5000 and incubated at room temperature for 1 h. Upon washing, samples were further incubated in the dark for 10 min with freshly prepared 3,3′,5′,5′-tetramethylbenzidine (TMB) substrate. Upon quenching the reaction with a STOP solution, the plates were measured at wavelengths of 450 nm with background subtraction at 550 nm. The raw data was processed and fitted into the 4PL curve using the Prism Graphpad 9.0. IC50s were calculated and fold changes of binding affinity were calculated to generate the heatmap.

For ACE2 competitive ELISA, the super stable spike was coated at 80 ng/ml on the plate. Nbs were serially 5× diluted in blocking buffers from 500 nM to 32 pM with an addition of 60 ng/well of biotinylated hACE2 for competition. No Nb was used as a negative control. Pierce High Sensitivity Neutravidin-HRP antibodies (Thermo Fisher, Cat# 31030) were used at 1:8000. The hACE2 percentage was calculated by the reading at each Nb concentration divided by the reading at the negative control. Then the data was processed and fitted into the 4PL curve using the Prism Graphpad 9.0.

### Molecular dynamics (MD) simulation setup

Input files for MD simulations of SARS-CoV-2 RBD and nanobody complexes were prepared using CHARMM-GUI^49^. MD simulations were performed using the NAMD^50^ and the amber ff19sb^51^, GLYCAM_06j^52^, ions^53^ with the TIP3P water model^54^. Proteins were solvated in a cubic water box with a 16 Å padding in all directions. Sodium ions and chloride ions were added to achieve a physiological salt condition of 150 mM. The systems were energy minimized for 10,000 steps to remove bad contacts. Then, the systems were equilibrated with all heavy atoms restrained harmonically and the temperature raised 10 K per 10,000 steps starting from 0 to 300 K using temperature reassignment. After reaching the desired temperature, harmonic restraints were gradually reduced using a scale from 1.0 to 0 with a 0.2 decrements for every 50,000 steps. MD simulations were performed under the NPT ensemble^55,56^. Langevin dynamics was used for constant temperature control, with the value of Langevin coupling coefficient and the Langevin temperature set to 5 ps and 300 K, respectively. The pressure was maintained at 1 atm using the Langevin piston method with a period of 100 fs and decay times of 50 fs. A time step of 2 fs was used for all the simulations by using the SHAKE algorithm^57^ to constrain bonds involving hydrogen atoms.

### Relative energy contribution with MM-PBSA calculations

For each snapshot, every 1 ns of the 200 ns trajectory of SARS-CoV-2 RBD and nanobody complexes, the binding energy of MM/PBSA was calculated using Eqs. (3) and (4)^58,59^

3 $$\varDelta {G}_{{{{{\rm{binding}}}}}}={G}_{{{{{\rm{complex}}}}}}-{G}_{{{{{\rm{RBD}}}}}}-{G}_{{{{{\rm{Nb}}}}}}$$

4 $$=\varDelta {E}_{{{{{\rm{MM}}}}}}+\varDelta {G}_{{{{{\rm{PB}}}}}}+\varDelta {G}_{{{{{\rm{nonpolar}}}}}}-T\varDelta S$$

where is the molecular mechanic (MM) interaction energy calculated in gas-phase between RBD and nanobody, including electrostatic and van der Waals energies; the desolvation free energy consists of polar (and nonpolar) terms; is the change of conformational entropy on nanobody binding, which was not considered here as the binding epitope on the RBD is very stable and the comparison was performed internally. The decomposition of the binding free energy to the relative energy contribution from individual residues was performed using the MMPBSA.py module in AMBER18^60^.

### Pseudovirus neutralization assay

The 293T-hsACE2 stable cell line and the pseudotyped SARS-CoV-2 particles (wild-type and mutants) with luciferase reporters were purchased from the Integral Molecular. The Alpha (UK) pseudotyped virus contains all of the naturally prevalent mutations for that strain. The Beta contains all of the naturally prevalent mutations except del241–243, which is replaced by an L242H substitution for the pseudovirus (Supplementary Fig. 2). The neutralization assay was carried out according to the manufacturers’ protocols in duplicates. In brief, threefold or fivefold serially diluted Nbs were incubated with the pseudotyped SARS-CoV-2-luciferase for 1 h at 37 °C. At least eight concentrations were tested for each Nb. Pseudovirus in culture media without Nbs was used as a negative control. Totally, 100 µl of the mixtures were then incubated with 100 µl 293T-hsACE2 cells at 3×10e5 cells/ml in the 96-well plates. The infection took ~72 h at 37 °C with 5% CO_2_. The luciferase signal was measured using the Renilla-Glo luciferase assay system with the luminometer at 1 ms integration time. The obtained relative luminescence signals from the wells were normalized according to the negative control and the neutralization percentage was calculated at each concentration. The data was then processed by Prism GraphPad 9.0 to fit into a 4PL curve and to calculate the logIC50 (half-maximal inhibitory concentration).

### Spike conformational change analysis by western blot

SARS-CoV-2 super stable hexapro (6 P) spike trimer was incubated either with hACE2 ectodomain (Acro biosystem, 1:10 molar ratio) or an Nb (1:8 molar ratio) overnight at room temperature. Proteins were then digested with proteinase K (PK, 1:50 enzyme to substrate ratio) for 15 min and 60 min at room temperature. PK was inactivated by mixing with a sodium dodecyl sulfate-polyacrylamide gel electrophoresis loading buffer and heating at 98 °C for 10 min. Inactivated samples were run on a 4–12% Bis–Tris gel (Bolt) before being stained with a Sypro Ruby stain or subject to western blot analysis. For western blot, anti-S2 SARS-CoV-2 polyclonal antibodies (Sino biologics, Cat# 40590-T62, 1:2000 dilution) were used as the primary antibody at 4 °C overnight. A horse radish peroxidase-conjugated goat anti-rabbit secondary antibody was used at 1:5000 dilution (Thermo Fisher, Cat# 31460) for 1 h at room temperature. ECL substrate (Bio-rad) was used to develop S2 signals which were visualized by the Bio-rad Imager. The experiments were repeated four times.

### Protein thermal shift assay

Thermal denaturation of S protein in the presence of an increased concentration of Nb36 was monitored by differential scanning fluorimetry using Protein Thermal ShiftTM dye kit^61^. Briefly, the same protein samples used for negative stain EM were diluted to a final assay concentration of 100 nM in PBS with 1 mM DTT and 1:1000 fluorescence dye (TFS 4461146). The final assay volume was 20 μL, with 1, 5, 10, 100, and 600 nM of Nb36 was added to a final concentration of 100 nM S protein. Heat denaturation curves were recorded using a real-time PCR instrument (StepOneTM) applying a temperature gradient of 1 °C/min. Analysis of the data was performed using Excel. Melting temperatures of protein samples were determined by the inflection points of the plots of −*d*(RFU)/*dT*.

### Statistical analysis

A two-sided student *t* test (assuming unequal variance) was performed in the analysis of the buried area and epitope curvature in Fig. 7a–d.

### Reporting summary

Further information on research design is available in the Nature Research Reporting Summary linked to this article.

## Supplementary information

Supplementary Information

Peer Review File

Reporting Summary

## Data Availability

The cryo-EM maps have been deposited in the Electron Microscopy Data Bank under accession code EMD-24255, EMD-24256, EMD-24257, EMD-23802, EMD-24262, EMD-23782, EMD-23790, and EMD-23788. The atomic coordinates for the deposited map have been deposited in the Protein Data Bank under accession code 7N9B, 7N9C, 7N9E, n/a (due to resolution), 7N9T, 7MDW, 7MEJ, 7ME7, respectively. Source data are provided with this paper. Other data are available from the corresponding author upon reasonable request. Source data are provided with this paper.

## Source data

Source Data
