# Supplementary material for: Potent neutralizing nanobodies resist convergent circulating variants of SARS-CoV-2 by targeting diverse and conserved epitopes
Source: Nat Commun. 2021 Aug 3;12:4676. doi: 10.1038/s41467-021-24963-3 (PMC8333356; doi:10.1038/s41467-021-24963-3)
Supplement: Supplementary file 1 — Supplementary Information [file 41467_2021_24963_MOESM1_ESM.pdf]

Supplementary Information

Potent neutralizing nanobodies resist convergent circulating variants of SARS-CoV-2 by targeting diverse and conserved epitopes

Dapeng Sun[1,10], Zhe Sang[2,3,10], Yong Joon Kim[3,4,10], Yufei Xiang[3,10], Tomer Cohen[5], Anna K. Belford[6], Alexis Huet[6], James F. Conway[6], Ji Sun[7], Derek J. Taylor[8,9], Dina Schneidman-Duhovny[5*], Cheng Zhang[1*], Wei Huang[8*], and Yi Shi[2,3,4*]

1. Department of Pharmacology and Chemical Biology, University of Pittsburgh
2. The University of Pittsburgh and Carnegie Mellon University Program for Computational Biology, Pittsburgh, PA, USA
3. Department of Cell Biology, University of Pittsburgh, PA, USA
4. Medical Scientist Training Program, University of Pittsburgh School of Medicine and Carnegie Mellon University, Pittsburgh, PA, USA
5. School of Computer Science and Engineering, Institute of Life Sciences, The Hebrew University of 6. Jerusalem, Israel
6. Department of Structural Biology, University of Pittsburgh
7. Department of Structure Biology, St. Jude Children's Research Hospital, Memphis, TN, USA
8. Department of Pharmacology, Case Western Reserve University, Cleveland, OH, USA
9. Department of Biochemistry, Case Western Reserve University, Cleveland, OH, USA
10. These authors contributed equally to this work: Dapeng Sun, Zhe Sang, Yong Joon Kim, Yufei Xiang

* Corresponding authors:
yi.shi@pitt.edu (Y.S), wxh180@case.edu (W.H), chengzh@pitt.edu (C.Z) or dina.schneidman@mail.huji.ac.il (D.S)

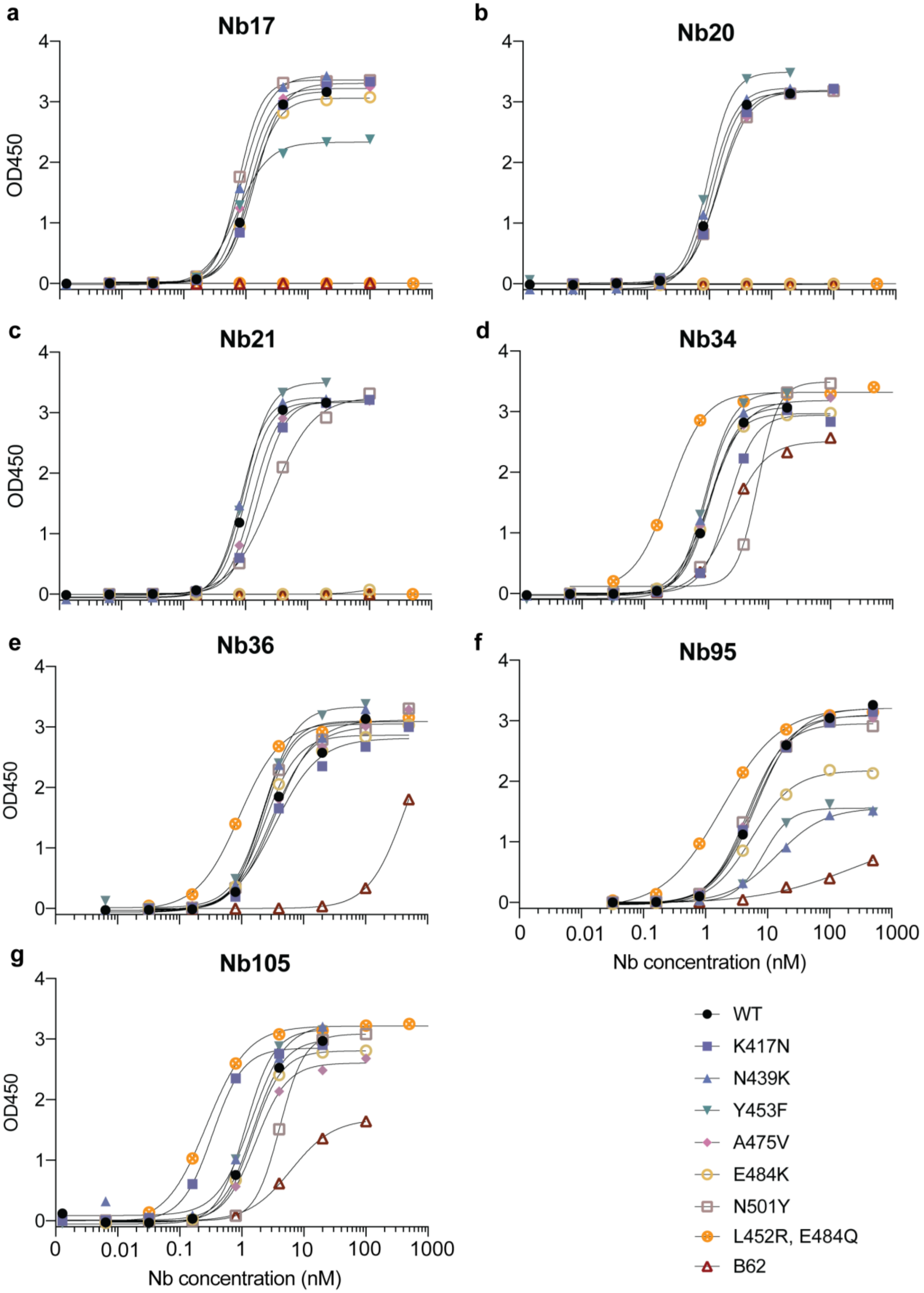

Supplementary Figure 1: ELISA curves of Nbs for RBD mutant binding.
n = 3 biological replicates. Data points are presented as mean values.

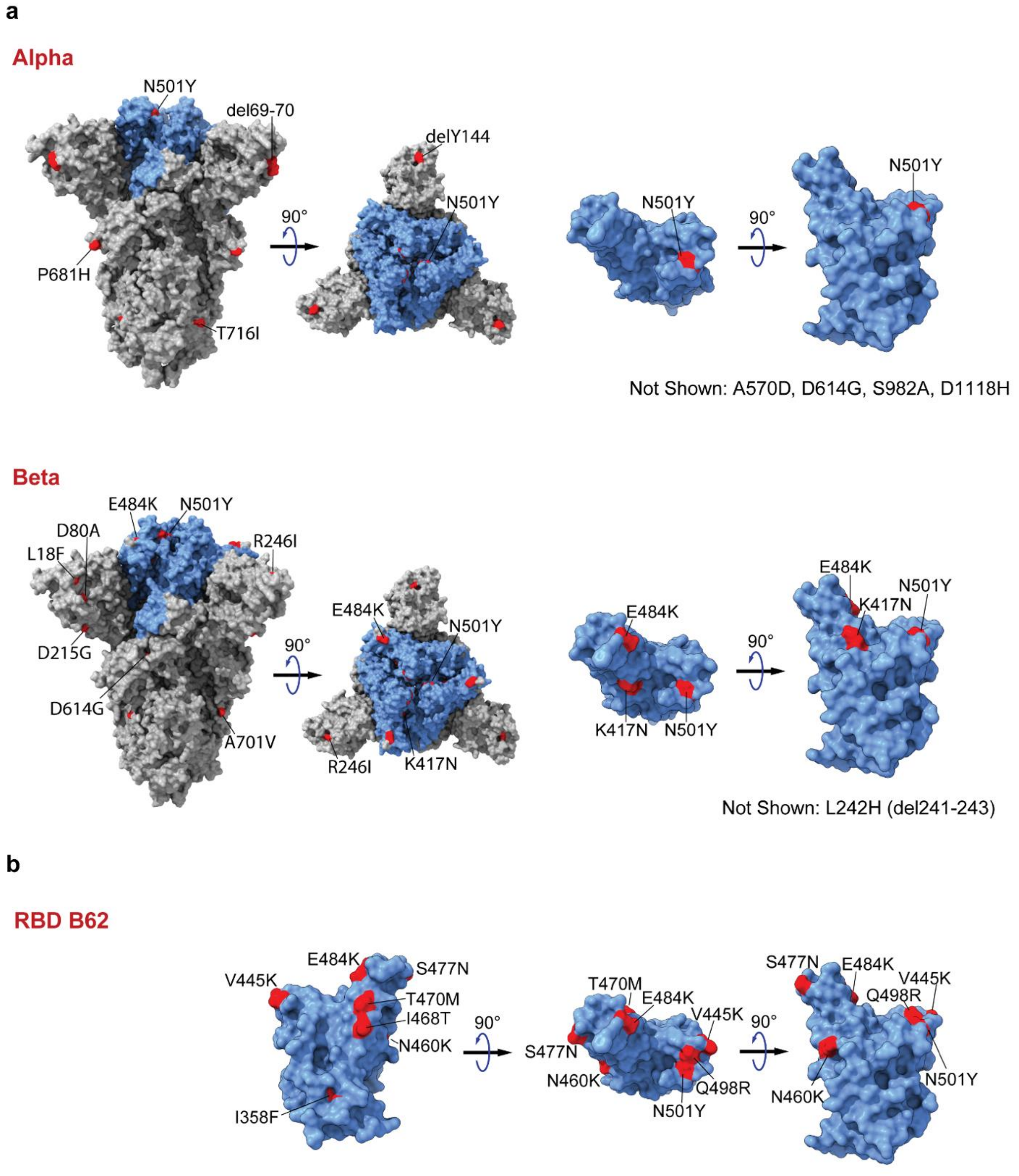

Supplementary Figure 2: Structure representations of SARS-CoV-2 spike trimer glycoprotein and mutations for two prevalent circulating strains.

a. All mutations for VOCs Alpha and Beta highlighted in red. Mutations for Alpha include del69-70, delY144, N501Y, A570D, D614G, P681H, T716I, S982A, and D1118H. Mutations for Beta include L18F, D80A, del241-243, D215G, R246I, K417N, E484K, N501Y, D614G, AND A701V.
b. All mutations for ACE2 affinity matured RBD B62 highlighted in red. Mutations include I358F, V445K, N460K, I468T, T470M, S477N, E484K, Q498R, and N501Y.

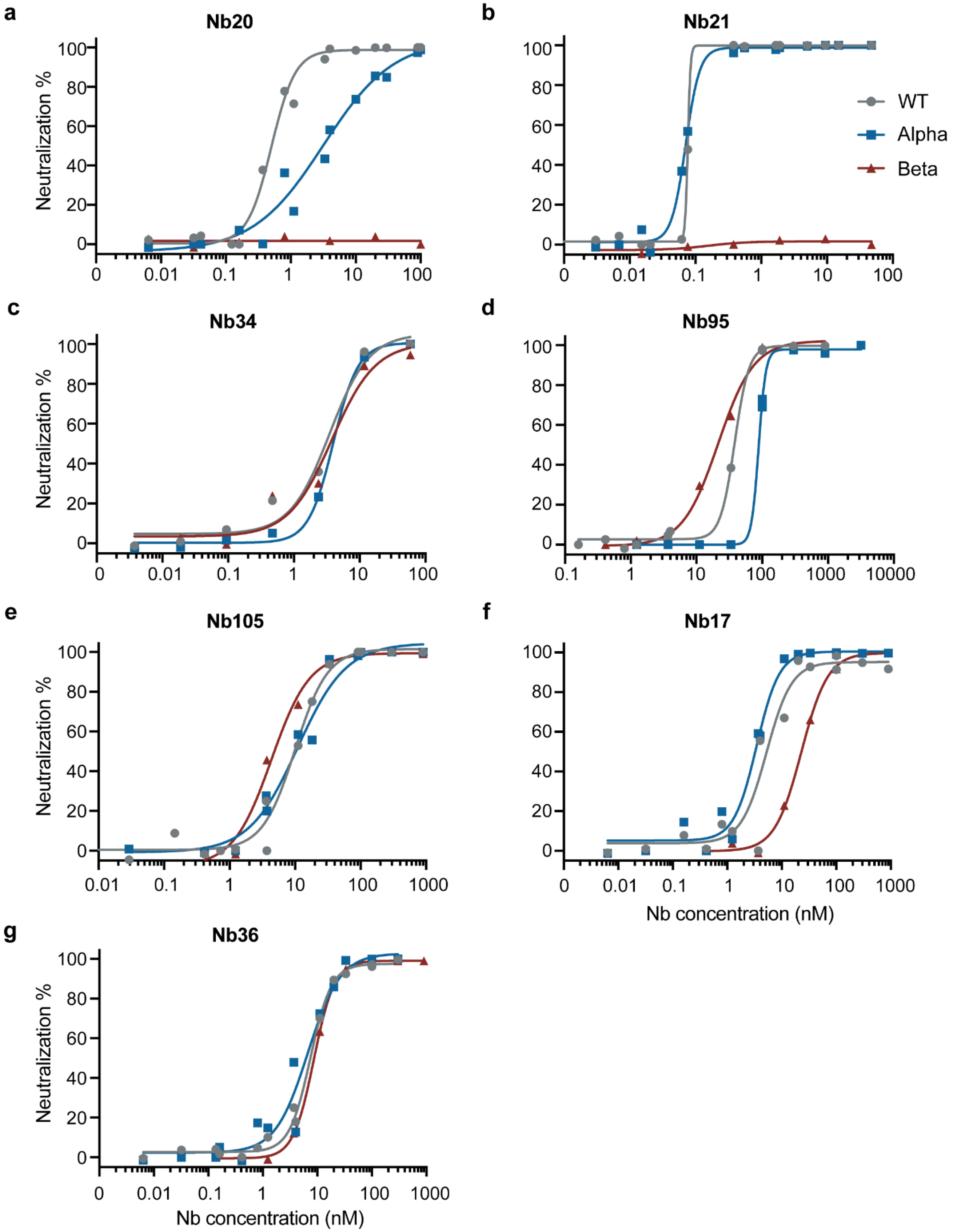

Supplementary Figure 3: Pseudovirus assay results for individual Nbs.
n = 2 biological replicates. Data points are presented as mean values.

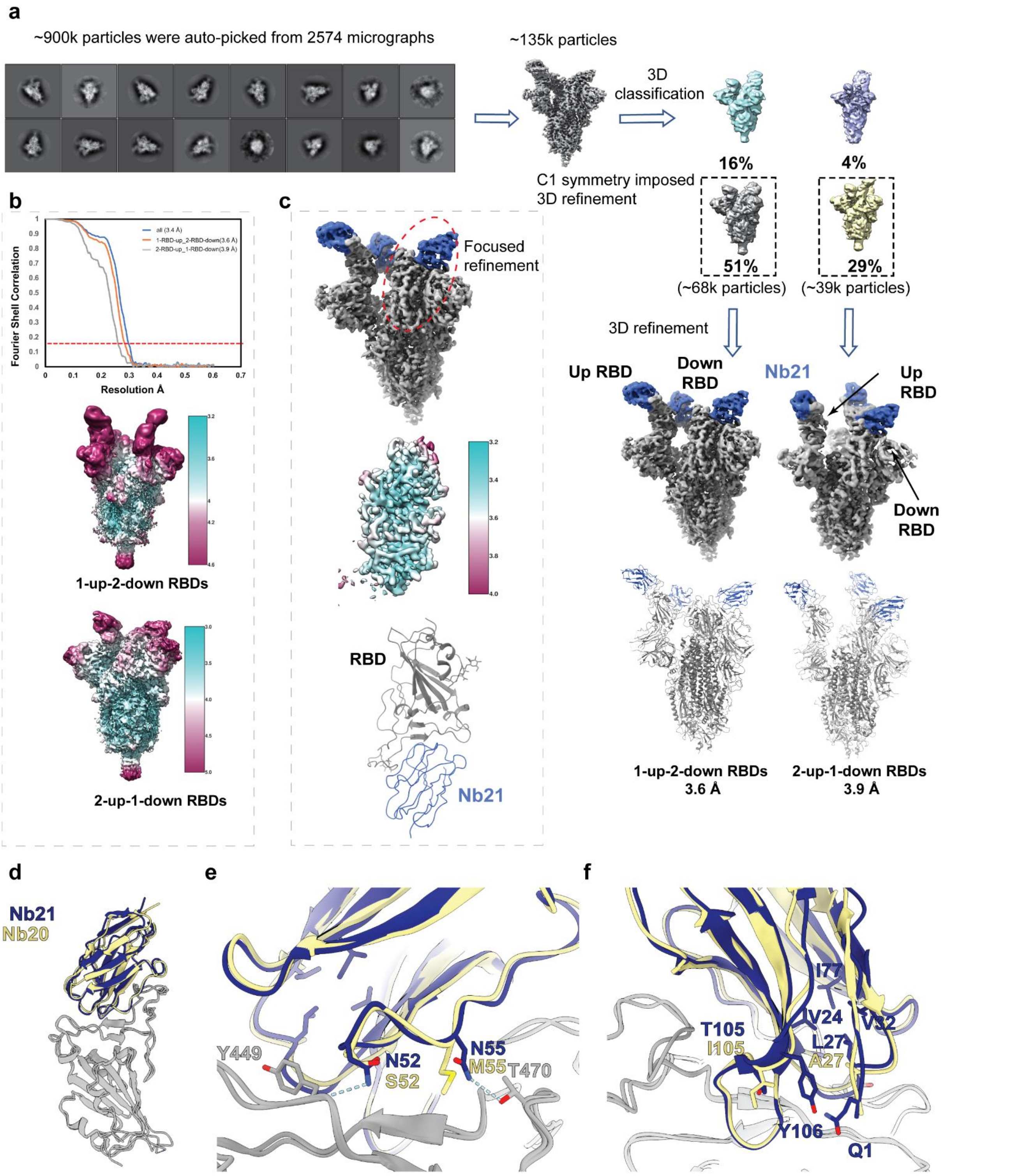

Supplementary Figure 4: Cryo-EM Structure determination of S with Nb21, local refinement of RBD with Nb21 and comparison of Nb21 with Nb20.

a. Cryo-EM Data Processing workflow showing the strategies and particle cohort sizes used to generate the maps discussed in this work. ~900K particles were picked based on the 2D class averages of S with Nb21 for 3D classification. Two major classes with the largest proportions were further refined. One class refined to 3.6 Å corresponds to S with 1-up-2-down RBDs and the other class refined to 3.9 Å corresponds to S with 2-up-1-down RBDs. S is colored in dark gray. Nb21 is colored blue.
b. Fourier Shell Correlation and local resolution estimations for S and NB21 complexes. The red line represents FSC = 0.143.

c. Focused Refinement of one down RBD with Nb21.

d-f. Structural comparison of RBD with Nb21 and with Nb20. Nb21 is colored blue while Nb20 is colored yellow. RBD is colored dark gray and cyan in the structures with Nb21 and Nb20, respectively. Nb21 differs from Nb20 by four residues (all on CDRs). Its RBD binding is very similar to that of Nb20. The two structures can be well aligned with a root mean square deviation (RMSD) of 1.8 Å (all atoms). Here, S52 and M55 on CDR2 in Nb20, are replaced by N52 and N55 in Nb21, which form additional polar interactions with the RBD (e). A27 (CDR1) and I105 (CDR3) of Nb20 are replaced by L27 and T105 in Nb21 (f). While the two residues do not bind RBD directly, the side chain of L27 is buried inside Nb21 to form additional hydrophobic interactions with V24, V32, and I77. The small short side chain of T105 allows the neighboring residue Y106 to point towards the first N-terminal residue Q1 to form a hydrogen bond. This interaction, which is missing in the structure of Nb20:RBD as it is impeded by the presence of the large side chain of I105 in the analogous position of Nb20 I105. These additional interactions may help stabilize CDR1 and CDR3 loops to strengthen Nb21:RBD interactions.

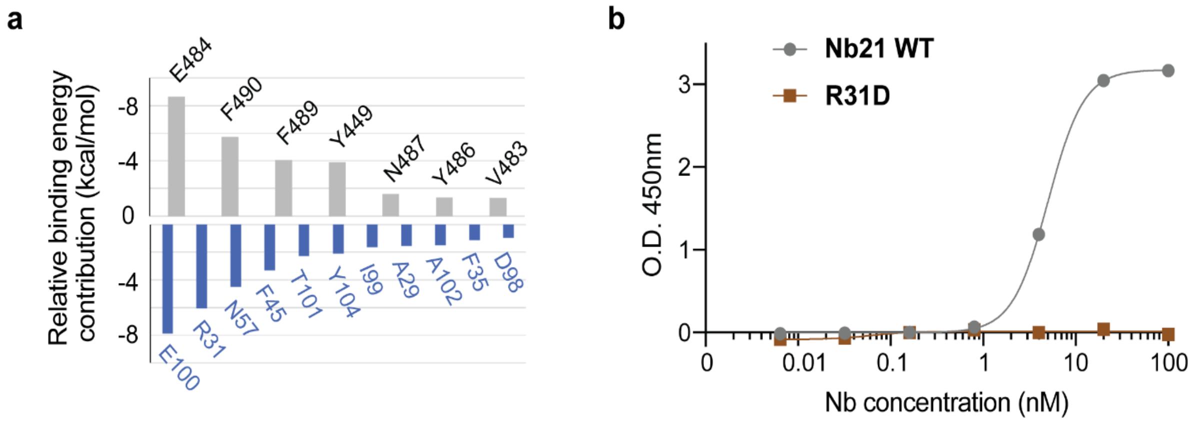

Supplementary Figure 5: Assessment of the RBD:Nb21 interactions using both computational binding energy calculation and experimental mutagenesis.

a. Decomposition of relative binding free energy contribution from individual residues of RBD (top, gray) and Nb21 (bottom, blue) for these more than -1 kcal/mol.

b. ELISA assay showing Nb21 point mutant R31D fails to bind RBD.

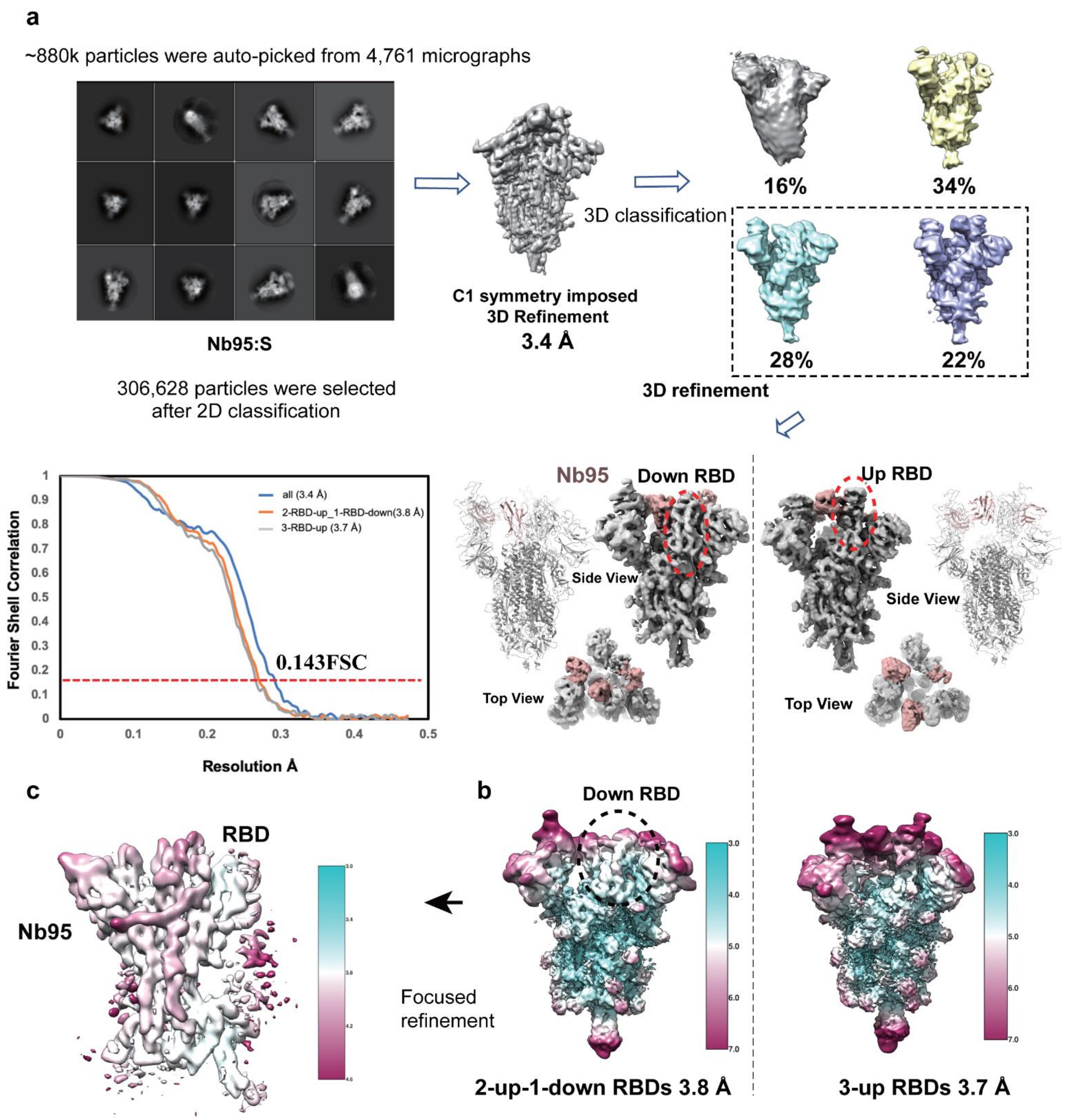

Supplementary Figure 6: Cryo-EM Structure determination of S with Nb95 and focused refinement.

a. ~880K particles were picked based on the 2D class averages of S with Nb95 for 3D classification. Two major classes with the largest proportions were further refined. One class refined to 3.8 Å corresponds to S with 2-up-1-down RBDs and the other class refined to 3.7 Å corresponds to S with 3-up RBDs. S is colored in dark gray. Nb95 is colored in teal. FSC estimations for these two complexes are shown at the lower-left corner. The red line represents FSC=0.143.
b. Local resolution distribution for the two S and Nb95 complexes.
c. Focused Refinement of one down RBD with NB95. The down RBD showed better density compared to up RBDs.

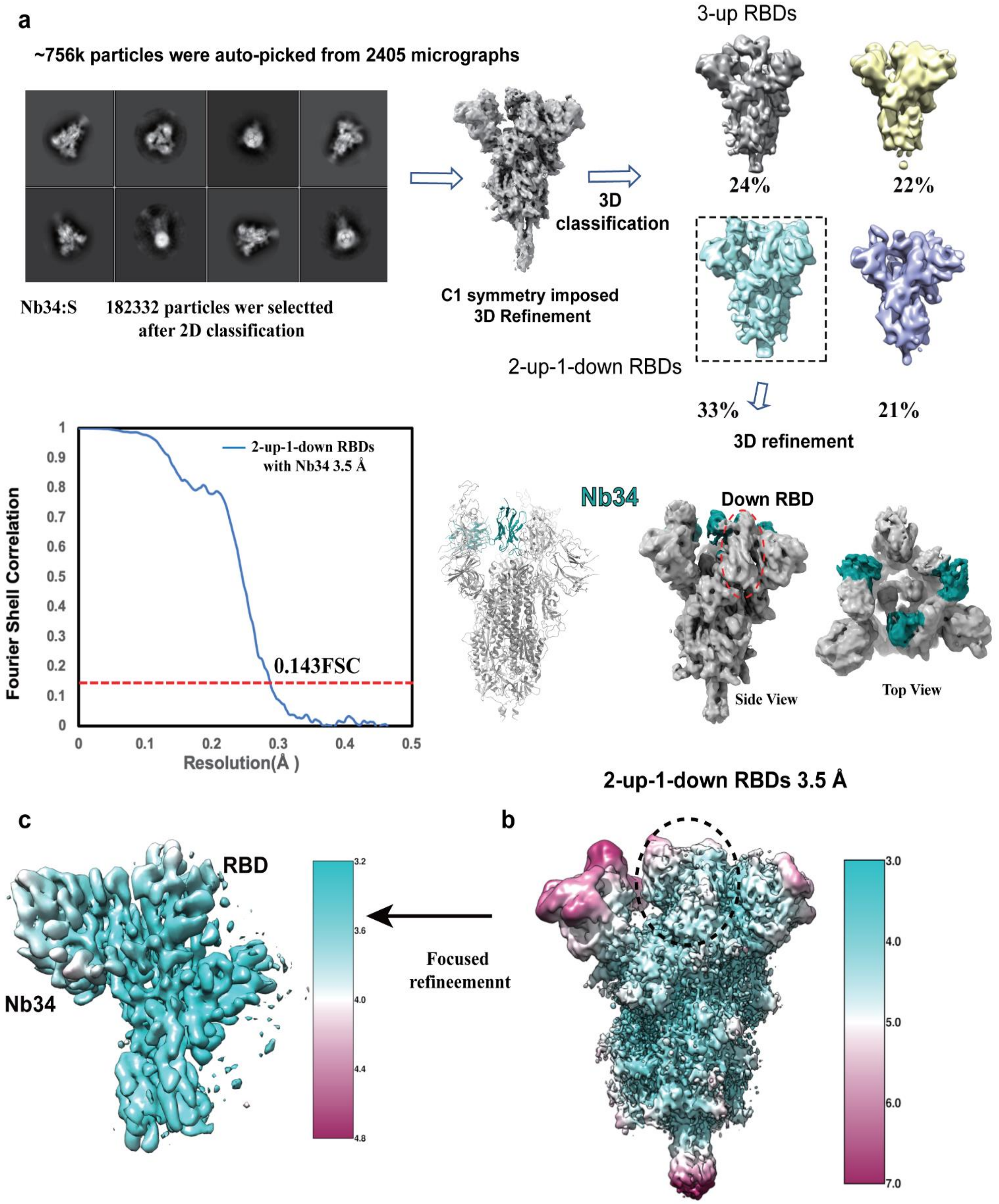

Supplementary Figure 7: Cryo-EM Structure determination of S with Nb34 and focused refinement.

a. ~756K particles were picked based on the 2D class averages of S with Nb34 for 3D classification. Two major classes were observed with clear features of 3-up RBDs and 2-up-1-down RBDs. We focused on the 2-up-1-down class for 3D refinement and obtained a structure with a global resolution of 3.5 Å corresponding to 0.143FSC shown at the lower-left panel.
b. Local resolution distribution for the S and Nb34 complex.
c. Focused Refinement of one down RBD with NB34. The down RBD showed better density compared to up RBDs.

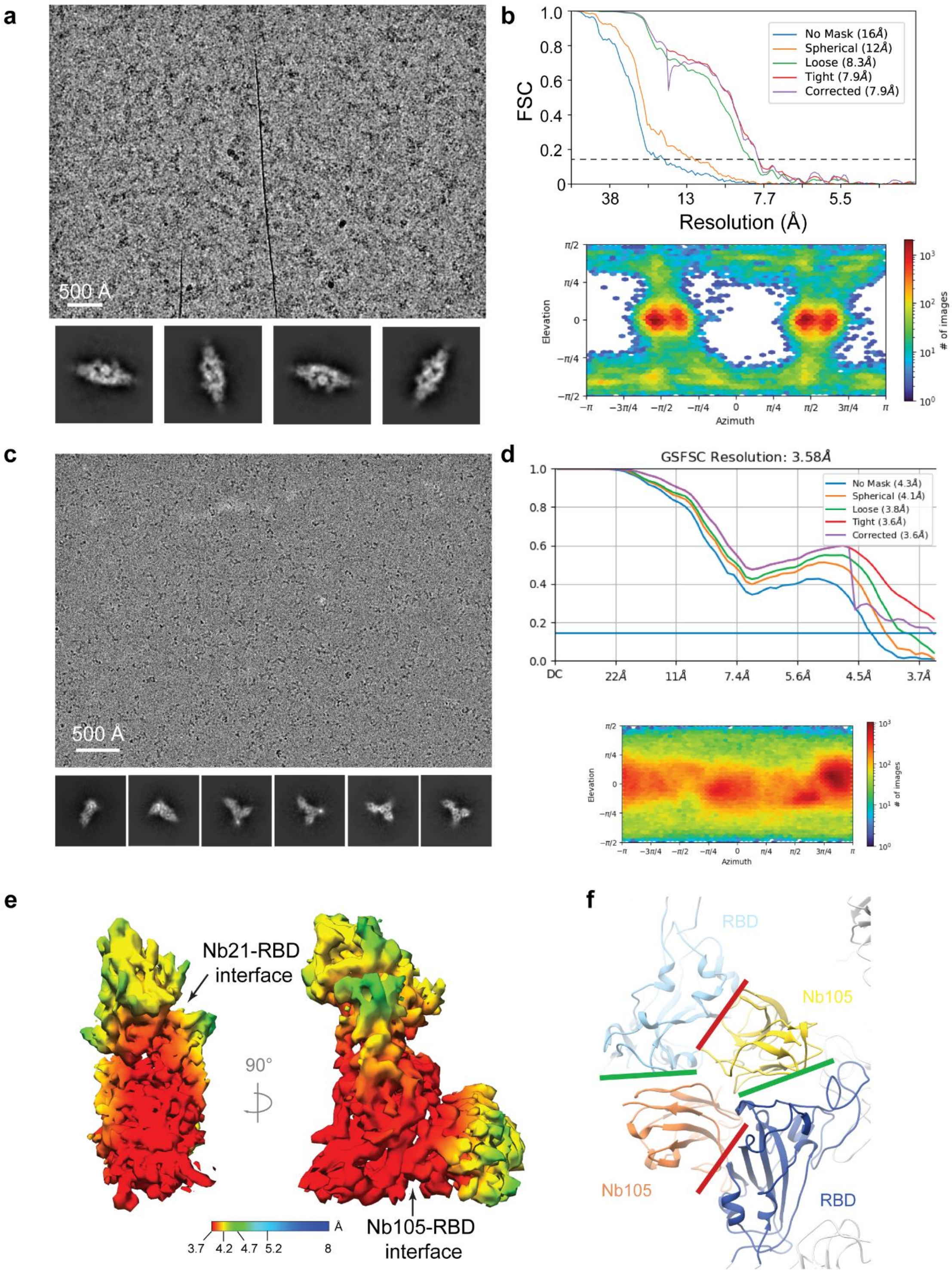

Supplementary Figure 8: Cryo-EM analysis of Nb105:S and Nb105:RBD: Nb21 complexes.

a. Representative micrograph and 2D class averages of Nb105:S complex.
b. Gold-standard Fourier shell correlation (FSC) and Euler angular distribution.
c. Representative micrograph and 2D class averages of Nb105:RBD:Nb21 complex.
d. Gold-standard Fourier shell correlation (FSC) and Euler angular distribution.
e. Local resolution estimation for Nb105:RBD:Nb21 complex.
f. Rigid docking of Nb105:RBD complex to the interface of the dimeric S. The interface highlighted with the green line is between the Nb framework and RBS.

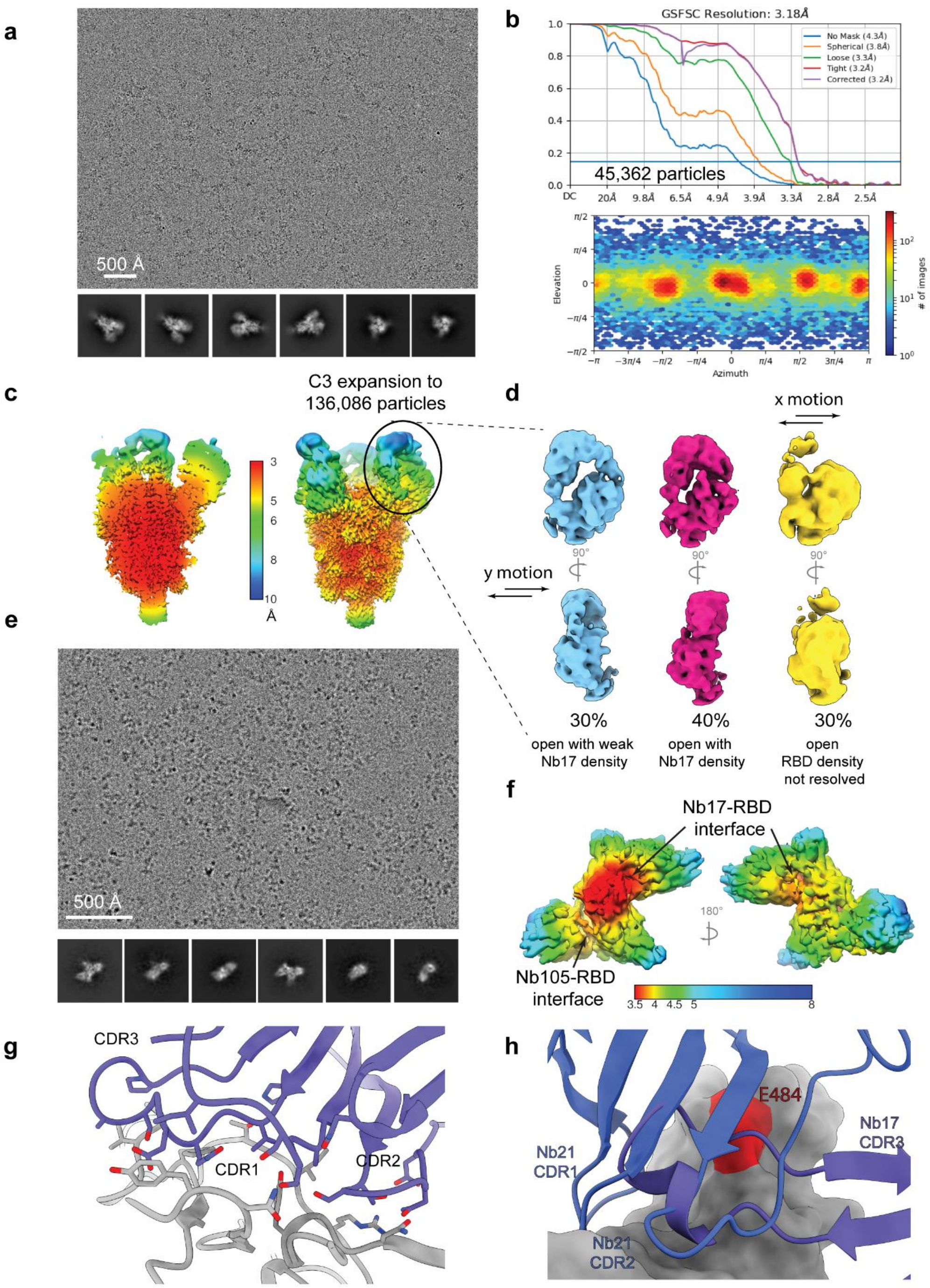

Supplementary Figure 9: Cryo-EM analysis of Nb17:S and Nb17:RBD:Nb105 complexes.

a. Representative micrograph and 2D class averages of Nb17:S complex.
b. Gold-standard Fourier shell correlation (FSC) and Euler angular distribution.
c. Local resolution estimation for Nb17:S complex.
d. Focused classification of the flexible region in Nb17:S complex. The density of Nb17 in class 1 (cyan) is smeared due to motion along the y-direction, class 2 (magenta) has well resolved RBD, Nb17, and NTD density, and both densities of RBD and Nb17 is lost due to motion along the x-direction.
e. Representative micrograph and 2D class averages of Nb17:RBD: Nb105 sample.
f. Local resolution estimation for Nb105:RBD: Nb21 sample.
g. Interface residues of Nb17:RBD complex.
h. Alignment of Nb17:RBD to Nb21:RBD showing the large overlap between Nb17 CDR3 with Nb21 CDR2 and partially Nb21 CDR1.

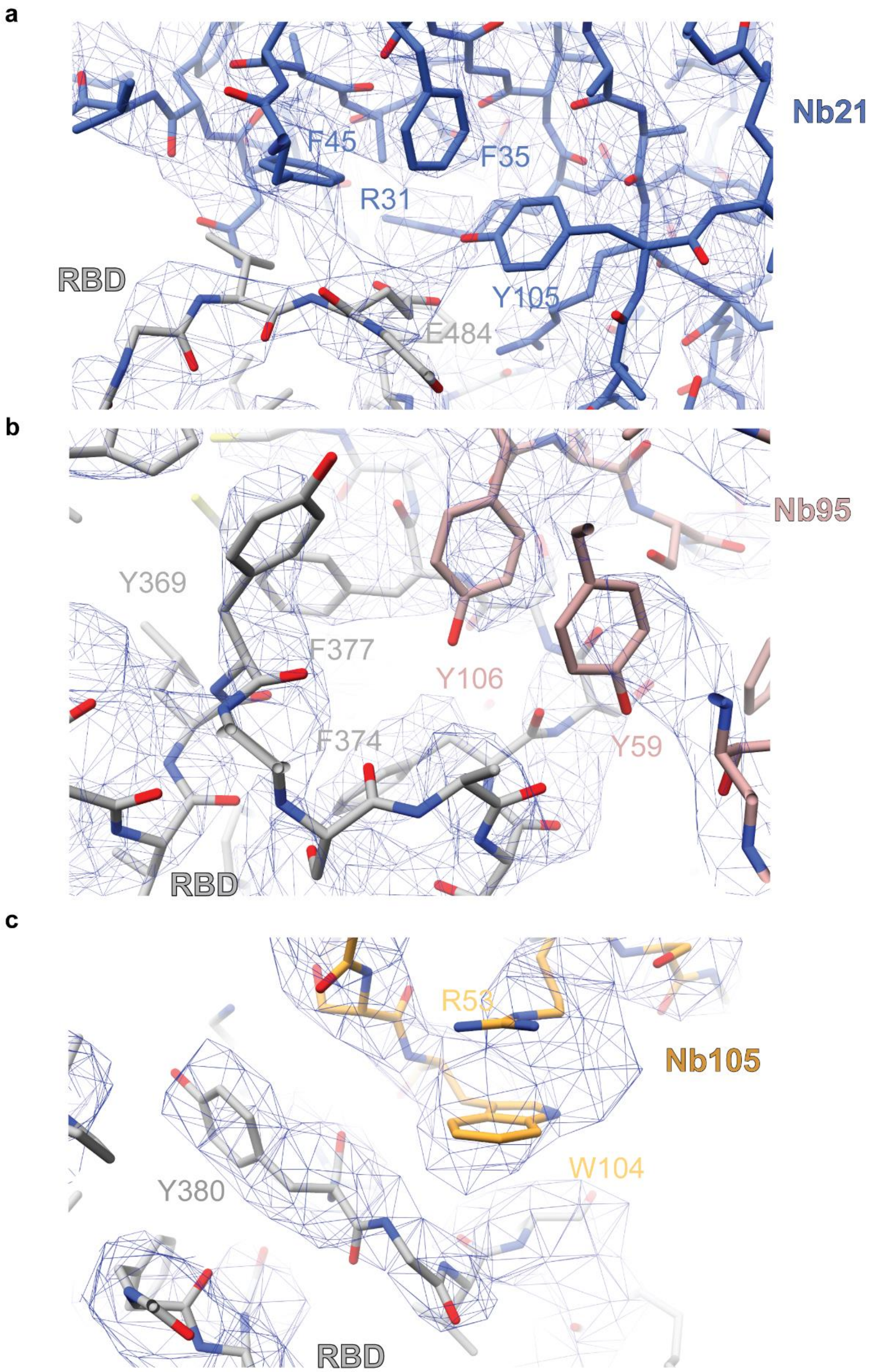

Supplementary Figure 10: Structure models with cryo-EM density for the interface region between RBD and Nbs after local refinement.

a. The density map of Nb21:RBD interactions.
b. The density map of Nb95:RBD interactions.
c. The density map of Nb105:RBD interactions.

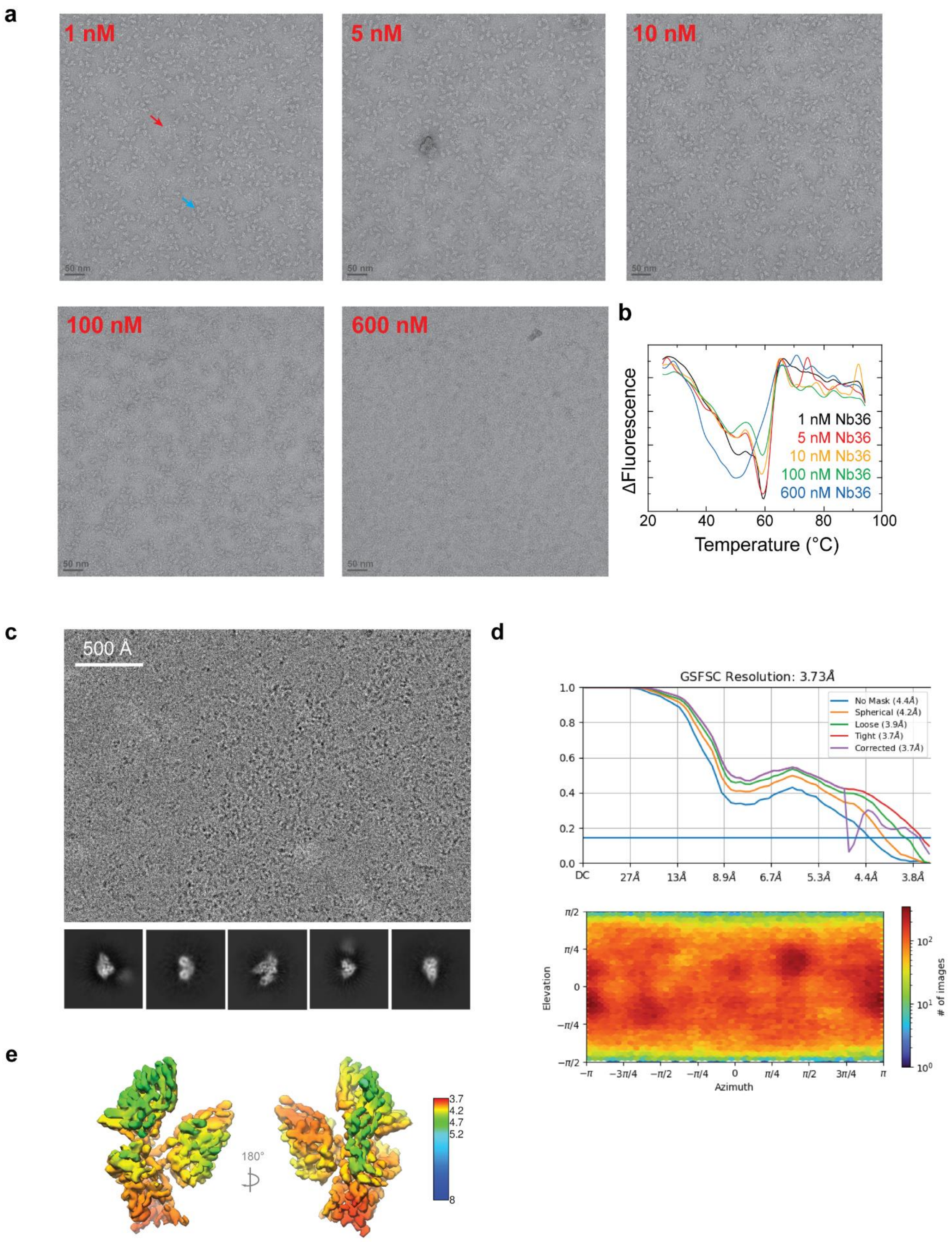

Supplementary Figure 11: EM Analysis of Nb36 with S and RBD.

a. Negative stain EM micrographs of spike protein in the presence of an increased concentration of Nb36. An example of an intact trimeric spike particle is highlighted by a blue arrow, and an example of a disrupted spike particle is highlighted by a red arrow. n = 2 biological replicates and representative image is shown here.
b. Thermal melting profile of S protein in the presence of an increased concentration of Nb36.
c. Representative micrograph and 2D class averages of Nb36:RBD: Nb21 complex.
d. Gold-standard Fourier shell correlation (FSC) and Euler angular distribution.
e. Local resolution estimation for Nb36:RBD: Nb21 complex.

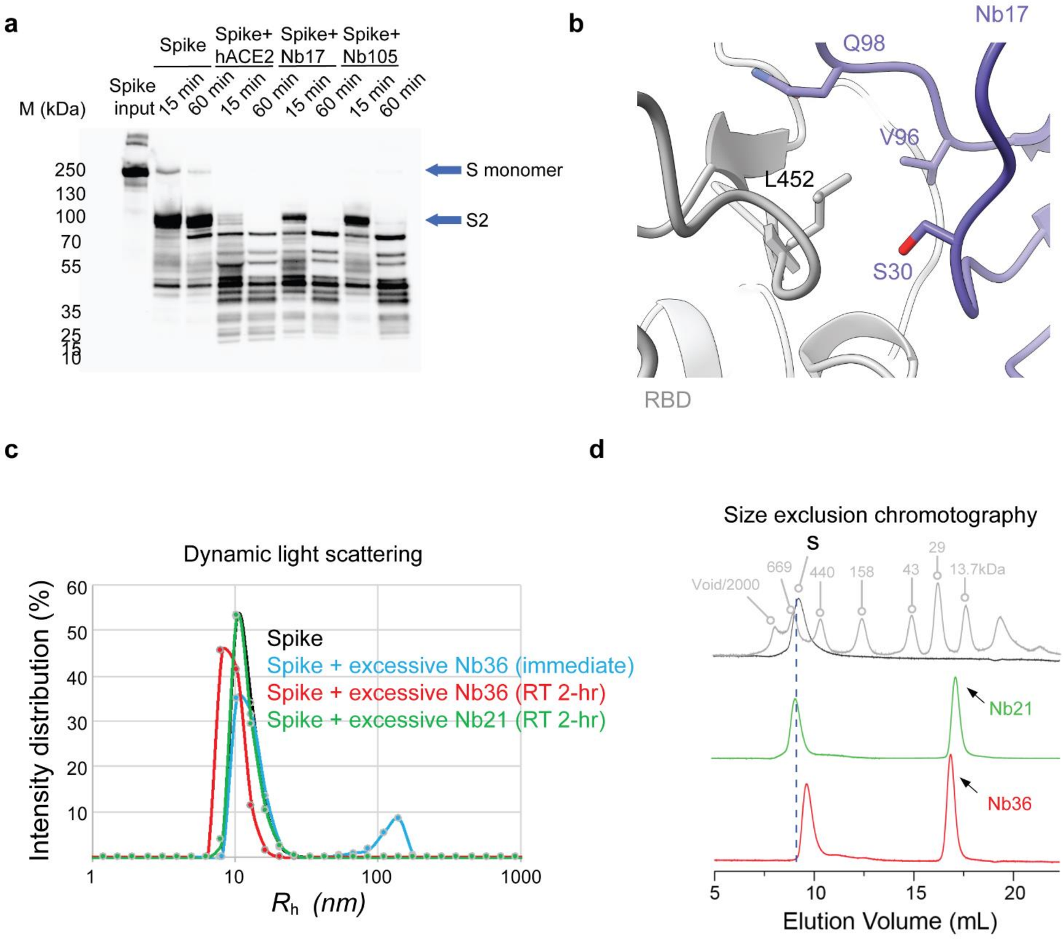

Supplementary Figure 12: Functional analysis of the neutralization mechanisms of class III Nbs.

a. Nb17 promotes the SARS-CoV-2 S transition to post-fusion state by Western Blot. The stable SARS-CoV-2 S trimer (hexapro) was digested with proteinase K either directly, or after incubation with hACE2 or Nbs for 15 min or 60 min at room temperature. anti-S2 SARS-CoV-2 polyclonal antibodies were used for western blot analysis. The experiments were repeated 4 times and representative picture is shown here.

b. Hydrophobic interactions formed between L452(RBD) and S30, V96, Q98(Nb17).

c. Hydrodynamic radius distribution by intensity for S, S immediately upon addition of Nb36, S with Nb36 incubated at room temperature for 2 hours and S with Nb21 at 0.6 mg/mL concentration of S and 6:1 molar ratio of Nbs in PBS buffer.

d. Size exclusion chromatography profiles of S (black), S with Nb21 (green) and S with Nb36 (red) with superdex 200 GL 10/300 column on Shimadzu HPLC at 0.25 mL/min flow rate in PBS buffer. The protein standard profile is shown in gray overlapping spike only profile.

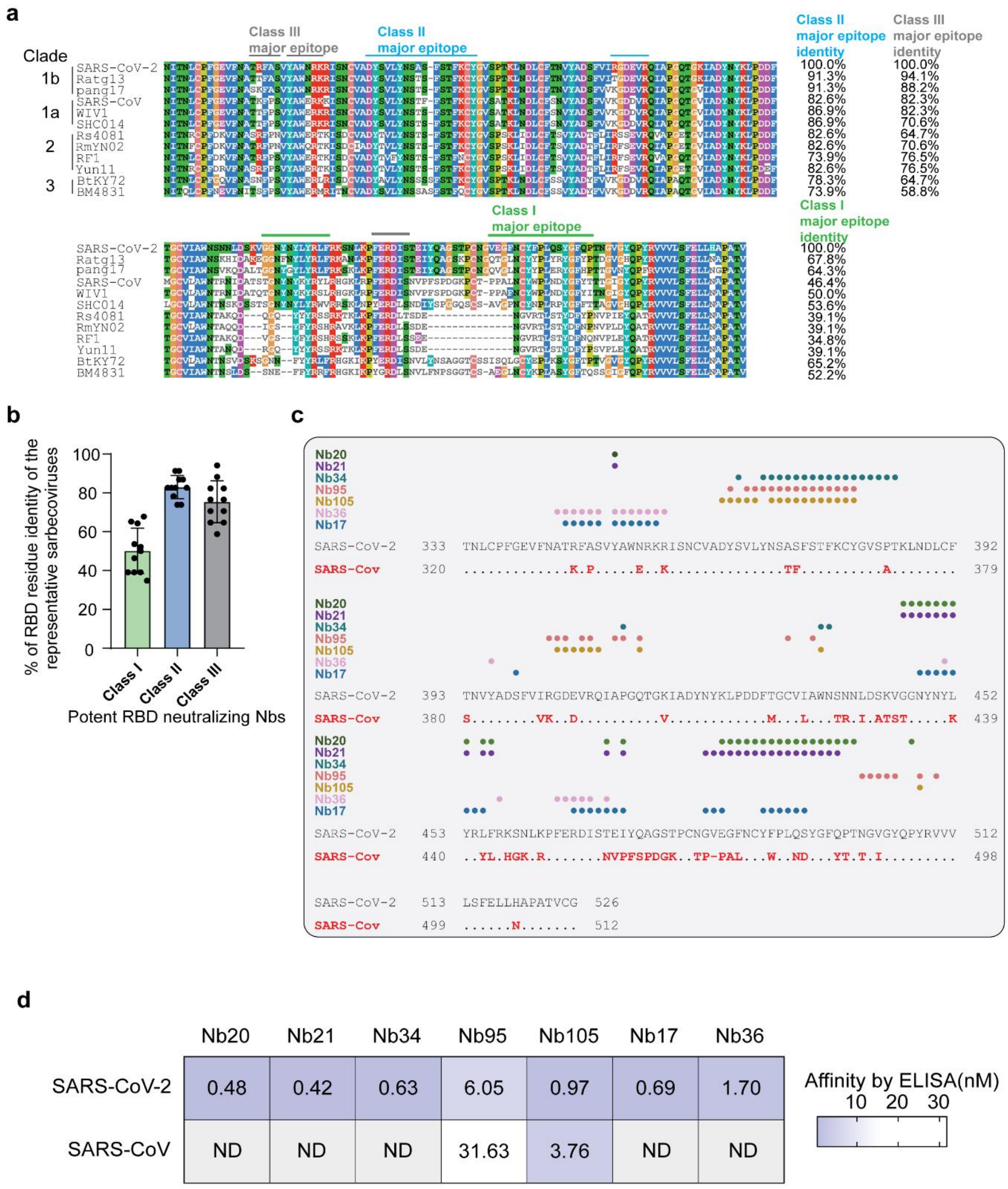

Supplementary Figure 13: Analysis of binding of 7 Nbs against RBD$_{\text{SARS-CoV}}$

a. RBD Sequence alignment from the Sarbecovirus family. Major epitopes of three classes of Nbs were highlighted and epitope identities on different RBDs were shown.
b. Analysis of RBD sequence identity of 12 representative sarbecoviruses for different classes of Nbs. Data are presented as mean +/- SD.
c. Sequence alignment of SARS-CoV-2 and SARS-CoV, with non-conserved SARS-CoV amino acid residues highlighted in red letters. Individual Nb epitope footprints on SARS-CoV-2 RBD are illustrated in color coded dots along the primary sequence.
d. Binding affinity (IC50) of different Nbs towards SARS-CoV and SARS-CoV-2 measured by ELISA. IC50 values reported in nM units. ND: signal not detected.

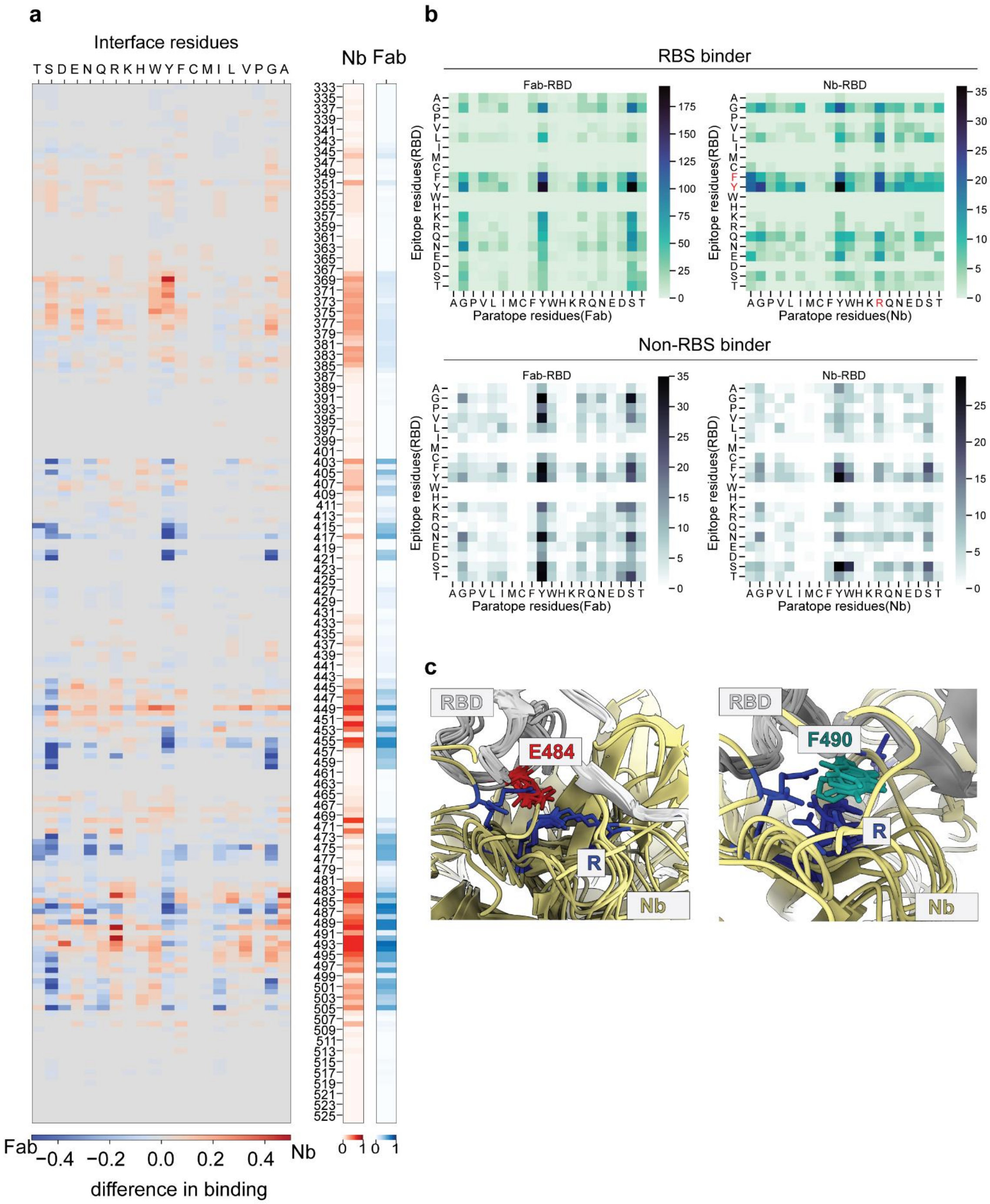

Supplementary Figure 14: Comparison of neutralizing Nbs and mAbs for RBD binding.

a. The heatmap shows the binding difference between Nbs and Fabs in terms of paratope residue utility despite overall similar epitope regions.
b. Heatmaps show the difference in preference of epitope-paratope residues between Nbs and Fabs. The comparisons were made separately for RBS binders and non-RBS binders. Nbs with at least 30% overlapping residues with ACE2 binding sites were considered RBS binders.
c. Illustrations of dominated electrostatic interactions formed between arginine from Nb CDRs and RBD residues. RBD was colored in dark gray, Nbs were colored in khaki, E484 (RBD) was colored in red, F490 (RBD) was colored in teal and R (Nb CDRs) was colored in blue.

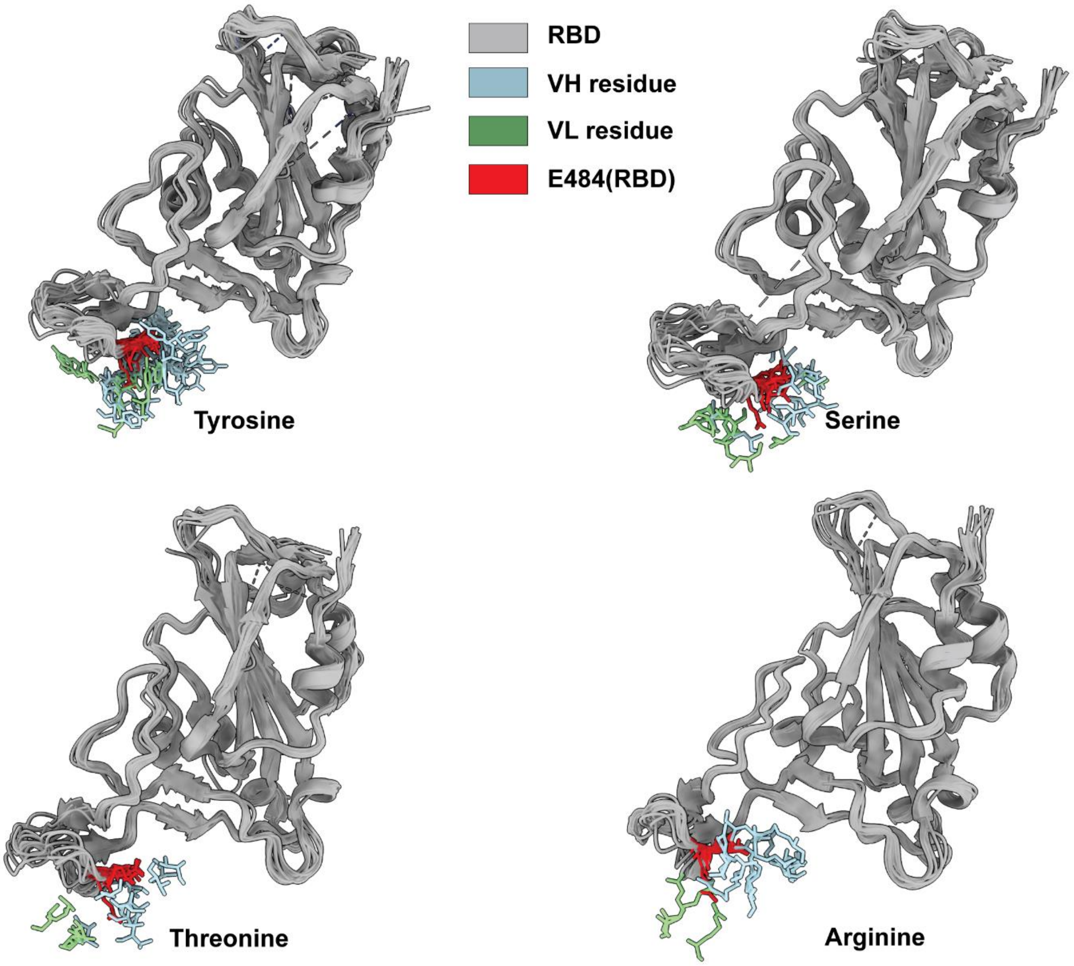

Supplementary Figure 15: Analysis of interactions of E484 (RBD) with neutralizing Nbs and mAbs. Superposition of Fab-RBD structures showing E484 (RBD) forms hydrogen and/or hydrophobic interactions with the respective residues of Fabs. The side chains of residues tyrosine, serine, threonine and arginine in close contact with E484 are shown in stick representation. RBD: dark gray, Fab VH: light blue, Fab LH: light green, and residue E484 (RBD): red.

Supplementary Table 1. Summary of dominant RBD mutations.

RBD Mutation	Region of Origin or Circulating Variant[1]	Increases ACE2 Binding	Replicated *in vitro* with ACE2 affinity Positive Pressure[2,3]	Humoral Immunity Resistance[4,5]	Replicated *in vitro* with Antibody Negative Pressure[6,7]
N501Y	B.1.1.7 UK<br>SA 501Y.V2<br>Brazil P.1	Yes[8,9]	Yes	mAbs (class 1 and 2) [10]<br>Convalescent Sera[11]<br>mRNA Vaccine elicited mAb	Yes
E484K/Q	SA 501Y.V2<br>Brazil P.1	Yes[12]	Yes	mAbs (class 1 and 2)<br>Convalescent Sera<br>mRNA Vaccine elicited mAb	Yes
K417N/T	SA 501Y.V2<br>Brazil P.1	No[12]	No	mAbs (class 1 and 2)<br>Convalescent Sera<br>mRNA Vaccine elicited mAb	Yes
N439K	Europe	Yes[2]	Yes	mAbs (class 3)<br>Convalescent Sera[13]<br>mRNA Vaccine elicited mAb	No
Y453F	Danish Mink-Associated Variant (Cluster 5) [14]	Yes[15,16]	Yes	mAbs[17]<br>Convalescent Sera	No
A475V	Australia	No	No	mAbs[18] (class 1)<br>Convalescent Sera[19]<br>mRNA Vaccine elicited mAb	Yes
L452R	California & India	Yes[21]	No	mAbs<br>Convalescent Sera<br>mRNA Vaccine elicited mAb, Sera[20,21]	Yes

Supplementary Table 2. Statistics for 3D reconstruction and model refinement for Nb:S complexes.

	Nb21:S<br>(EMD-24255)<br>(PDB: 7N9B)		Nb95:S<br>(EMD-24256)<br>(PDB: 7N9C)		Nb34:S<br>(EMD-24257)<br>(PDB: 7N9E)	Nb105:S<br>(EMD-23802)<br>(PDB: n/a)	Nb17:S<br>(EMD-24262)<br>(PDB: 7N9T)
**Data collection and processing**							
Microscope	Titan Krios		Titan Krios		Titan Krios	Titan Krios G3i	Titan Krios G3i
Camera	Falcon 3 EC		Gatan K3		Gatan K3	Gatan K3	Gatan K3 (CDS)
Voltage (keV)	300		300		300	300	300
Defocus range (m)	-0.5 to -3.5		-1.5 to -5.0		-1.5 to -3.0	-1 to -3	-0.5 to -2
Pixel size (Å)	0.83		1.06		1.06	1.069	1.07
Electron dose (e/Å^2)	62		60		60	51.5	40
Symmetry imposed	C1	C1	C1	C1	C1	C1	C1
Particles (no.)	97,000	91,000	85,549	68,378	60,614	113,230	45,362
Map resolution (Å)	3.57	3.86	3.76	3.71	3.52	7.92	3.18
B-factor (Å^2)	-94.15	-102.91	-94.48	-93.42	-79.21	-584.9	-45.3
Micrographs (no.)	2574		4761		2405	8417	2070
**Model statistics**							
Clash score	19.70		21.95		18.69		8.01
MolProbity score	2.00		2.20		2.03		2.21
Ramachandran plot (%)							
Outliers	0		0		0		0
Allowed	2.92		5.32		3.85		3.46
Favored	97.08		94.68		96.15		96.54
Rotamer outliers (%)	0		0		0		0.35

Supplementary Table 3. Statistics for 3D reconstruction and model refinement for 2Nbs:RBD complexes.

	Nb21:Nb105:RBD<br>(EMD-23782)<br>(PDB: 7MDW)	Nb21:Nb36:RBD<br>(EMD-23790)<br>(PDB: 7MEJ)	Nb17:Nb105:RBD<br>(EMD-23788)<br>(PDB: 7ME7)
**Data collection and processing**			
Microscope	Titan Krios	Titan Krios G3i	Titan Krios G3i
Camera	Gatan K3	Gatan K3 (CDS)	Gatan K3 (CDS)
Voltage (keV)	300	300	300
Defocus range (m)	-1.0 to -2.5	-0.5 to -2.5	-0.5 to -2.5
Pixel size (Å)	0.872	0.52	0.52
Electron dose (e/Å^2)	60.4	108	108
Symmetry imposed	C1	C1	C1
Particles (no.)	297,899	154,955	280,090
Map resolution (Å)	3.58	3.55	3.73
B-factor (Å^2)	-154.6	-119.5	-139.1
Micrographs (no.)	5152	7957	5499
**Model statistics**			
Clash score	5.68	7.94	4.23
MolProbity score	1.88	1.86	1.53
Ramachandran plot (%)			
Outliers	0	0	0
Allowed	6.57	6.54	4.65
Favored	93.47	93.46	95.35
Rotamer outliers (%)	0	0.28	0.56

Supplementary Table 4. Summary of structure features for IgGs and Nbs.

PDB	Type	name	method	cavity spike	cavity ab	Burid surface area	Burid surface area(mAb VH)	Burid surface area(mAb VL)	best matched fab	structural overlap
6XC2	mAb	CC12.1	x-ray	0.34	0.33	1318.53	801.94	588.37	\	\
6XC4	mAb	CC12.3	x-ray	0.37	0.34	886.29	703.64	182.65	\	\
6XCM	mAb	C105	cryoEM	0.41	0.42	906.17	666.91	267.88	\	\
6XDG	mAb	REGN10933	cryoEM	0.44	0.38	920.37	768.82	185.43	\	\
6XDG	mAb	REGN10987	cryoEM	0.46	0.46	593.52	502.12	114.17	\	\
6XE1	mAb	CV30	x-ray	0.38	0.37	1018.08	760.66	269.04	\	\
6XKP	mAb	CV07-270	x-ray	0.40	0.41	793.50	718.46	115.36	\	\
6XKQ	mAb	CV07-250	x-ray	0.44	0.31	881.33	415.23	552.53	\	\
6YLA	mAb	CR3022	x-ray	0.34	0.34	982.32	627.39	393.69	\	\
6ZCZ	mAb	EY6A	x-ray	0.34	0.35	952.18	602.64	448.45	\	\
7B3O	mAb	STE90-C11	x-ray	0.26	0.25	1146.01	678.54	473.66	\	\
7BWJ	mAb	7BWJ	x-ray	0.40	0.38	612.14	499.01	143.90	\	\
7BYR	mAb	BD23	cryoEM	0.50	0.40	770.34	741.37	32.32	\	\
7C01	mAb	CB6	x-ray	0.39	0.37	1069.45	743.58	339.23	\	\
7CAH	mAb	H014	cryoEM	0.35	0.41	957.79	715.74	317.74	\	\
7CDI	mAb	P2C-1F11	x-ray	0.40	0.37	945.31	755.28	225.26	\	\
7CDJ	mAb	P2C-1A3	x-ray	0.43	0.34	865.77	617.33	310.70	\	\
7CH4	mAb	BD604	x-ray	0.40	0.37	1116.10	764.24	375.07	\	\
7CH5	mAb	BD629	x-ray	0.39	0.39	1052.65	863.20	189.60	\	\
7CHB	mAb	BD236	x-ray	0.34	0.36	1088.77	708.52	431.29	\	\
7CHE	mAb	BD368-2	x-ray	0.47	0.38	672.52	592.83	134.98	\	\
7CJF	mAb	7CJF	x-ray	0.31	0.30	1178.00	794.10	421.88	\	\
7CM4	mAb	CT-P59	x-ray	0.43	0.38	921.91	849.78	119.00	\	\
7CWO	mAb	P17	cryoEM	0.47	0.39	850.06	660.87	243.02	\	\
7DPM	mAb	MW06	x-ray	0.30	0.41	735.36	551.16	265.97	\	\
7JMO	mAb	COVA2-04	x-ray	0.35	0.38	1140.37	807.10	377.61	\	\
7JMP	mAb	COVA2-39	x-ray	0.40	0.31	677.85	601.27	144.54	\	\
7JMW	mAb	COVA1-16	x-ray	0.37	0.44	802.57	655.12	156.94	\	\
7JV6	mAb	S2H13	cryoEM	0.58	0.47	415.82	185.93	234.66	\	\
7JVA	mAb	S2A4	cryoEM	0.37	0.40	770.57	408.23	475.75	\	\
7JW0	mAb	S304	cryoEM	0.42	0.46	379.56	163.49	217.79	\	\
7K43	mAb	S2M11	cryoEM	0.38	0.43	650.59	621.53	58.64	\	\
7K45	mAb	S2E12	cryoEM	0.48	0.34	649.72	468.85	236.41	\	\
7K8M	mAb	C102	x-ray	0.39	0.36	1050.93	821.04	253.50	\	\
7K8S	mAb	C002	cryoEM	0.45	0.41	891.12	740.33	196.77	\	\
7K8U	mAb	C104	cryoEM	0.66	0.42	383.30	250.00	133.30	\	\
7K8V	mAb	C110	cryoEM	0.49	0.56	603.73	262.92	343.92	\	\
7K8W	mAb	C119	cryoEM	0.46	0.35	854.54	569.78	290.49	\	\
7K8X	mAb	C121	cryoEM	0.47	0.33	787.68	735.75	81.82	\	\
7K8Z	mAb	C135	cryoEM	0.43	0.37	495.47	321.72	206.88	\	\
7K90	mAb	C144	cryoEM	0.39	0.43	767.00	701.49	99.34	\	\
7K9Z	mAb	52	x-ray	0.41	0.44	868.57	613.32	279.13	\	\
7K9Z	mAb	298	x-ray	0.49	0.36	663.38	417.02	299.81	\	\
7KFV	mAb	C1A-B12	x-ray	0.33	0.32	1145.32	841.85	392.73	\	\
7KFW	mAb	C1A-B3	x-ray	0.35	0.34	1115.55	795.66	381.12	\	\
7KFX	mAb	C1A-C2	x-ray	0.34	0.35	1152.14	857.09	362.81	\	\
7KFY	mAb	C1A-F10	x-ray	0.32	0.32	1119.83	809.80	373.11	\	\
7KLH	mAb	15033-7	x-ray	0.41	0.34	963.75	425.65	630.19	\	\
7KMG	mAb	LY-CoV555	x-ray	0.44	0.29	802.50	606.58	271.54	\	\
7KMH	mAb	LY-CoV488	x-ray	0.30	0.33	909.94	666.59	261.43	\	\
7KMI	mAb	LY-CoV481	x-ray	0.33	0.34	1085.25	648.13	468.26	\	\
7KS9	mAb	910-30	cryoEM	0.35	0.36	791.44	412.19	450.03	\	\
7KZB	mAb	CR3014	x-ray	0.33	0.46	694.07	246.91	472.54	\	\
7L0N	mAb	S309	x-ray	0.44	0.38	753.49	645.09	154.46	\	\
7L5B	mAb	2-15	x-ray	0.48	0.36	881.80	711.16	198.14	\	\
7LD1	mAb	DH1047	cryoEM	0.38	0.41	780.92	553.31	242.05	\	\
6YZ5	Nb	H11-D4	x-ray	0.36	0.34	660.12	\	\	7CHE	0.439
6ZH9	Nb	H11-H4	x-ray	0.43	0.39	617.57	\	\	7KMG	0.387
7A29	Nb	Sb23	cryoEM	0.44	0.42	576.16	\	\	7BWJ	0.384
7C8V	Nb	SR4	x-ray	0.34	0.31	763.60	\	\	7K8W	0.282
7C8W	Nb	MR17	x-ray	0.39	0.37	884.55	\	\	7CM4	0.498
7CAN	Nb	MR17-K99Y	x-ray	0.40	0.38	860.30	\	\	7CM4	0.490
7D2Z	Nb	SR31	x-ray	0.32	0.31	922.96	\	\	7JVA	0.286
7JVB	Nb	Nb20	x-ray	0.41	0.37	718.11	\	\	7KMG	0.451
7KGJ	Nb	Sb45	x-ray	0.37	0.39	997.68	\	\	7K8W	0.353
7KGK	Nb	Sb16	x-ray	0.40	0.40	1017.77	\	\	7CM4	0.389
7KLW	Nb	Sb68	x-ray	0.36	0.36	642.57	\	\	7JVA	0.366
7KKK	Nb	Nb6	cryoEM	0.30	0.36	786.31	\	\	7K90	0.491
7KKL	Nb	mNb6	cryoEM	0.36	0.33	916.41	\	\	7K8S	0.372
7KN5	Nb	E	x-ray	0.39	0.33	804.03	\	\	7L5B	0.428
7KN5	Nb	U	x-ray	0.33	0.38	654.74	\	\	7DPM	0.415
7KN6	Nb	V	x-ray	0.35	0.37	990.31	\	\	7DPM	0.465
7KN7	Nb	W	x-ray	0.35	0.42	662.30	\	\	7DPM	0.415
N/A	Nb	Nb105	cryoEM	0.36	0.46	586.16	\	\	7JVA	0.379
N/A	Nb	Nb17	cryoEM	0.33	0.31	921.20	\	\	6XKP	0.176
N/A	Nb	Nb21	cryoEM	0.39	0.39	849.72	\	\	7L5B	0.428
N/A	Nb	Nb34	cryoEM	0.37	0.41	702.07	\	\	7DPM	0.452
N/A	Nb	Nb36	cryoEM	0.34	0.45	499.17	\	\	6XKP	0.011
N/A	Nb	Nb95	cryoEM	0.37	0.44	896.98	\	\	7CAH	0.437

Supplementary References

1 Science Brief: Emerging SARS-CoV-2 Variants. (2021).

2 Starr, T. N. *et al.* Deep Mutational Scanning of SARS-CoV-2 Receptor Binding Domain Reveals Constraints on Folding and ACE2 Binding. *Cell* **182**, 1295-1310 e1220, doi:10.1016/j.cell.2020.08.012 (2020).

3 Zahradník, J. *et al.* SARS-CoV-2 RBD in vitro evolution follows contagious mutation spread, yet generates an able infection inhibitor. *bioRxiv*, 2021.2001.2006.425392, doi:10.1101/2021.01.06.425392 (2021).

4 Wang, Z. *et al.* mRNA vaccine-elicited antibodies to SARS-CoV-2 and circulating variants. *Nature*, doi:10.1038/s41586-021-03324-6 (2021).

5 Weisblum, Y. *et al.* Escape from neutralizing antibodies by SARS-CoV-2 spike protein variants. *Elife* **9**, doi:10.7554/eLife.61312 (2020).

6 Greaney, A. J. *et al.* Comprehensive mapping of mutations in the SARS-CoV-2 receptor-binding domain that affect recognition by polyclonal human plasma antibodies. *Cell Host Microbe*, doi:10.1016/j.chom.2021.02.003 (2021).

7 Greaney, A. J. *et al.* Complete Mapping of Mutations to the SARS-CoV-2 Spike Receptor-Binding Domain that Escape Antibody Recognition. *Cell Host Microbe* **29**, 44-57 e49, doi:10.1016/j.chom.2020.11.007 (2021).

8 Nelson, G. *et al.* Molecular dynamic simulation reveals E484K mutation enhances spike RBD-ACE2 affinity and the combination of E484K, K417N and N501Y mutations (501Y.V2 variant) induces conformational change greater than N501Y mutant alone, potentially resulting in an escape mutant. *bioRxiv*, 2021.2001.2013.426558, doi:10.1101/2021.01.13.426558 (2021).

9 Ahmed, W., Philip, A. M. & Biswas, K. H. Stable Interaction Of The UK B.1.1.7 lineage SARS-CoV-2 S1 Spike N501Y Mutant With ACE2 Revealed By Molecular Dynamics Simulation. *bioRxiv*, 2021.2001.2007.425307, doi:10.1101/2021.01.07.425307 (2021).

10 Barnes, C. O. *et al.* SARS-CoV-2 neutralizing antibody structures inform therapeutic strategies. *Nature* **588**, 682-687, doi:10.1038/s41586-020-2852-1 (2020).

11 Cele, S. *et al.* Escape of SARS-CoV-2 501Y.V2 variants from neutralization by convalescent plasma. *medRxiv*, 2021.2001.2026.21250224, doi:10.1101/2021.01.26.21250224 (2021).

12 Villoutreix, B. O., Calvez, V., Marcelin, A. G. & Khatib, A. M. In Silico Investigation of the New UK (B.1.1.7) and South African (501Y.V2) SARS-CoV-2 Variants with a Focus at the ACE2-Spike RBD Interface. *Int J Mol Sci* **22**, doi:10.3390/ijms22041695 (2021).

13 Thomson, E. C. *et al.* The circulating SARS-CoV-2 spike variant N439K maintains fitness while evading antibody-mediated immunity. *bioRxiv*, 2020.2011.2004.355842, doi:10.1101/2020.11.04.355842 (2020).

14 Oude Munnink, B. B. *et al.* Transmission of SARS-CoV-2 on mink farms between humans and mink and back to humans. *Science* **371**, 172-177, doi:10.1126/science.abe5901 (2021).

15 Bayarri-Olmos, R. *et al.* The SARS-CoV-2 Y453F mink variant displays a striking increase in ACE-2 affinity but does not challenge antibody neutralization. *bioRxiv*, 2021.2001.2029.428834, doi:10.1101/2021.01.29.428834 (2021).

16 Welkers, M. R. A., Han, A. X., Reusken, C. & Eggink, D. Possible host-adaptation of SARS-CoV-2 due to improved ACE2 receptor binding in mink. *Virus Evol* **7**, veaa094, doi:10.1093/ve/veaa094 (2021).

17 Hayashi, T., Yaegashi, N. & Konishi, I. Effect of RBD mutation (Y453F) in spike glycoprotein of SARS-CoV-2 on neutralizing antibody affinity. *bioRxiv*, 2020.2011.2027.401893, doi:10.1101/2020.11.27.401893 (2020).

18 Yi, C. *et al.* Key residues of the receptor binding motif in the spike protein of SARS-CoV-2 that interact with ACE2 and neutralizing antibodies. *Cell Mol Immunol* **17**, 621-630, doi:10.1038/s41423-020-0458-z (2020).

19 Li, Q. *et al.* The Impact of Mutations in SARS-CoV-2 Spike on Viral Infectivity and Antigenicity. *Cell* **182**, 1284-1294 e1289, doi:10.1016/j.cell.2020.07.012 (2020).

20 Liu, C., Ginn, H. M., Dejnirattisai, W., Supasa, P., Wang, B., Tuekprakhon, A., ... & Screaton, G. R. Reduced neutralization of SARS-CoV-2 B. 1.617 by vaccine and convalescent serum. *Cell*, doi:10.1016/j.cell.2021.06.020 (2021).

21 Li, Q., Wu, J., Nie, J., Zhang, L., Hao, H., Liu, S., ... & Wang, Y. The impact of mutations in SARS-CoV-2 spike on viral infectivity and antigenicity. *Cell*, **182**(5), 1284-1294, doi:10.1016/j.cell.2020.07.012 (2020).
