# Supplementary material for: Potent neutralizing nanobodies resist convergent circulating variants of SARS-CoV-2 by targeting diverse and conserved epitopes
Source: Nat Commun. 2021 Aug 3;12:4676. doi: 10.1038/s41467-021-24963-3 (PMC8333356; doi:10.1038/s41467-021-24963-3)
Supplement: Supplementary file 3 — Reporting Summary [file 41467_2021_24963_MOESM3_ESM.pdf]

Corresponding author(s): Dina Schneidman-Duhovny, Cheng Zhang, Wei Huang and Yi Shi

Last updated by author(s): Jul 13, 2021

Reporting Summary

Nature Portfolio wishes to improve the reproducibility of the work that we publish. This form provides structure for consistency and transparency in reporting. For further information on Nature Portfolio policies, see our Editorial Policies and the Editorial Policy Checklist.

Statistics

For all statistical analyses, confirm that the following items are present in the figure legend, table legend, main text, or Methods section.

n/a	Confirmed	
☐	☒	The exact sample size (*n*) for each experimental group/condition, given as a discrete number and unit of measurement
☒	☐	A statement on whether measurements were taken from distinct samples or whether the same sample was measured repeatedly
☐	☒	The statistical test(s) used AND whether they are one- or two-sided<br>*Only common tests should be described solely by name; describe more complex techniques in the Methods section.*
☐	☒	A description of all covariates tested
☐	☒	A description of any assumptions or corrections, such as tests of normality and adjustment for multiple comparisons
☐	☒	A full description of the statistical parameters including central tendency (e.g. means) or other basic estimates (e.g. regression coefficient) AND variation (e.g. standard deviation) or associated estimates of uncertainty (e.g. confidence intervals)
☐	☒	For null hypothesis testing, the test statistic (e.g. *F*, *t*, *r*) with confidence intervals, effect sizes, degrees of freedom and *P* value noted<br>*Give P values as exact values whenever suitable.*
☒	☐	For Bayesian analysis, information on the choice of priors and Markov chain Monte Carlo settings
☒	☐	For hierarchical and complex designs, identification of the appropriate level for tests and full reporting of outcomes
☒	☐	Estimates of effect sizes (e.g. Cohen's *d*, Pearson's *r*), indicating how they were calculated

Our web collection on statistics for biologists contains articles on many of the points above.

Software and code

Policy information about availability of computer code

Data collection	The cryo-EM data was collected using SerialEM 3.8, Latitude S and EPU v2; ELISA reading (Supplementary Figure 1) was collected from Thermo Fisher Multiskan GO; Luciferase reading (Supplementary Figure 3) was collected from the Luminometer. Protein shift assay was measured using real-time PCR instrument (StepOne). More detailed information can be found in the Method.
Data analysis	Cryo-EM data was analyzed using Relion 3.1, Resmap v1.1.5, DeepEMhancer (https://github.com/rsanchezgarc/deepEMhancer), CTFFIND 4.1.12 and CryoSPARC 3.0.0 and validated using MolProbity 4.5.1. Model refinement was using Coot version 0.9 and Phenix version dev-3951. Figure 1, Extended Fig.1 & 3 are analyzed by Prism Graphpad 9.0. MD simulation was done using CHARMM-GUI and SHAKE algorithm. CDR3 loop modeling was performed using in-house developed software NanoNet (not published yet). Energy contribution calculation was performed using MMPBSA.py module in AMBER18. Protein thermal shift assay was analyzed using EXCEL. More detailed information can be found in the Method.

For manuscripts utilizing custom algorithms or software that are central to the research but not yet described in published literature, software must be made available to editors and reviewers. We strongly encourage code deposition in a community repository (e.g. GitHub). See the Nature Portfolio guidelines for submitting code & software for further information.

Data

Policy information about availability of data

All manuscripts must include a data availability statement. This statement should provide the following information, where applicable:

- Accession codes, unique identifiers, or web links for publicly available datasets
- A description of any restrictions on data availability
- For clinical datasets or third party data, please ensure that the statement adheres to our policy

The cryo-EM maps have been deposited in the Electron Microscopy Data Bank under accession code EMD-24255, EMD-24256, EMD-24257, EMD-23802, EMD-24262, EMD-23782, EMD-23790, EMD-23788. The atomic coordinates for the deposited map have been deposited in the Protein Data Bank under accession code 7N9B, 7N9C, 7N9E, n/a (due to lack of resolution), 7N9T, 7MDW, 7MEJ, 7ME7.

Field-specific reporting

Please select the one below that is the best fit for your research. If you are not sure, read the appropriate sections before making your selection.

☒ Life sciences ☐ Behavioural & social sciences ☐ Ecological, evolutionary & environmental sciences

For a reference copy of the document with all sections, see nature.com/documents/nr-reporting-summary-flat.pdf

Life sciences study design

All studies must disclose on these points even when the disclosure is negative.

Sample size	The chosen of sample size for analysis and comparison was based on the number of available Nb or Fab structures related to SARS-CoV-2 in the PDB.
Data exclusions	No data was excluded from the experiments.
Replication	All replication attempts were successful and consistent. For ELISA (Supplementary Figure 1), three replicates were performed and mean value of each point was shown and fitted into the curve. For pseudovirus neutralization assay (Supplementary Figure 3), 2 biological replicates were performed with 3 technical measurements each, mean value of each point was shown and fitted into the curve. For western blot (Supplementary Figure 12a), experiment has been performed for four times and a representative gel picture was shown. The experiment of Nb36 disrupting spike (Supplementary Figure 11) was performed twice and the representative negative stain EM image was shown.
Randomization	Due to the nature of structure studies, no randomization is involved.
Blinding	Due to the nature of structure studies, no blinding is involved.

Reporting for specific materials, systems and methods

We require information from authors about some types of materials, experimental systems and methods used in many studies. Here, indicate whether each material, system or method listed is relevant to your study. If you are not sure if a list item applies to your research, read the appropriate section before selecting a response.

Materials & experimental systems

n/a	Involved in the study	
☐	☒	Antibodies
☐	☒	Eukaryotic cell lines
☒	☐	Palaeontology and archaeology
☒	☐	Animals and other organisms
☒	☐	Human research participants
☒	☐	Clinical data
☒	☐	Dual use research of concern

Methods

n/a	Involved in the study	
☒	☐	ChIP-seq
☒	☐	Flow cytometry
☒	☐	MRI-based neuroimaging

Antibodies

Antibodies used	Thermo fisher T7 tag polyclonal antibody, HRP (Cat# PA1-31449, Lot# VA2925356C, 1mg/ml); Thermo fisher Pierce goat-anti-rabbit IgG (H+L) polyclonal antibody, peroxidase conjugated (Cat# 31460, Lot# VJ313046, 0.8 mg/ml); Thermo fisher Pierce high sensitivity NeutrAvidin-HRP (Cat# 31030, Lot# VI312484A, 1mg/ml); Sino Biological SARS-CoV-2 spike S2 rabbit polyclonal antibody (Cat# 40590-T62, Lot# HD14JU1604).
Validation	The primary antibody (Sino Biological SARS-CoV-2 spike S2 rabbit polyclonal antibody) was validated for ELISA and WB analysis

Validation	according to the manufacturer.

Eukaryotic cell lines

Policy information about cell lines

Cell line source(s)	293T-hsACE2 (Integral Molecular, Cat# C-HA102, Lot# TA-072420-MC); HEK293-ES (Expression system, Cat# 94-007S, Lot# n/a); s9f insect cell (Expression system, Cat# 94-001F, Lot#n/a)
Authentication	293T-hsACE2 cell line was authenticated by flow cytometry using an ACE2 monoclonal antibody (R&D systems, Cat# MAB9332-100).HEK293-ES and S9f cells were not authenticated.
Mycoplasma contamination	293T-hsACE2 cell line was tested to be free of mycoplasma using the Sigma Aldrich MP0035-1KT kit. HEK293-ES and s9f cell lines were not tested for mycoplasma contamination but no indication of contamination was observed.
Commonly misidentified lines (See ICLAC register)	No commonly misidentified lines were used.
