# Supplementary material for: Potent neutralizing nanobodies resist convergent circulating variants of SARS-CoV-2 by targeting diverse and conserved epitopes
Source: Nat Commun. 2021 Aug 3;12:4676. doi: 10.1038/s41467-021-24963-3 (PMC8333356; doi:10.1038/s41467-021-24963-3)
Supplement: Supplementary file 2 — Peer Review File [file 41467_2021_24963_MOESM2_ESM.pdf]

REVIEWER COMMENTS

Reviewer #1 (Remarks to the Author):

In this manuscript, Sun et al present a structural basis for the binding of SARS-CoV-2 neutralizing nanobodies to spike of SARS-CoV-2. The antibodies were grouped into three types according to their binding epitopes as defined by cryo-EM structures. Notably, some of the nanobodies conferred resistance to current circulating SARS-CoV-2 variants. With the small size of nanobodies and their potential to be developed as inhalant, the reported nanobodies is in value of curing SARS-CoV-2.

The reviewer commends the authors on their structural biology approaches and their informative summary of current antibodies. However, given the thorough research regarding to the epitopes of neutralizing antibodies of SARS-CoV-2 and the overall state of the manuscript, this work is lack of creativity and light spot. At this point, the reviewer feels compelled to be very clear for considering of publication in Nature Communications.

Major comments:
1. The same group(s) reported the crystal structure of nb20 bound SARS-CoV-2 RBD in 2020 (Science 2020, 370: 1479-1484). Nb20 and nb21, which was reported in the current study, are both ultra-potent nbs and alike in epitope. Although cryo-EM structure of spike and nb21 reveals their interaction mode, the results are rather limited in advancing our understanding of mechanism for the ultrapotent feature of the class I nbs. Perhaps, detailed comparison of the paratope and binding energy of nb21 with nb20 and other RBS-binding nbs and mAbs should be addressed.
2. The interaction residues on SARS-CoV-2 RBD for class III nbs, such as R346, A348, F490, Q493 and S494 are semi-conserved or not conserved in SARS-CoV (K, P, W, N, and D, respectively in SARS-CoV as counterpart), in contrast with the authors clarification that the epitope for class III (especially nb17) is conserved. The authors didn't define the criterion for conservation in Figure 3a (no expression in methods neither). The authors should address these questions and might even be interested in determining the binding affinity of nb17 and nb36 to SARS-CoV-RBD and their neutralizing ability to SARS-CoV. These should be addressed experimentally and/or discussed.
3. The unique epitope of nb17 is very compelling. Binding of nb17 seems to lock the spike in all RBD-up conformations, which is necessary for ACE2 binding. Besides, nb17 completely doesn't compete with ACE2 for binding to RBD, which makes the neutralizing mechanism for nb17 highly mysterious and ambiguous. This is a key and critical point, so more work is necessary to further clarify the neutralizing mechanism of nb17.
4. The authors proposed a special neutralizing mechanism of nb36 by destabiling the spike protein. However, the evidence they provided is not that solid. Both the ns-EM figures and thermo-shift assay showed a sudden change when nb36 was added at a super high concentration (600nM) rather than a gradient-dependent change. It is highly recommended other experiments, such as SEC analysis of spike profile upon binding to nb36, be performed to address these questions. Also, there is no direct evidence showing that nb36 would be accommodated by RBD and neighbour NTD.

Other technique concerns and comments
1. The authors discussed the detailed interactions between nanobodies and RBD. However, the cryo-EM structures of spike and nanobodies were determined at overall resolutions range from 3.2-3.8 Å and the RBD and nbs might even be less well fitted. It is hard to discuss the polar interactions (range of bond length is 2.5-3.3 Å) using structures at such resolutions. Although the author emphasized the local refinements of the interactions, the reviewer would suggest providing the electronic map of RBD-nbs interface to make the description convincing.
2. There is no reference for the last sentence of paragraph 4 in page 2, 'The results contrast with recent investigations of a repertoire of neutralizing mAbs derived from convalescent and vaccine-elicited polyclonal sera, which are significantly affected by at least one of these strains.'. And to my knowledge, neutralizing mAbs from sera of both convalescent patients and vaccinee were reported to remain active against B.1.1.7 lineage (see ref. Nature 2021 Mar 8.doi: 10.1038/s41586-021-03398-2).
3. Detailed information about RBD62 is needed.
4. Line 3-6 of the 6th paragraph in page 3, 'For nb95, the CDR3 side chains of D99, K100, and S108

form ionic or hydrogen-bonding interactions with the side chains of K378 and Y380 and the main chain carbonyl group of S375 of RBD, respectively (Fig. 1d). R408 … with V407, V503 and Y508 of RBD' does not match the figure 1d. For instance, polar interactions between K100 and Y380, as well as S108 and main chain amine group of E112 were not found in figure 1d. On the contrary, an extra salt bridge between E112 and K378 was shown in figure 1d but not described in the text.
5. It is better to paint the ionic and hydrogen bonds in different colors.

Reviewer #2 (Remarks to the Author):

Sun and colleagues characterized seven nanobodies (Nbs) and found they exhibited varying levels of binding and neutralizing against a panel of SARS-CoV-2 variants. Nbs 20 and 21 were markedly affected by SA variant B.1.351 but marginally affected by UK variants B.1.1.7. The remaining five, however, remained similar potency to B.1.351. Binding analysis to mutated RBDs indicated that Nbs 20 and 21 relied on E484 while the remaining five are independent of E484, although RBD62 also disrupted binding of Nb17 and diminished binding of Nb95 and Nb36 while left Nbs 34 and 105 unaffected. The authors went further to resolved a total of 9 Nb-bound RBD/spike structures by high-resolution cryo-EM and revealed several potential antiviral mechanisms associated with epitope specificity. Accordingly, the authors categorized the seven Nbs into three classes. Class I including Nbs 20 and 21 exhibited the most potent neutralizing activity to the wildtype SARS-CoV-2 but failed to neutralize B1.351 due to E484K mutation. The other two classes bound to more conserved epitopes and maintained neutralizing activity. Class III Nbs 17 and 36 bound to previously unidentified cryptic epitopes that are inaccessible by bivalent antibodies derived from human. These results provide a fresh and comprehensive insights into how Nbs targeted the spike in a unique manner to achieve their broad neutralizing activity against the emerging variants of concerns of SARS-CoV-2.

Obviously, a timely and relevant study, with real importance for the broader community and potential clinical applications. While there have been other publications on the effect of the Variants of Concern (VOC) on Nbs neutralization, the authors here conduct a more thorough and detailed characterization on the structure basis for the observed differences in neutralizing potency and breadth.

My one concern is the breath of Nbs claimed by the authors as they only tested two VOCs B.1.1.7 and B.1.351 where there are at least 7 VOCs have so far been identified. It is important to test the seven Nbs against other strains so we could have better understanding on the true breadth of their neutralizing activity.

For readers to better understand the seven Nbs, it is necessary to include a few introductory sentences as where these Nbs came from and how they were selected in the first place. What immunogen used to immunize animals and what antigen used to fish out these Nbs.

Table 1, how -1000.00 or <-1000.00 were determined? What is B62? Is it RBD62? Is so, please keep the nomenclature consistent thoroughout.

Extended Fig. 5a, how relative binding energy was calculated for each residue? In Fig.5b, why R31D was selected for analysis and what about other residues? Was the RBD used in the binding derived from the wildtype or any specific variant?

Reviewer #3 (Remarks to the Author):

SARS-CoV-2 is evolving continuously, generating convergent variants resistant to convalescent or vaccine-elicited sera. Nanobodies (Nbs) are stable and cost-effective therapeutic option. In this work, the authors reported 9 Nb-bound structures, based on which three classes are identified. The antiviral mechanisms and neutralizing epitopes for these Nbs were analyzed and discussed. The authors also compared Nbs with other neutralizing Abs and revealed the unique characteristics for Nbs. This work

provided valuable insights into the antiviral mechanisms of Nbs. The following are the points for this work.

1. Nb105 forms an elongated S/Nb105/S complex with two copies of the S protein. This is interesting. Why does Nb105 crosslink two copies of the S protein with only one binding interface? The authors should solve the structure for this elongated complex. Or, can the authors generate a model for the S/Nb105/S complex based on the binding interface between Nb105 and RBD?
2. The map quality of some cryo-EM maps is poor and should be improved, including the focused refined maps on the interface between Nb95 or Nb34 and RBD, the maps of Nb21/RBD/Nb105 and Nb17/RBD/Nb105.
3. Page 4, "While class II Nbs do not interact with the ACE2 directly, they can still efficiently block ACE2 binding at low nM concentrations (Fig. 2h)." I think this should be "While class II Nbs do not compete with the ACE2 directly … "
4. For Fig 1 legend, please explain the meaning of the minus fold change.

We appreciate the reviewers for the positive and constructive comments on our paper. In the revised manuscript, we have fully addressed their concerns by performing new experiments and analyses. The main text has been modified to improve clarity. In addition, we have expanded the main figures to 7 figures and the extended data figures to 15 (supplementary figures). By making these changes, we are pleased that this manuscript has been substantially improved. A point-to-point response has been provided as follows:

Major comments:

1. The same group(s) reported the crystal structure of nb20 bound SARS-CoV-2 RBD in 2020 (Science 2020, 370: 1479-1484). Nb20 and nb21, which was reported in the current study, are both ultra-potent nbs and alike in epitope. Although cryo-EM structure of spike and nb21 reveals their interaction mode, the results are rather limited in advancing our understanding of mechanism for the ultrapotent feature of the class I nbs. **Perhaps, detailed comparison of the paratope and binding energy of nb21 with nb20 and other RBS-binding nbs and mAbs should be addressed.**

> To explore the potential correlation between binding energy and affinity, we calculated the statistically optimized atomic potentials (SOAP) for protein-protein interfaces [1] of 13 class I Nbs for RBD binding. Our analysis (see below) reveals that the statistical binding energy calculated by SOAP is not sufficiently sensitive (or with sufficient resolution) to inform affinity. This is likely because the SOAP was optimized to better differentiate between correct and incorrect docking models. Indeed, our analysis reveals a weak and negative correlation between K_D and the SOAP score, instead of a positive correlation as expected.

Moreover, we have calculated a large number of interface scores using the CCharPPI web server for computational characterization of protein-protein interactions from structure[2]. We could not find any significant correlation using these scores. Benchmarking affinity prediction, based on the structures of protein-protein interactions, revealed that accurate prediction of affinity is still beyond the reach of current statistical methods & scoring functions [3]

Finally, we have used MD simulation to evaluate multiple different class I Nbs, and consistently, the results (binding energy) can not be used for affinity prediction. Although it is difficult to understand the ultrapotency of Nbs20 and 21 based on calculated energy, our detailed biophysical and structural analysis have revealed that both high-affinity (especially the exceptionally slow off rates for RBD binding) and epitope are critical to sterically block ACE2 binding to RBD, which is likely critical for neutralization.

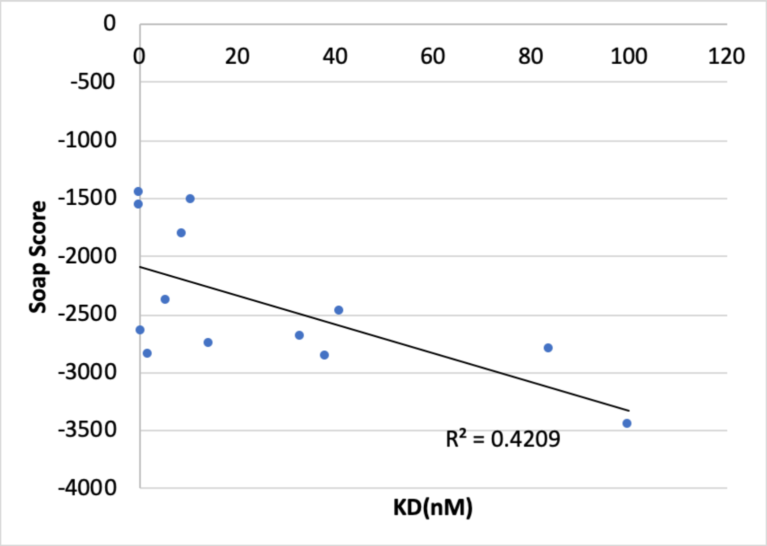

2. The interaction residues on SARS-CoV-2 RBD for class III nbs, such as R346, A348, F490, Q493 and S494 are semi-conserved or not conserved in SARS-CoV (K, P, W, N, and D, respectively in SARS-CoV as counterpart), in contrast with the authors clarification that the epitope for class III (especially nb17) is conserved. The authors didn't define the criterion for conservation in Figure 3a (no expression in methods neither). The authors should address these questions and might even be interested in determining the binding affinity of nb17 and nb36 to SARS-CoV-RBD and their neutralizing ability to SARS-CoV. These should be addressed experimentally and/or discussed.

> Thanks for the suggestion that helps us strengthen the paper. The criterion for the RBD conservation map (Fig 5a) was added to the Method. In brief, the conservation scores were obtained from Consurf server by querying the RBD sequence [4]. The multiple sequence alignment of different RBDs was constructed and the evolutionary rate was calculated using an empirical Bayesian method. The evolutionary rate was then normalized by the z-score method to calculate the conservation score, where higher scores indicate more conservation and lower scores indicate more variability.

We agree with this reviewer that class III Nbs, especially Nb17 targets a relatively less conserved epitope than class II Nbs. To quantitatively assess the epitope conservation, we selected and aligned 12 RBDs from the major clades of the sarbecovirus family (i.e., lineage B of beta-coronavirus or SARS-like). Major RBD epitope residues for each class of Nbs, and the epitope sequence identities to $RBD_{SARS\text{-}CoV\text{-}2}$ were shown in Extended Fig 13a, 13b. The median epitope identities for class I, II and III Nbs are 50% (σ= 11.1%), 82.6% (σ=5.7%), and 76.5% (σ=10.3%), respectively.

We also evaluated the binding of different potent neutralizing Nbs to $RBD_{SARS\text{-}CoV}$, which shares ~73% sequence identity with $RBD_{SARS\text{-}CoV\text{-}2}$. Consistent with epitope conservation analysis, the ELISA results show that unlike class I and III Nbs, potent neutralizing class II Nbs (specifically, Nbs 95 and 105, but not Nb 34) bind strongly to $RBD_{SARS\text{-}CoV\text{-}2}$ by targeting highly conserved RBD epitopes (Extended Fig. 13c, 13d). It is possible that specific and ultrapotent Class II Nbs may be used for the further bioengineering of pan-sarbecovirus Nb constructs.

We added a new Extended Fig. 13 (see below) during revision.

Extended Data Fig. 13

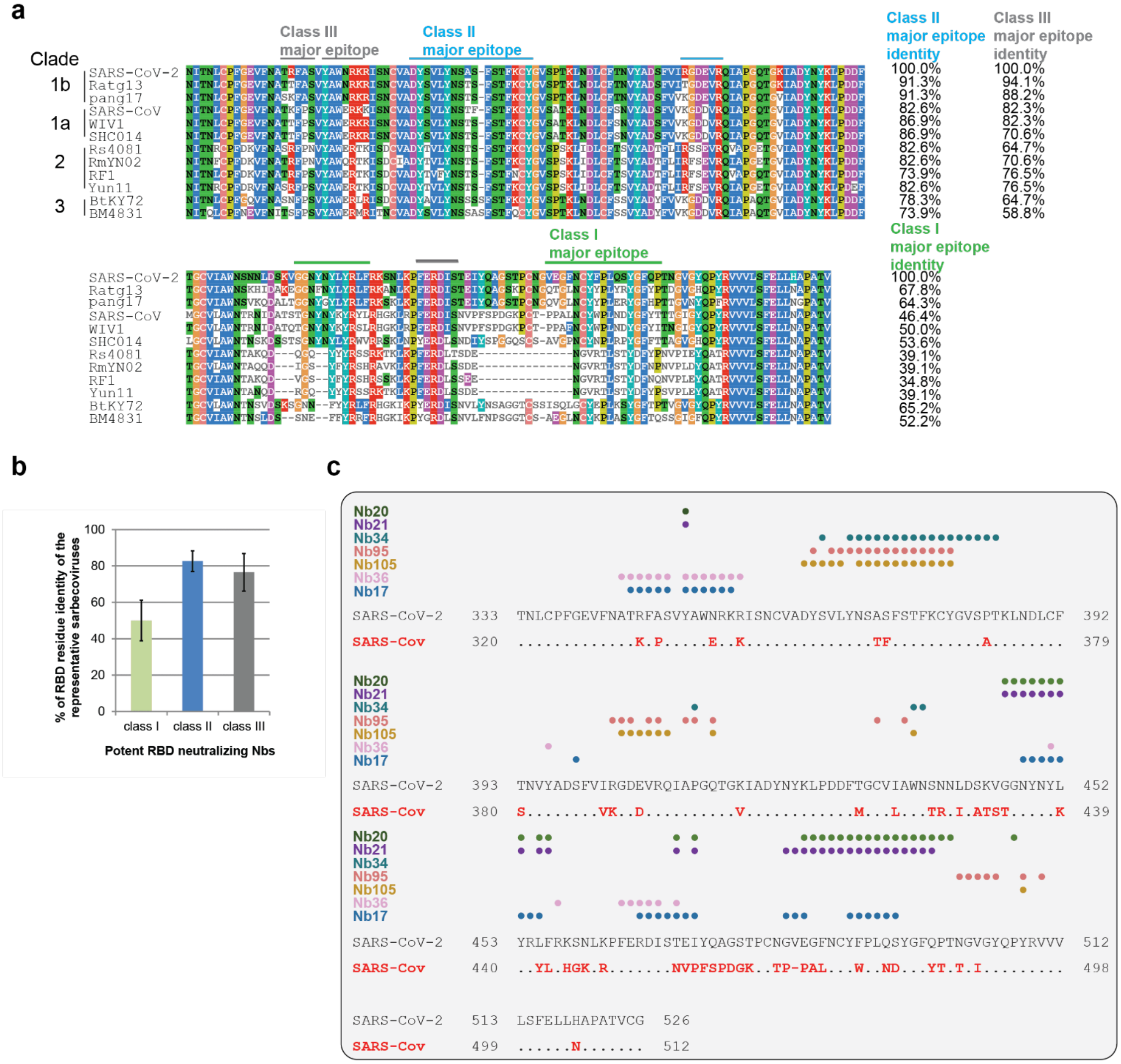

d

	Nb20	Nb21	Nb34	Nb95	Nb105	Nb17	Nb36
SARS-CoV-2	0.48	0.42	0.63	6.05	0.97	0.69	1.70
SARS-CoV	ND	ND	ND	31.63	3.76	ND	ND

Affinity by ELISA(nM)
10 20 30

*3. The unique epitope of nb17 is very compelling. Binding of nb17 seems to lock the spike in all RBD-up conformations, which is necessary for ACE2 binding. Besides, nb17 completely doesn't compete with ACE2 for binding to RBD, which makes the neutralizing mechanism for nb17 highly mysterious and ambiguous. This is a key and critical point, so more work is necessary to further clarify the neutralizing mechanism of nb17.

> We agree with this reviewer that Nb17 seems to trap S in a specific, all-RBD up conformation. We hypothesize that this unique S conformation implies that Nb17 binding may lead to premature activation of the spike making it more susceptible to protease activities [5-7]. To test this hypothesis and to understand the mechanism of Nb17 neutralization better, we performed a limited proteolysis experiment by digesting S (specifically a prefusion-stabilized SARS-CoV-2 Spike 6p developed by McLellan and colleagues) in complex with Nb17 by using proteinase K (Methods). S by itself, or in complex with hACE2 or Nb105 (an epitope II Nb) was used for control. We found that the super stable S is highly resistant to the proteinase K activity. After 60 min digestion, there was still residual undigested S as shown by the western blot analysis using anti-S2 polyclonal antibodies. The binding of Nb17 to S induces specific 3-RBD-up conformation that is more susceptible to the protease activity than S or the Nb105:S complex (comparable to the hACE2 binding to S). We speculate that the result might be more pronounced by using a wild-type glycoprotein S, which is highly flexible. We have also evaluated the potential effects using a cell-based pseudovirus post-fusion assay. However, due to technical limitations (low signal-to-noise/ high background), we were unable to obtain reproducible data. The result was inserted into the main text. The new figure was updated as Extended Data Fig. 12a (see below).

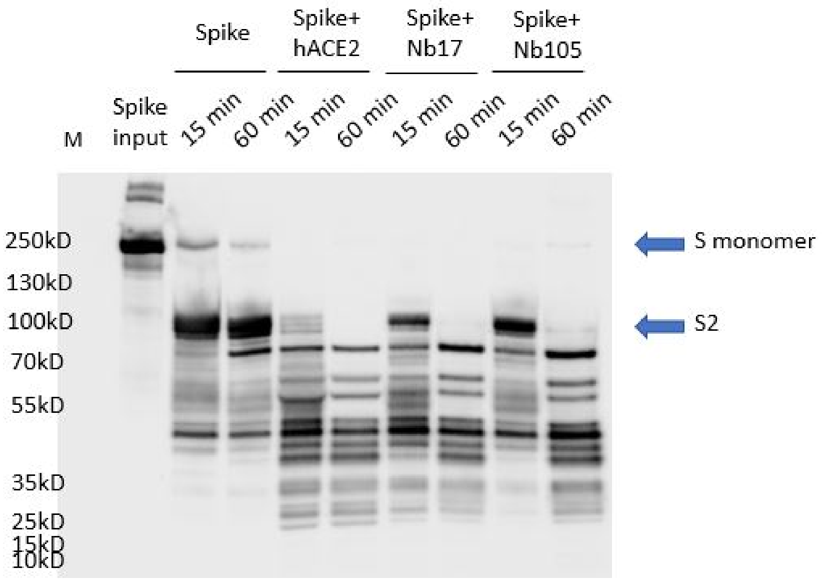

*4. The authors proposed a special neutralizing mechanism of nb36 by destabiling the spike protein. However, the evidence they provided is not that solid. Both the ns-EM figures and thermo-shift assay showed a sudden change when nb36 was added at a super high concentration (600nM) rather than a gradient-dependent change. It is highly recommended other experiments, such as SEC analysis of spike profile upon binding to nb36, be performed to address these questions. Also, there is no direct evidence showing that nb36 would be accommodated by RBD and neighbour NTD.

> To validate our observation that Nb36 can destabilize S, we performed additional experiments according to the reviewer. First, we used the SEC to evaluate Nb36:S complex. As shown in Extended Data Fig. 12d, Nb36 binding to S leads to the formation of a smaller complex than Nb21:S and critically S itself. This data indicates that Nb36 binding may disrupt the integrity of the trimeric S. Second, we performed an orthogonal Dynamic light scattering (DLS) experiment which further supports the SEC result (Extended Data Fig. 12c). Finally, we note that despite using the stable Hexapro S, Nb36 binding to S can blur the image at or below 100 nM, which is consistent with the *in vitro* pseudovirus neutralization potency (~ 7 nM) with the WT spike (Extended Data

Fig.3g). Together, our experiments suggest that Nb36 can efficiently neutralize SARS-CoV-2 by destabilizing the spike. These experiments were added to the revised manuscript in Extended Data Fig. 12c-d.

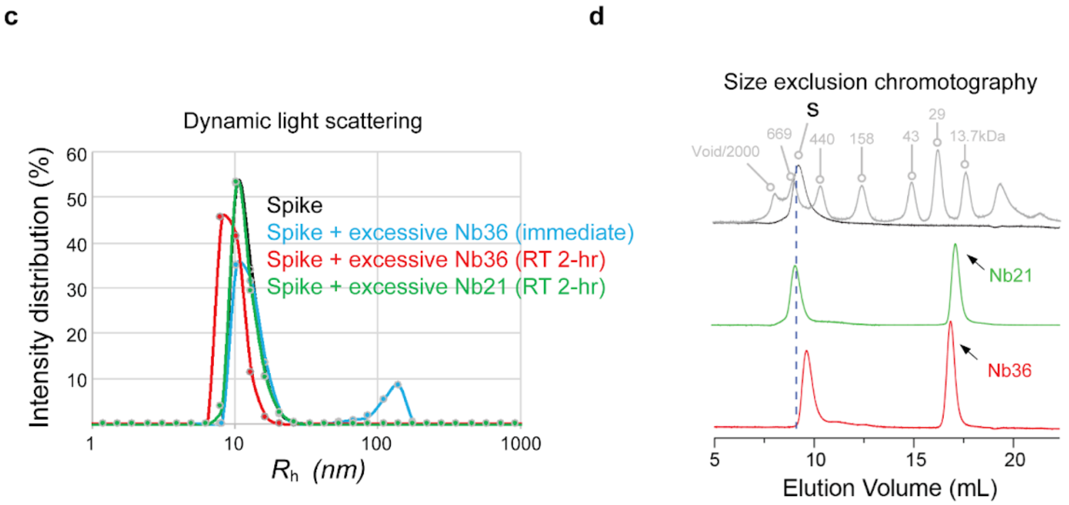

* Also, there is no direct evidence showing that nb36 would be accommodated by RBD and neighbour NTD.

> Due to technical limitations (i.e., spike destabilization in complex with Nb36), we can not obtain high-quality images of Nb36 in complex with the full-length S to directly visualize NTD. Our hypothesis was based on the superposition of Nb36 into an open S structure (PDB # 7CAK). We agreed with the reviewer that despite extensive efforts, we still do not have direct imaging evidence that Nb36 would be accommodated by RBD and the neighbor NTD. In the revised manuscript, we have further tuned down this claim.

“Facilitated by the small size, Nb36 may insert its convex paratope residues between an RBD and the adjacent NTD to destabilize the spike.”

“To validate this hypothesis, we employed analytical SEC to check the sizes of Nb:RBD complexes. Our analysis reveals that Nb36 binding to S leads to the formation of a smaller complex than the Nb21:S complex and interestingly the S itself (Extended Data Fig. 12d). Dynamic light scattering (DLS) was used to further substantiate the negative stain and SEC results. After two hours of incubation at room temperature, the Nb36:S complex showed a substantially smaller radius (Rh) than the Nb21: S complex, which has an identical radius with the transiently formed Nb36: S complex (Extended Data Fig. 12c). Together, these data suggest that Nb36 can efficiently neutralize SARS-CoV-2 by destabilizing the spike. Since a super stable S variant (HexaPro) was used for this study, it is anticipated that Nb36 binding may have a more dramatic impact on the highly flexible wild-type spike [8]. Moreover, this destabilization mechanism is a reminiscence of mAb CR3022. However, Nb36 targets a completely different epitope from CR3022 with substantially higher neutralization potency [9,10]. “

Other technique concerns and comments

1. The authors discussed the detailed interactions between nanobodies and RBD. However, the cryo-EM structures of spike and nanobodies were determined at overall resolutions range from 3.2-3.8 Å and the RBD and nbs might even be less well fitted. It is hard to discuss the polar interactions (range of bond length is 2.5-3.3 Å) using structures at such resolutions. Although the author emphasized the local refinements of the interactions, the reviewer would suggest providing the electronic map of RBD-nbs interface to make the description convincing.

> We have performed further refinement on the local maps of Nbs with RBD to improve map quality. We have now included Extended Data Fig. 10 in the supplementary information to show EM maps of RBD and Nb interfaces allowing us to assign side chains for bulky residues, such as Trp, Tyr, and Phe of Nb21, Nb95, and Nb105.

Our discussion of polar interactions was based on the potential favorable positions of polar residues, after registering bulky residues to the density maps. We have moderated the claim during revision.

The density maps were shown in the Extended Data Fig. 10: a (Nb21), b(N95), and c(Nb105).

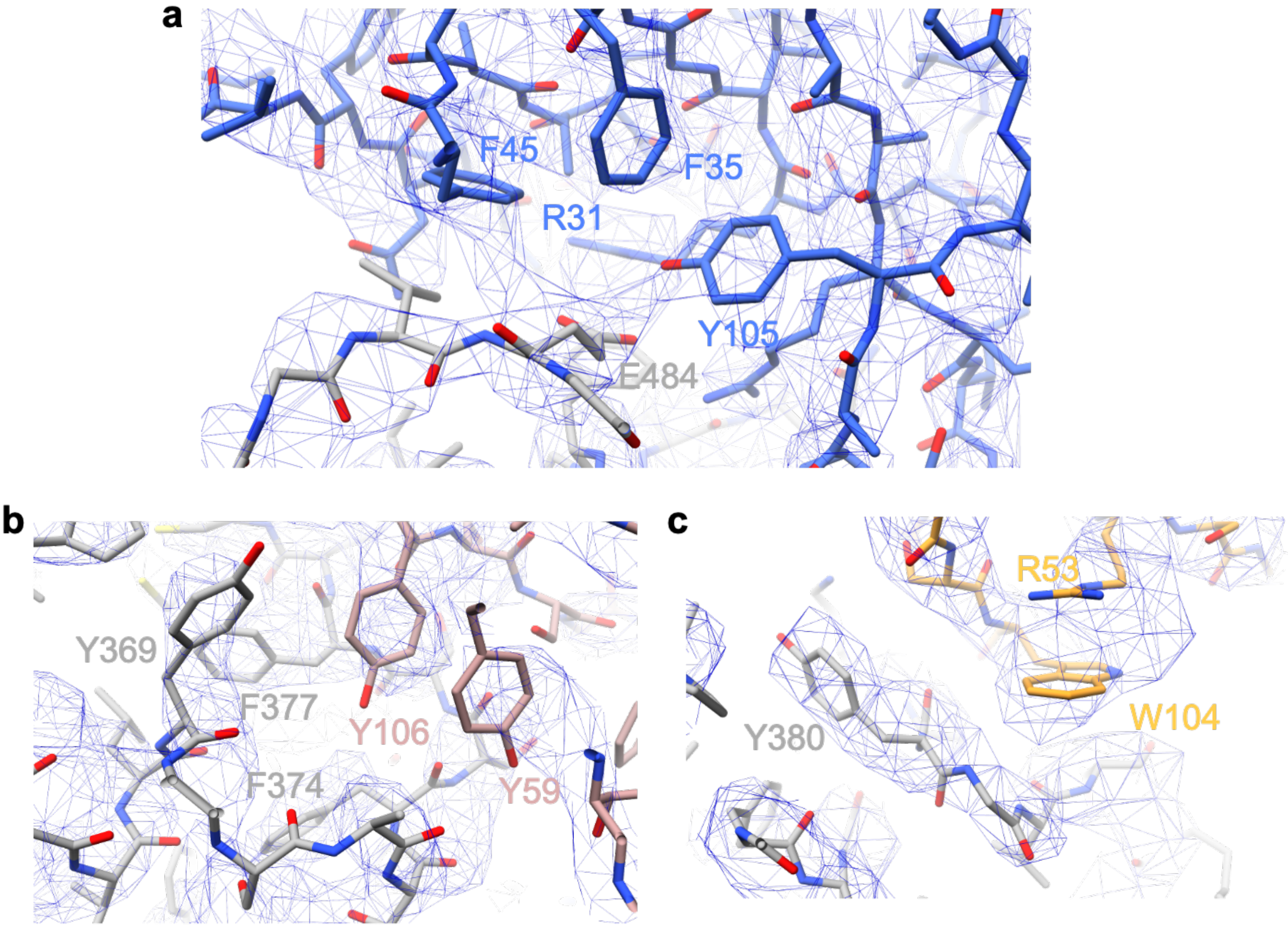

*2. There is no reference for the last sentence of paragraph 4 in page 2, 'The results contrast with recent investigations of a repertoire of neutralizing mAbs derived from convalescent and vaccine-elicited polyclonal sera, which are significantly affected by at least one of these strains.'. And to my knowledge, neutralizing mAbs from sera of both convalescent patients and vaccines were reported to remain active against B.1.1.7 lineage (see ref. Nature 2021 Mar 8.doi: 10.1038/s41586-021-03398-2).

> Thanks for pointing this out. We have modified this sentence and added the following reference:

[11]

Wang, Z., Schmidt, F., Weisblum, Y. *et al.* mRNA vaccine-elicited antibodies to SARS-CoV-2 and circulating variants. *Nature* 592, 616–622 (2021). https://doi.org/10.1038/s41586-021-03324-6

*3. Detailed information about RBD62 is needed.

> The detailed information of B62 has been added to the main text.

"B62 possesses unseen mutations which were evolved *in vitro* for high ACE2 binding affinity and potentially enhanced infectivity (Extended Data Figure 2). This highly evolved RBD mutant contains 9 point mutations (I358F, V445K, N460K, I468T, T470M, S477N, E484K, Q498R, N501Y), including both established and potential mutations that together increase the affinity of ACE2 binding by 600 fold [12]"

*4. Line 3-6 of the 6th paragraph in page 3, 'For nb95, the CDR3 side chains of D99, K100, and S108 form ionic or hydrogen-bonding interactions with the side chains of K378 and Y380 and the main chain carbonyl group of S375 of RBD, respectively (Fig. 1d). R408 … with V407, V503 and Y508 of RBD' does not match the figure 1d. For instance, polar interactions between K100 and Y380, as well as S108 and the main chain amine group of E112 were not found in figure 1d. On the contrary, an extra salt bridge between E112 and K378 was shown in figure 1d but not described in the text.

> This is much appreciated. We have revised our description and figure of the molecular details of the Nb95:RBD interface, as followed:

"For Nb95, the side chains of CDR3 residues D99 and K100 form ionic or hydrogen-bonding interactions with the side chains of K378 and Y380 of RBD, respectively (Fig. 3c). The side chain of Y55 of Nb95 also forms a hydrogen bond with the main chain carbonyl of F374 of RBD. In addition to those polar interactions, the CDR3 residues P110 and F109 of Nb95 form hydrophobic interactions with the RBD residues V503 and Y508, and residues Y55 and Y106 of Nb95 cluster with the RBD residue Y369 to form aromatic interactions (Fig. 3c)."

*5. It is better to paint the ionic and hydrogen bonds in different colors.

> We colored the ionic bonds in green and hydrogen bonds in blue throughout the figures.

Reviewer #2 (Remarks to the Author):

Sun and colleagues characterized seven nanobodies (Nbs) and found they exhibited varying levels of binding and neutralizing against a panel of SARS-CoV-2 variants. Nbs 20 and 21 were markedly affected by SA variant B.1.351 but marginally affected by UK variants B.1.1.7. The remaining five, however, remained similar potency to B.1.351. Binding analysis to mutated RBDs indicated that Nbs 20 and 21 relied on E484 while the remaining five are independent of E484, although RBD62 also disrupted binding of Nb17 and diminished binding of Nb95 and Nb36 while left Nbs 34 and 105 unaffected. The authors went further to resolved a total of 9 Nb-bound RBD/spike structures by high-resolution cryo-EM and revealed several potential antiviral mechanisms associated with epitope specificity. Accordingly, the authors categorized the seven Nbs into three classes. Class I including Nbs 20 and 21 exhibited the most potent neutralizing activity to the wildtype SARS-CoV-2 but failed to neutralize B1.351
due to E484K mutation. The other two classes bound to more conserved epitopes and maintained neutralizing activity. Class III Nbs 17 and 36 bound to previously unidentified cryptic epitopes that are inaccessible by bivalent antibodies derived from human. These results provide a fresh and comprehensive insights into how Nbs targeted the spike in a unique manner to achieve their broad neutralizing activity against the emerging variants of concerns of SARS-CoV-2.

Obviously, a timely and relevant study, with real importance for the broader community and potential clinical applications. While there have been other publications on the effect of the Variants of Concern (VOC) on Nbs neutralization, the authors here conduct a more thorough and detailed characterization on the structure basis for the observed differences in neutralizing potency and breadth.

*My one concern is the breath of Nbs claimed by the authors as they only tested two VOCs B.1.1.7 and B.1.351 where there are at least 7 VOCs have so far been identified. **It is important to test the seven Nbs against other strains so we could have better understanding on the true breadth of their neutralizing activity**.

> We greatly appreciate these comments on the novelty and impact of our study. Three dominant variants VOCs (Brazil, South Africa, and UK variants) were identified when we were preparing the manuscript. In addition, we have also evaluated the contribution of individual RBD point mutations to Nb binding and employed in vitro assay to assess the neutralization potency of two dominant VOCs.

Critical RBD (residues 319-541) mutations are shared between VOCs (bolded) and VOIs (Variants of Interest) as followed:
K417N (**B.1.351 SA)**
K417T (**P.1 Brazil**)
L452R (**B.1.427 California**, **B.1.429 California**, B.1.526.1 NY, B.1.617 India, B.1.617.1-3 India)
E484K (**B.1.1.7 UK**, **B.1.351 SA**, **P.1 Brazil**, P.2 Brazil, B.1.525 UK, B.1.526 NY**)**
E484Q (B.1.617 India, B.1.617.1 India, B.1.617.3 India)
N501Y (**B.1.1.7 UK**, **B.1.351 SA**, **P.1 Brazil**)

All VOC mutations have been assessed in our ELISA and pseudovirus experiments. Untested VOI RBD mutations include:
T478K (B.1.617.2 India)
S477N (some strains of B.1.526)

Many critical RBD mutations are shared among VOCs (e.g., Brazil and South Africa strains share key residues of K147N, E484K, and N501Y), we investigated all of these individual RBD mutants for Nb binding and two major VOCs. During the submission of our paper, two additional VOCs from California (B.1.427 and B.1.429) emerged and quickly dominated. The B.1.427 and B.1.429 contain RBD mutation L452R, which is also shared by the Variants of Interest that have emerged in India (B.1.617, B.1.617.1-3). The variants from India also contain the E484Q mutation on RBD. In the revised study, we also investigated Nb binding to the critical RBD double mutant (E484Q: L452R) from the Californian and Indian strains. The data was updated in Figure 1.

*For readers to better understand the seven Nbs, it is necessary to include a few introductory sentences as where these Nbs came from and how they were selected in the first place. What immunogen used to immunize animals and what antigen used to fish out these Nbs.

> We appreciate this comment and have added more background information about these Nbs.

" By using camelid immunization of $RBD_{SARS\text{-}CoV2}$ and an advanced proteomic pipeline, we have recently identified > 8,000 high-affinity RBD Nbs including a repertoire of ultrapotent Nbs [13]".

*Table 1, how -1000.00 or <-1000.00 were determined? What is B62? Is it RBD62? Is so, please keep the nomenclature consistent thoroughout.

> The description for Table 1 (now Fig. 1, see below) was modified to include further details of our analysis, as followed:

a. ELISA binding of the spike variants (a summary heatmap). Data shown as binding affinity fold change relative to that of RBD WT.
b.The fold change in neutralizing potencies of the Nbs against two dominant circulating variants (UK and SA strains) relative to that of the wild-type SARS-CoV-2 pseudovirus particles.
Negative values represent the loss in affinity or neutralization potency, and positive values represent a gain in affinity or neutralization potency. Based on the highest Nb concentration tested, reduction in affinity or neutralization potency greater than 1000 fold is represented as “<-1000”.

Figure 1

A

		RBD mutants							
		K417N	N439K	Y453K	A475V	E484K	N501Y	L452R E484Q	B62
Class I	Nb20	-1.21	1.13	1.21	-1.22	<-1000	-2.49	<-1000	<-1000
	Nb21	-1.43	1.18	1.04	-1.31	<-1000	-2.63	<-1000	<-1000
Class II	Nb34	-1.96	1.18	1.20	-1.00	1.07	-5.79	4.54	-2.10
	Nb105	-1.45	1.06	1.31	-1.10	1.02	-2.69	5.59	-4.10
	Nb95	1.11	-2.19	-1.31	1.21	1.15	1.37	3.50	-37.15
Class III	Nb17	-1.20	1.33	1.61	1.16	0.95	1.50	<-1000	<-1000
	Nb36	-2.94	1.43	1.32	-1.13	1.29	1.35	3.30	-145.87

B

	**B.1.1.7 (UK)**	**B.1.351 (SA)**
Nb20	-6.29	<-1000
Nb21	1.08	<-1000
Nb34	-1.14	-1.09
Nb105	-2.31	1.78
Nb95	-1.09	2.30
Nb17	1.52	-4.37
Nb36	1.12	-1.16

And yes, B62 and RBD62 are the same. Now we made the nomenclature consistent throughout the paper as B62 (RBD).

Extended Fig. 5a, how relative binding energy was calculated for each residue? In Fig.5b, why R31D was selected for analysis and what about other residues? Was the RBD used in the binding derived from the wildtype or any specific variant?

> In the revised manuscript, we included the method for the energy calculation. Briefly, the relative binding energy contribution was calculated using component analysis following the original work on the Ras-Raf complex done by Holger Gohlke et al[14]. The calculation was performed using the MMPBSA.py module available in the AMBER18 package.

Based on Nb21:S structure, we found R31 (Nb21) is likely critical for mediating the ultrahigh-affinity binding of Nb21 to RBD. R31 likely 1) forms a salt bridge with E484 (RBD) and, 2) also enables cation-pi interactions with other RBD residues such as Y104 and F490. To better understand the contribution of these two interactions to

overall binding, we made an R31**D** substitution on Nb21 to potentially restore the salt bridge interaction with the RBD E484**K** mutant. On the other hand, the R31D substitution may be electrostatically unfavorable for the cation-pi interactions with RBD residues Y104 and F490. We found that Nb21 R31D did not bind to the RBD variant (E484K), indicating the critical contribution of other cation-pi interactions that are not restored with the R31D substitution.

We clarified this in the revision.

Reviewer #3 (Remarks to the Author):

SARS-CoV-2 is evolving continuously, generating convergent variants resistant to convalescent or vaccine-elicited sera. Nanobodies (Nbs) are stable and cost-effective therapeutic option. In this work, the authors reported 9 Nb-bound structures, based on which three classes are identified. The antiviral mechanisms and neutralizing epitopes for these Nbs were analyzed and discussed. The authors also compared Nbs with other neutralizing Abs and revealed the unique characteristics for Nbs. This work provided valuable insights into the antiviral mechanisms of Nbs. The following are the points for this work.

> Thank you for the positive comments on our paper.

*1. Nb105 forms an elongated S/Nb105/S complex with two copies of the S protein. This is interesting. Why does Nb105 crosslink two copies of the S protein with only one binding interface? The authors should solve the structure for this elongated complex. Or, can the authors generate a model for the S/Nb105/S complex based on the binding interface between Nb105 and RBD?

> We have docked the isolated Nb105-RBD structure and the open spike (PDB entry: 7CAK) into this elongated dimeric spike trimer complex. We have also performed molecular dynamic flexible fitting (MDFF) to better understand the interface. Our analysis reveals two potential binding interfaces of Nb105. The first interface (highlighted in a red line) is formed between Nb105 CDR3 and the corresponding RBD epitope. This interface is specific. The second interface is formed between the Nb105 framework and the ACE2 binding sites. The interaction (highlighted in green) is likely mediated by nonspecific ionic bonds. We have modified our previous panel f in Extended Data Fig. 8 to clearly delineate these two distinct interfaces as well as in the text to clarify this point.

F

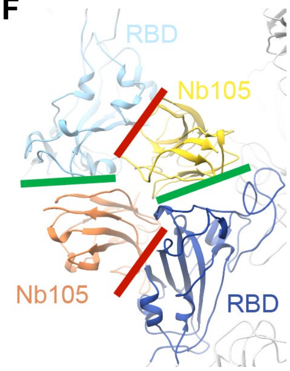

2. The map quality of some cryo-EM maps is poor and should be improved, including the focused refined maps on the interface between Nb95 or Nb34 and RBD, the maps of Nb21/RBD/Nb105 and Nb17/RBD/Nb105.

> As suggested by the reviewer, we have performed additional refinements of local maps for the RBD and Nb complexes. For Nb34, the quality of local maps is poor and we didn’t discuss the molecular details of the interface. We have also included figures showing local maps of interfaces between Nbs and RBD(now in Extended Data Fig. 10).

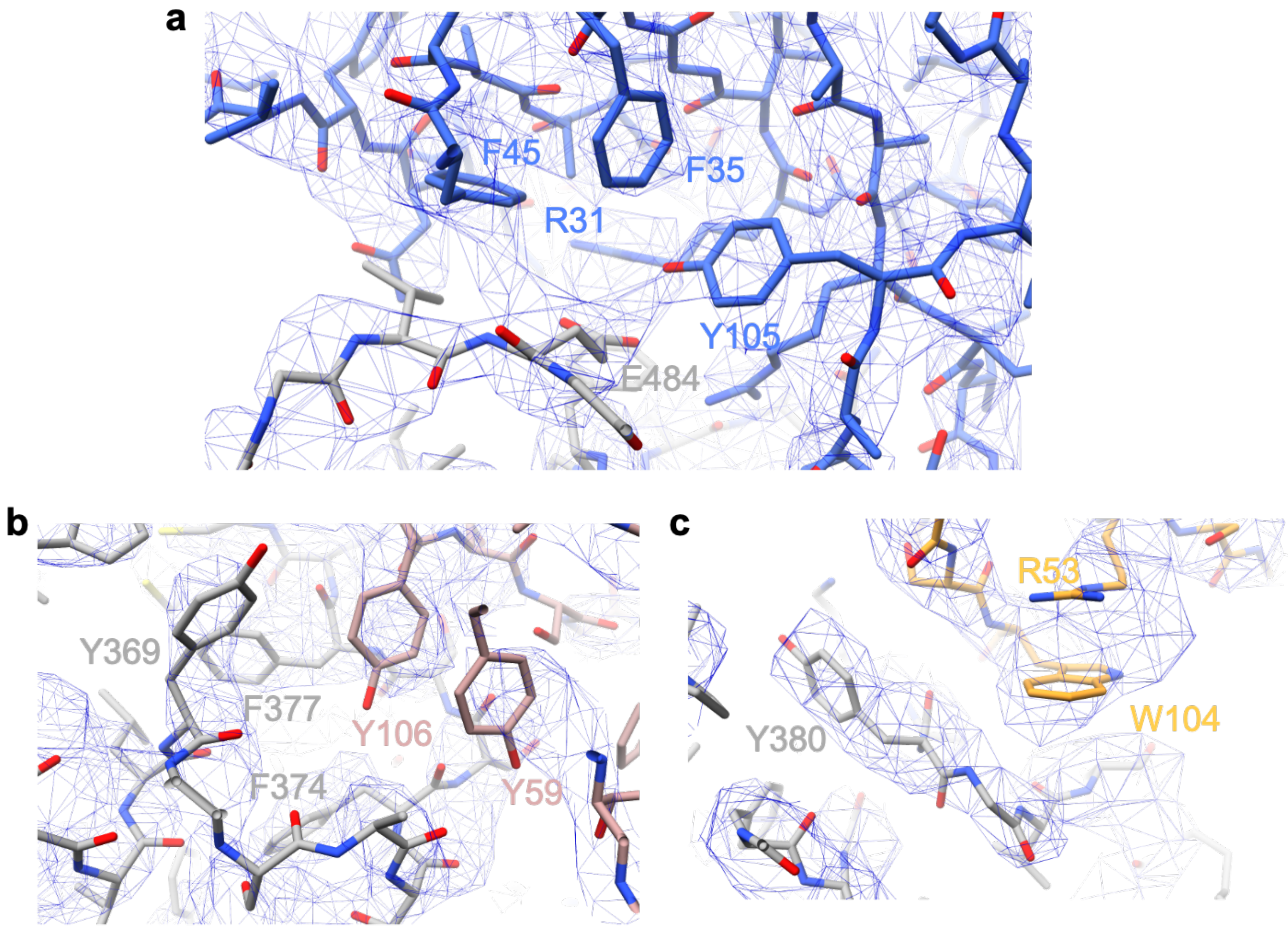

*3. Page 4, “While class II Nbs do not interact with the ACE2 directly, they can still efficiently block ACE2 binding at low nM concentrations (Fig. 2h).” I think this should be “While class II Nbs do not compete with the ACE2 directly … ”

> Corrected.

*4. For Fig 1 legend, please explain the meaning of the minus fold change.

> The description for Figure 1 was modified to include further details of our analysis, as followed:

a. ELISA binding of the spike variants (a summary heatmap). Data are shown as binding affinity fold change relative to that of RBD WT.

b. The fold change in neutralizing potencies of the Nbs against two dominant circulating variants (UK and SA strains) relative to that of the wild-type SARS-CoV-2 pseudovirus particles.
Negative values represent a loss in affinity or neutralization potency, and positive values represent a gain in affinity or neutralization potency. Based on the highest Nb concentration tested, reduction in affinity or neutralization potency greater than 1000 fold is represented as “<-1000”.

1 Dong, G. Q., Fan, H., Schneidman-Duhovny, D., Webb, B. & Sali, A. Optimized atomic statistical potentials: assessment of protein interfaces and loops. *Bioinformatics* **29**, 3158-3166, doi:10.1093/bioinformatics/btt560 (2013).
2 Moal, I. H., Jimenez-Garcia, B. & Fernandez-Recio, J. CCharPPI web server: computational characterization of protein-protein interactions from structure. *Bioinformatics* **31**, 123-125, doi:10.1093/bioinformatics/btu594 (2015).
3 Kastritis, P. L. & Bonvin, A. M. Are scoring functions in protein-protein docking ready to predict interactomes? Clues from a novel binding affinity benchmark. *J Proteome Res* **9**, 2216-2225, doi:10.1021/pr9009854 (2010).
4 Ashkenazy, H. *et al.* ConSurf 2016: an improved methodology to estimate and visualize evolutionary conservation in macromolecules. *Nucleic Acids Res* **44**, W344-350, doi:10.1093/nar/gkw408 (2016).
5 Zhou, H. *et al.* Structural definition of a neutralization epitope on the N-terminal domain of MERS-CoV spike glycoprotein. *Nat Commun* **10**, 3068, doi:10.1038/s41467-019-10897-4 (2019).
6 Walls, A. C. *et al.* Unexpected Receptor Functional Mimicry Elucidates Activation of Coronavirus Fusion. *Cell* **176**, 1026-1039 e1015, doi:10.1016/j.cell.2018.12.028 (2019).
7 Piccoli, L. *et al.* Mapping Neutralizing and Immunodominant Sites on the SARS-CoV-2 Spike Receptor-Binding Domain by Structure-Guided High-Resolution Serology. *Cell* **183**, 1024-1042 e1021, doi:10.1016/j.cell.2020.09.037 (2020).
8 Hsieh, C. L. *et al.* Structure-based design of prefusion-stabilized SARS-CoV-2 spikes. *Science* **369**, 1501-1505, doi:10.1126/science.abd0826 (2020).
9 Yuan, M. *et al.* A highly conserved cryptic epitope in the receptor binding domains of SARS-CoV-2 and SARS-CoV. *Science* **368**, 630-633, doi:10.1126/science.abb7269 (2020).
10 Huo, J. *et al.* Neutralization of SARS-CoV-2 by Destruction of the Prefusion Spike. *Cell Host Microbe* **28**, 445-454 e446, doi:10.1016/j.chom.2020.06.010 (2020).
11 Wang, Z. *et al.* mRNA vaccine-elicited antibodies to SARS-CoV-2 and circulating variants. *Nature* **592**, 616-622, doi:10.1038/s41586-021-03324-6 (2021).
12 Zahradník, J. *et al.* SARS-CoV-2 RBD in vitro evolution follows contagious mutation spread, yet generates an able infection inhibitor. *bioRxiv*, 2021.2001.2006.425392, doi:10.1101/2021.01.06.425392 (2021).
13 Xiang, Y. *et al.* Versatile, Multivalent Nanobody Cocktails Efficiently Neutralize SARS-CoV-2. *bioRxiv*, doi:10.1101/2020.08.24.264333 (2020).
14 Gohlke, H., Kiel, C. & Case, D. A. Insights into protein–protein binding by binding free energy calculation and free energy decomposition for the Ras–Raf and Ras–RalGDS complexes. *Journal of molecular biology* **330**, 891-913 (2003).

REVIEWER COMMENTS

Reviewer #1 (Remarks to the Author):

All my concerns have been addressed.

Reviewer #2 (Remarks to the Author):

The authors have satisfactorily addressed all my concerns.

Reviewer #3 (Remarks to the Author):

The authors have well addressed the issues raised in previous review round. The current manuscript has been much improved and suitable for acceptance after addressing the following point:

"Consistent with epitope conservation analysis, the ELISA results show that unlike class I and III Nbs, potent neutralizing class II Nbs (specifically, Nb95 and Nb105, but not Nb34) bind strongly to RBD_{SARS-CoV-2} by targeting highly conserved RBD epitopes (Extended Data Fig. 13c-d)." The authors should double check this sentence. The RBD_{SARS-CoV-2} should be RBD_{SARS-CoV} according to my understanding.

Reviewer 3

“Consistent with epitope conservation analysis, the ELISA results show that unlike class I and III Nbs, potent neutralizing class II Nbs (specifically, Nb95 and Nb105, but not Nb34) **bind strongly to $RBD_{SARS-CoV-2}$** by targeting highly conserved RBD epitopes (Extended Data Fig. 13c-d).” The authors should double check this sentence. The $RBD_{SARS-CoV-2}$ should be $RBD_{SARS-CoV}$ according to my understanding.”

We thank the reviewer for pointing out this typo! The sentence was modified as **“bind strongly to both $\underline{RBD_{SARS-CoV}}$** and **$RBD_{SARS-CoV-2}$** ” in the final version of the manuscript.
